# Rational Design of PROTAC Linkers Featuring Ferrocene as a Molecular Hinge to Enable Dynamic Conformational Changes

**DOI:** 10.1021/jacs.4c18354

**Published:** 2025-04-10

**Authors:** Alessandra Salerno, Lianne H. E. Wieske, Claudia J. Diehl, Alessio Ciulli

**Affiliations:** Centre for Targeted Protein Degradation, School of Life Sciences, University of Dundee, 1 James Lindsay Place, Dundee DD1 5JJ, U.K.

## Abstract

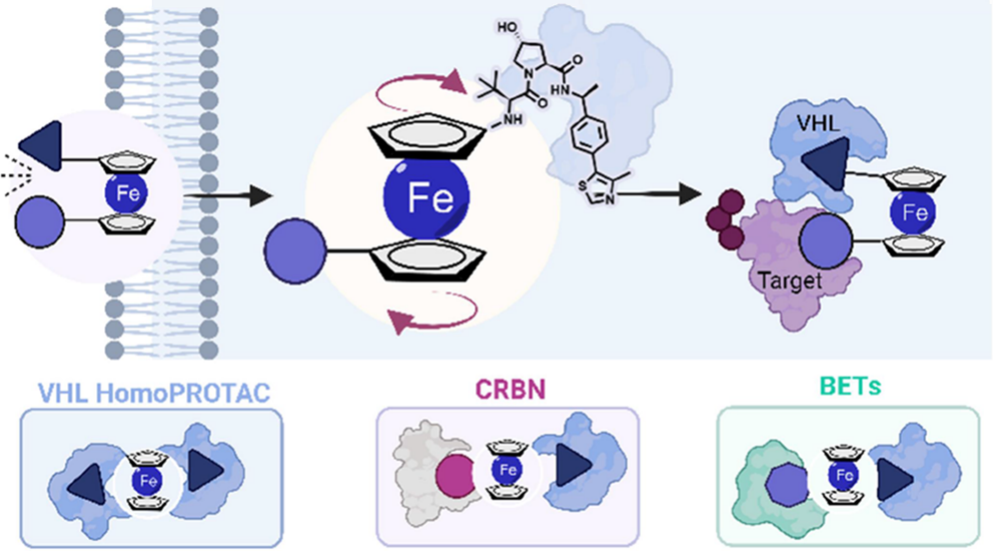

Proteolysis Targeting Chimeras (PROTACs) are bifunctional molecules that induce ubiquitination and degradation of a target protein via recruitment to an E3 ligase. The linker influences many steps of the PROTAC mode of action, from cellular permeability to ternary complex formation and target degradation. Much interest has therefore been devoted to linker design to fine-tune molecular and mechanistic properties of PROTACs. In this study, we present FerroTACs, a novel PROTAC design strategy incorporating ferrocene as the linker chemotype. We exemplify the approach across three different PROTAC systems: VHL-VHL (homo-PROTACs), VHL-CRBN, and VHL-BETs. We find that ferrocene’s unique organometallic structure, featuring freely rotating cyclopentadienyl rings around a central Fe(II) ion, acts as a molecular hinge enabling structural adjustment to the environment that results in properties alteration, i.e., chameleonicity. Conformational analyses via NMR spectroscopy support ferrocene’s role in fostering intramolecular interactions that result in a more folded state in an apolar environment. This property promotes compact conformations, improving cellular permeability and reducing efflux liabilities. Cellular assays demonstrate that FerroTACs exhibit robust target degradation and cell permeability profiles, en-par or enhanced compared to benchmark PROTACs **CM11**, **14a**, and **MZ1**. These findings highlight ferrocene’s potential as a new linker design strategy, offering a versatile platform to install and control molecular chameleonicity into next-generation PROTACs.

## Introduction

**Figure 1:**
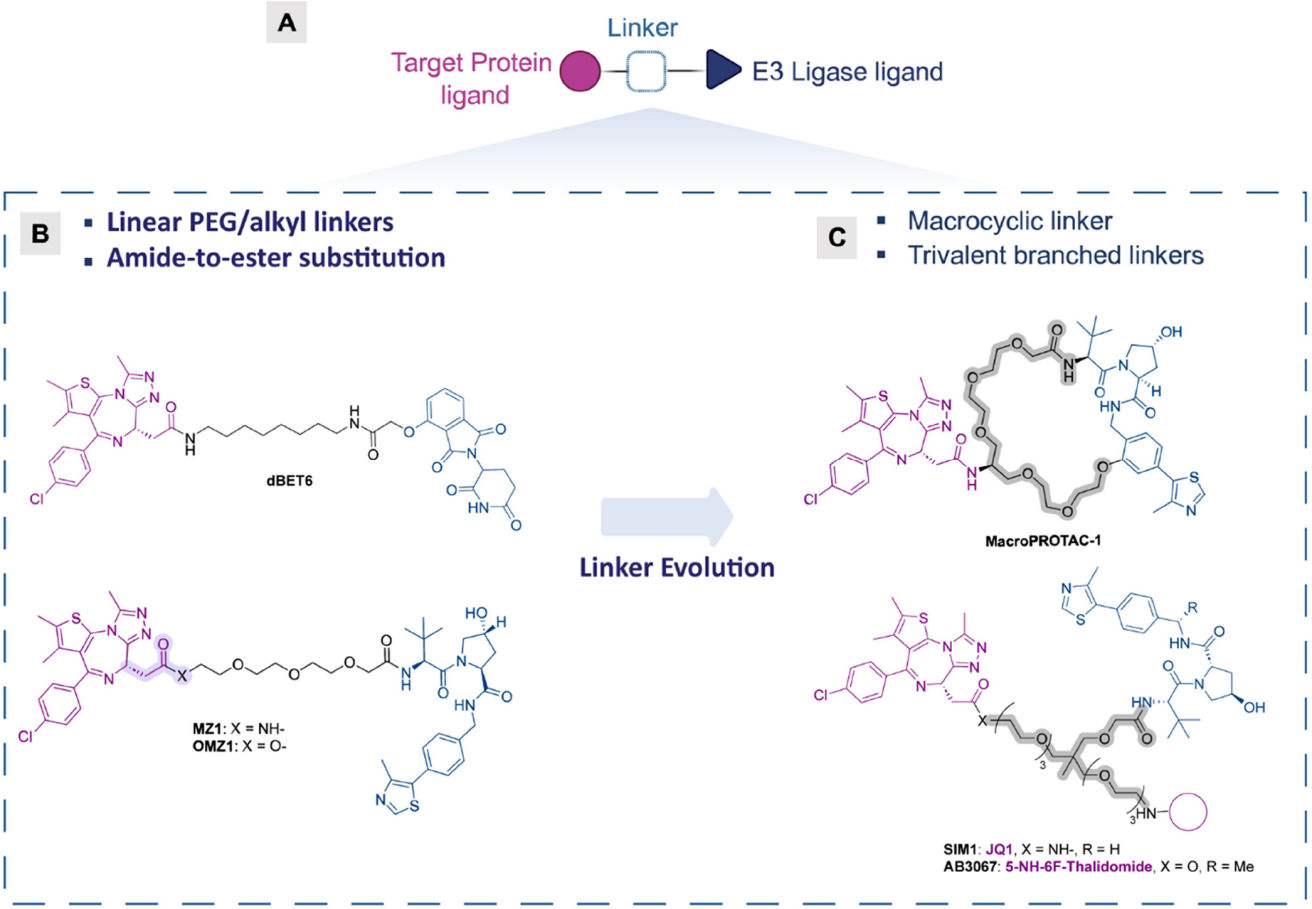
**Representative examples of PROTAC linkers.** (A) General structure of a PROTAC consisting of a ligand for the target protein, a linker, and a ligand for the E3 ligase. (B) Traditional aliphatic linkers (**dBET6**^7^), PEG-based linkers (**MZ1**^6^) and amide-to-ester substitution (**OMZ1**^17^). (C) Representative examples of linkers incorporating innovative modalities, such as macrocycles (**MacroPROTAC-1**^13^), and novel chemical architectures, including trivalent branched linkers (**SIM1**^14^ and **AB3067**(15)).

Targeted protein degradation is an innovative strategy in chemical biology and drug discovery that leverages the induced proximity of a ubiquitin E3 ligase to a target protein, facilitating the polyubiquitination and subsequent degradation of the target. This pharmacological strategy employs bifunctional molecules known as Proteolysis Targeting Chimeras (PROTACs), which work via a catalytic, substoichiometric mechanism to eliminate disease-causing proteins from the cell. This mechanism of action distinguishes PROTACs from traditional inhibitors, which block a protein’s activity by binding to its functional site, offering advantages such as reduced dosing requirements and prolonged effects.^1,2^ PROTACs comprise a ligand that binds to a target protein and another that recruits the E3 ubiquitin ligase, connected by a linker (Figure 1A). The linker plays a critical role in bringing the target protein and the ligase into the suitable proximity to form a stable and favorable ternary complex. This enables high potency and selectivity of ligase-directed target ubiquitination and subsequent degradation.^3^

The design and length of the linker are crucial, as these parameters not only impact ternary complex formation but ultimately influence the PROTAC’s physicochemical properties. Recent efforts have increasingly focused on understanding and optimizing PROTAC linkers to unlock their full therapeutic potential. The growing body of research and publications from both drug discovery and academic settings highlights the central role of the linker in PROTAC design and refinement, a field of study often referred to as “linkerology.”^4,5^ This discipline involves careful consideration of factors such as linker chemical composition, length, and flexibility. Historically, PROTAC linkers have been composed of linear PEG (i.e., **MZ1**,^6^Figure 1B) or alkyl chains (i.e., **dBET6**,^7^Figure 1B) of varying lengths. However, recent advances have introduced more complex linker designs, including rigid cyclic structures, heterocyclic scaffolds, spiro and bridged rings, alkynes, ionizable tertiary amines, fluorine atoms, and chiral centres.^8−11^ These unconventional motifs are being investigated not only to enhance PROTAC activity but also to fine-tune their pharmacokinetic properties, including solubility, lipophilicity, intracellular and metabolic stability, and oral bioavailability.^12^ Similarly, the development of creative and diversified linkers, including the integration of other chemical modalities like macrocycles (i.e., **MacroPROTAC-1**,^13^Figure 1C), or multivalent chemical architectures (i.e., **SIM1**(14) and **AB3067**,^15^Figure 1C) is also gaining attention. Linkerology allows the optimisation of the properties and activity of degraders, while also offering a fertile ground for medicinal chemistry experimentation to address current PROTACs limitations.^16^

A significant challenge for PROTACs development remains their poor cellular permeability/uptake and proneness to transporter-mediated efflux, usually stemming from their high molecular weight and large exposed polar surface area (PSA) due to linear structures. To tackle this issue, we and others have explored strategies including enhancing linker lipophilicity, replacing amides with esters (i.e., **OMZ1,**(17)Figure 1B) and altering linker composition to influence the conformational dynamics of PROTACs.^18^ Indeed, specific linker designs can enable PROTACs to act as molecular chameleons, adopting more compact conformations in response to environmental conditions.^10,19−21^ This molecular feature has the potential to change compounds’ drug-like properties, thereby improving their cellular permeability and reducing the efflux ratio.^21−23^ While the concept of molecular chameleons was introduced in the 1970s, interest in this concept has grown significantly as drug discovery shifts toward novel chemical modalities.^24,25^ These modalities, such as PROTACs, exist in a chemical space beyond traditional small-molecule drugs and the rule of five, and their drug-like properties are partially attributed to their “molecular chameleon” properties, enabling them to adapt different conformations in diverse environments.^26^

In this work, we present the development of ferrocene-PROTACs (FerroTACs), a novel strategy to bias PROTAC chameleonicity through the rational incorporation of an unconventional linker motif. Central to this design is the use of ferrocene, an organometallic moiety with distinctive structural and functional properties.

## Results and Discussion

### FerroTACs Design Rationale

**Figure 2:**
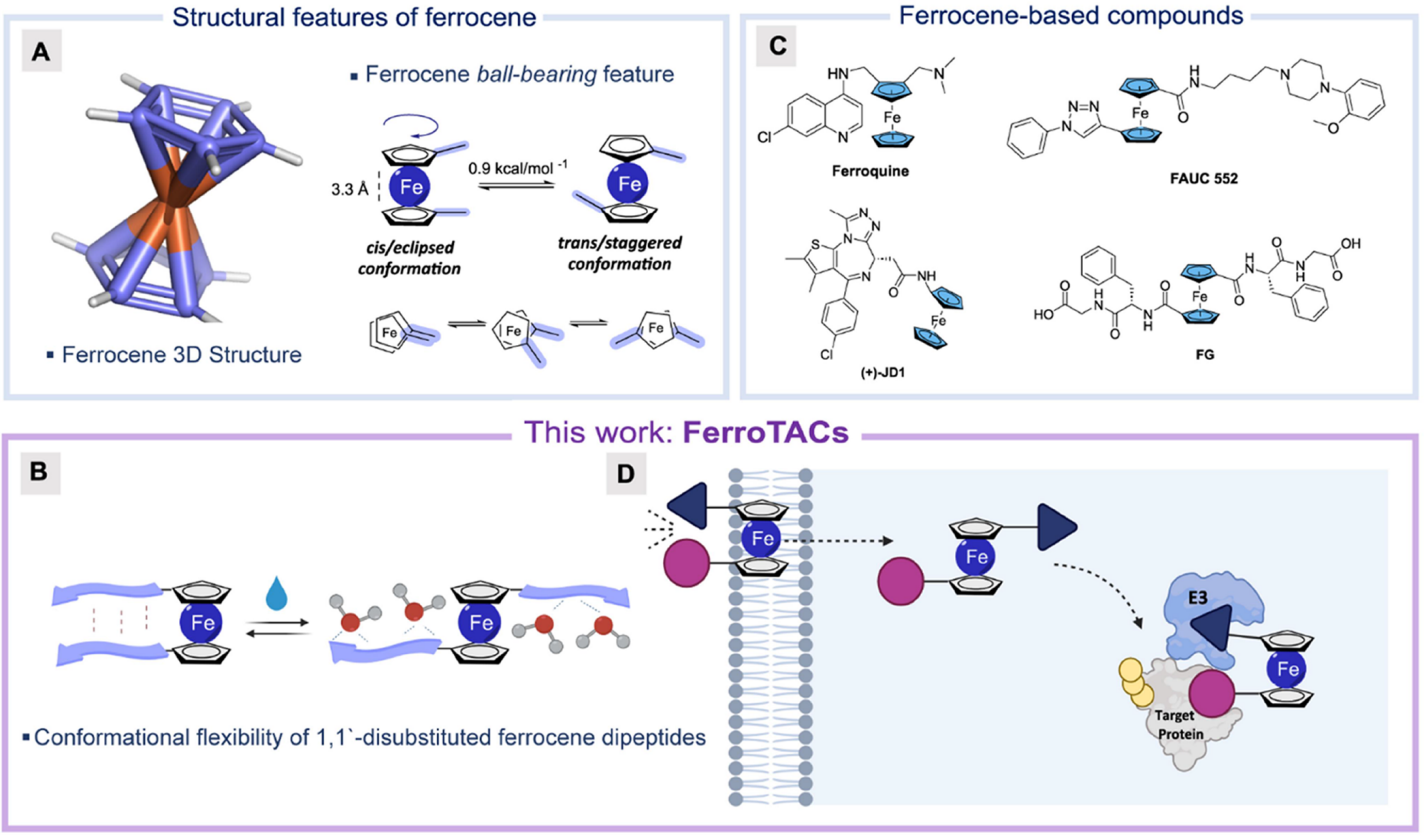
**Structural insights and applications of ferrocene.** (A) Ferrocene 3D structure (CSD, refcode: FEROCE04), *atomic ball-bearing feature,* and conformational flexibility behavior. (B) Overview of the general structure of 1,1′-disubstituted ferrocene dipeptides and their water-mediated conformational change (Adapted from *J. Phys. Chem. Lett.***2021**, 12, 26, 6190–6196. Copyright 2021 American Chemical Society). (C) Representative structures of compounds integrating a ferrocene moiety (i.e., ferroquine^35^ and (+)-JD1,^33^*left*) or having ferrocene as central linker moiety (FAUC 552^37^ and FG^29^, *right*). (D) Graphical representation of FerroTACs. In these structures, the ferrocene moiety could function as a molecular hinge, enhancing the chameleonic behavior of PROTACs by providing adaptable conformational flexibility.

Discovered serendipitously in 1951, ferrocene (Fc) features a sandwich-like structure with two parallel cyclopentadienyl (Cp) rings that share the π-electrons with the central Fe(II) ion via covalent bonding (Figure 2A).^27^ Consequently, ferrocene does not release free iron ions under normal conditions, as the iron is securely bound within the molecular framework. In the ferrocene structure, the Fe(II) ion acts as an “atomic ball-bearing” that enables the Cp rings to rotate freely in solution (Figure 2A). This allows ferrocene-based structures to dynamically change their conformation through thermal rotation with a low energy barrier of just 0.9 kcal mol^–1^.^28^ The preferred conformation of ferrocene derivatives, *staggered* and *eclipsed* herein referred as *cis* and *trans*, respectively, depends on various factors. In structures like 1,1′-disubstituted ferrocenes, the short distance between the Cp ring planes (3.3 Å) brings lateral substituents close together, facilitating supramolecular interactions such as intramolecular hydrogen bonding and π–π interactions, which could favor the *cis* conformation. These interactions are generally weak, and external factors can induce controllable switching between conformations (Figure 2A,B).^29,30^ For example, in ferrocene-conjugated dipeptides, the side chains formed a parallel β-sheet that shifted from *cis* to *trans* in aqueous media, a behavior linked to hydration and that results in hydrogen bond rearrangement between the side chains (Figure 2B). Ferrocene is an important structural core in (bio)organometallic chemistry because of its inherent stability, excellent redox properties, and tunable toxicity, making it a versatile platform in various fields, including nanotechnology, catalysis and medicinal chemistry.^31−34^ The lipophilic nature makes ferrocene a particularly attractive motif, especially for incorporation in bioactive compounds to modulate the overall lipophilicity. For instance, ferroquine (Figure 2C), now in phase II of clinical trials, is one of the most notable contributions of ferrocene to medicinal chemistry with remarkable improved lipophilicity and antimalarial properties.^35,36^

Inspired by the peculiar molecular properties of ferrocene, we envisaged its potential applications into linkers for bifunctional molecules. We hypothesized that the ferrocene scaffold could enable controlled modulation of the conformational landscape, via “chameleonicity” as a strategy to modulate physicochemical properties, and we aimed to investigate and exemplify this concept with PROTACs (Figure 2D).

**Figure 3:**
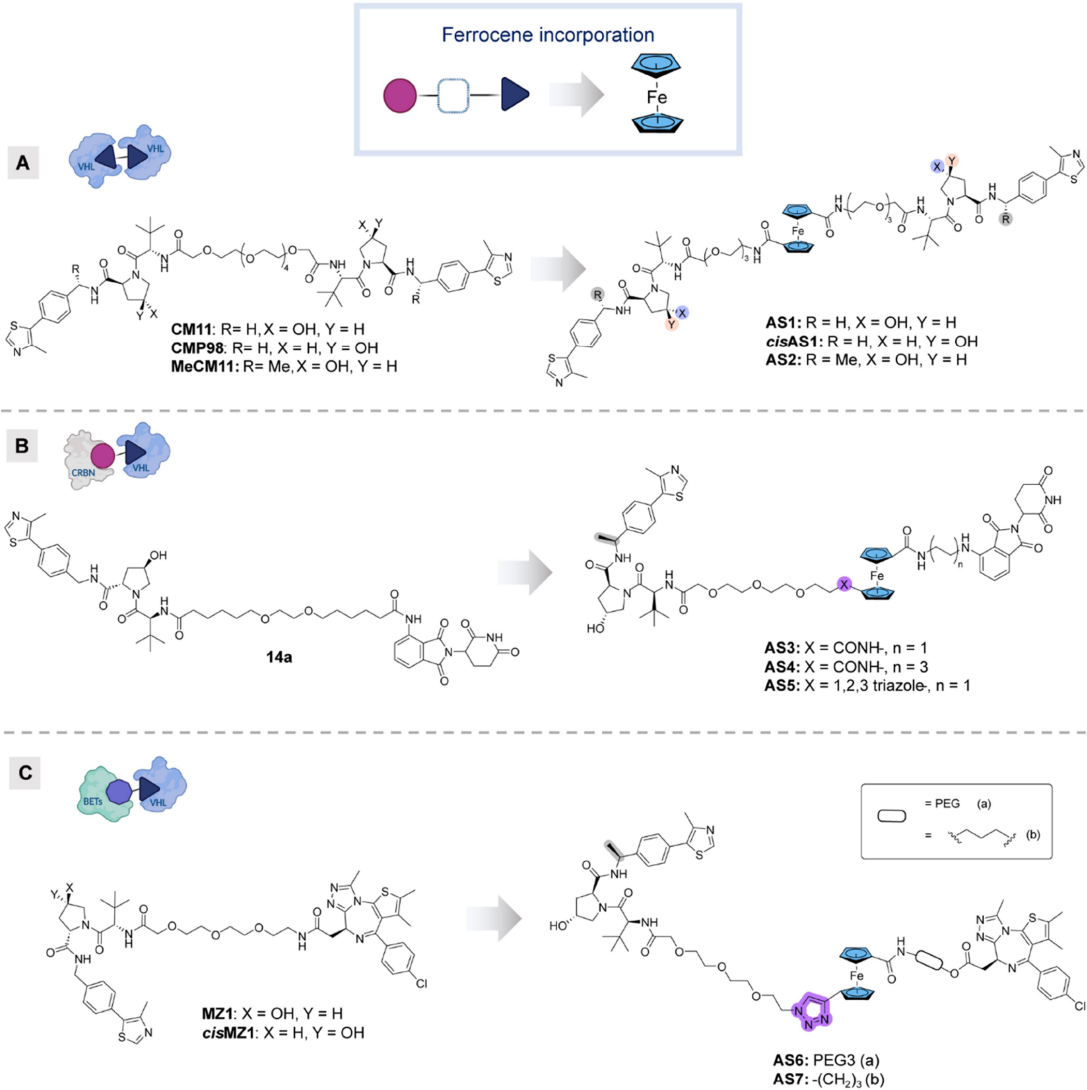
**Design of FerroTACs AS1–AS7 incorporating ferrocene as a linker moiety.** (A) Homo-FerroTACs **AS1**, *cis***AS1**, and **AS2** targeting VHL, and reference compounds **CM11**, **CMP98**, and **MeCM11**. (B) FerroTAC **AS3-AS5** targeting CRBN system, and reference compound **14a**; (C) FerroTACs **AS6** and **AS7** targeting BETs, and reference compounds **MZ1** and *cis***MZ1**.

To explore the potential of ferrocene as a versatile strategy for PROTAC linker development, we designed and synthesized three series of FerroTACs by incorporating the metallocene across three distinct systems, based on previously known structures of Fc-free homo- and hetero-PROTACs. We templated our homo-PROTAC system design based on the VHL dimerizer degrader **CM11**(38) (Figure 3A). We also utilized heterobifunctional PROTACs linking VHL and CRBN—building on our own previous work (e.g., **14a**,^39^Figure 3B). Additionally, we explored PROTACs aimed at degrading BET family proteins by recruiting VHL, exemplified by PROTAC **MZ1**(6) (Figure 3C).

Our goal in designing FerroTACs was to minimize the disruption from the newly introduced ferrocene moiety, by preserving approximately the same linker length and number of amide bonds as in the reference compounds whenever feasible. Additionally, all the developed FerroTACs are neutral species, which represents a significant advantage for the study and contributes to enhance their potential applications. VHL-targeting homo-FerroTACs **AS1** and *cis***AS1** (Figure 3A) were synthesized by incorporating ferrocene into the reference structures of **CM11** and its nondegrading analogue **CMP98**, respectively. Given the evidence that a benzylic methylated VHL ligand (MeVH032) demonstrated a significant increase in binding affinity and proved a favorable modification as part of PROTACs,^40^ we also included **CM11**’s benzyl-methylated analogue (**MeCM11**) from our previous work,^41^ resulting in the design of the VHL-targeting FerroTAC **AS2.** Building on MeVH032, we developed the CRBN-VHL-targeting hetero-PROTACs **AS3** and **AS4** (Figure 3B) from a ferrocene dicarboxylic precursor. Both structures incorporated linkers of varying lengths (i.e., alkyl C-2 and C-6 and PEG3) on each side of the ferrocene motif. Furthermore, to reduce the number of hydrogen bond donors (HBD) and enhance the feasibility of asymmetrical chemical derivatization, we employed an alternative strategy by introducing an alkyne as a chemical handle for click chemistry on the ferrocene building block resulting in the incorporation of a 1,2,3-triazole as amide bioisostere (Figure 3B,C). Using click chemistry, we synthesized 1,1-disubstituted ferrocenes with a triazole linkage for CRBN/VHL (**AS5**) and BET-targeting analogues containing JQ1: **AS6**, which incorporates a PEG3 linker, and **AS7**, featuring a shorter C-3 alkyl linker on the JQ1 side (Figure 3C).

### FerroTACs Synthesis

**Scheme 1:**
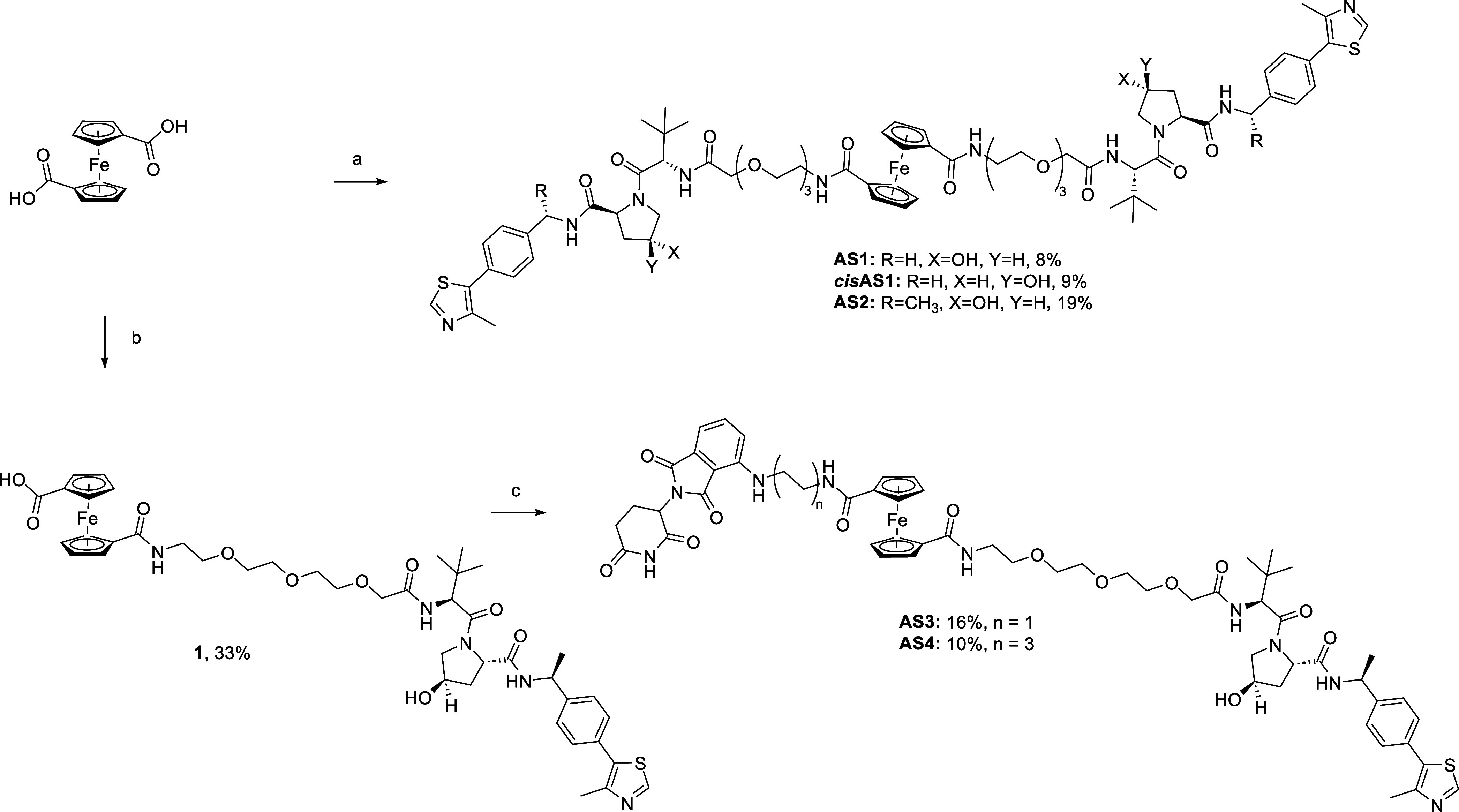
Synthesis of Homo-FerroTACs AS1, *cis*AS1 and AS2 and Hetero-FerroTACs AS3 and AS4 by Amide Connection. Reagents and conditions: (a) (i) SOCl_2_, TEA, dry DCM, 0 °C to r.t., 2 h; (ii) **7a, 7b**, or **7c**, TEA, DMAP, dry DCM, r.t., overnight (8–19%); (b) **7c**, HATU, DIPEA, dry DCM, r.t., overnight (33%); (c) **8** or **9**, HATU, DIPEA, dry DCM, r.t., overnight (10–16%).

After preparing the VHL ligands (VH032, MeVH032, and *cis*VH032) and required linkers (Scheme S1), the homo-FerroTACs were assembled around the 1,1′-dicarboxylic ferrocene core in a convergent manner, as depicted in Scheme 1.

The 1,1′-ferrocenedicarboxylic acid was converted to its corresponding acyl chloride and combined with the appropriate amines **7a**–**c** to give **AS1**, *cis***AS1**, and **AS2,** respectively. A different synthetic approach was used for the hetero-FerroTACs **AS3** and **AS4**, as shown in Scheme 1. Asymmetric monofunctionalization was achieved through HATU-mediated amide coupling with an excess of 1,1′-ferrocenedicarboxylic acid. The resulting intermediate **1** underwent further amide coupling with the corresponding amino-pomalidomide derivatives **8** and **9**, yielding compounds **AS3** and **AS4** in moderate yields.

**Scheme 2:**
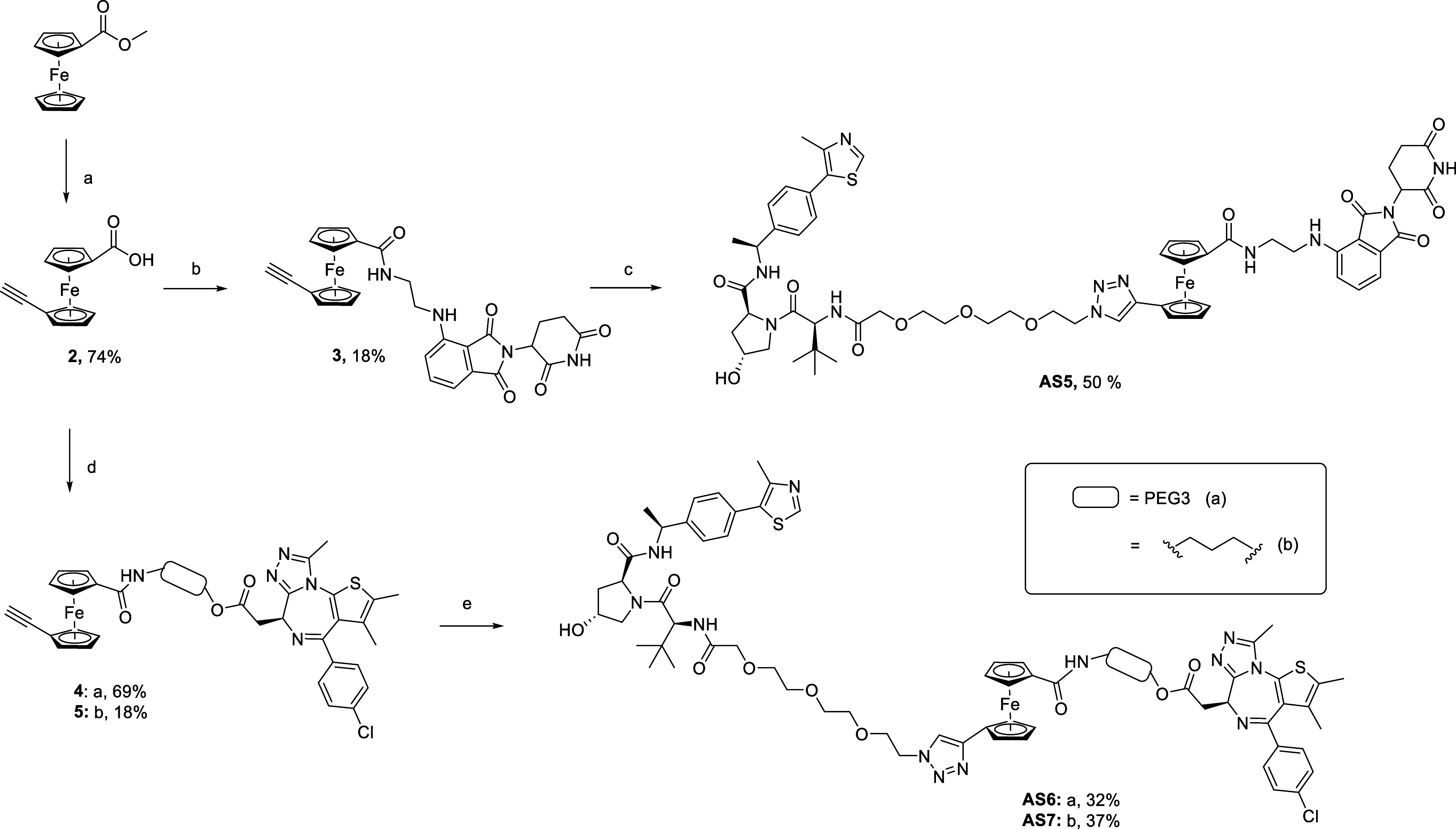
Synthesis of Hetero-FerroTACs AS5, AS6 and AS7 by Click Chemistry Reagent and conditions: (a) (i) acetylchloride, AlCl_3_, dry DCM, 0 °C, 4 h, (ii) POCl_3_, dryDMF, 0 °C to r.t., 2 h, (iii) dry dioxane, reflux, 5 min;0.5 N NaOH, reflux, 25 min (74%); (b) **8**, HATU, DIPEA, DCM,r.t. overnight (18%); (c) **6c**, CuSO_4_, NaAsc, H_2_O, tBuOH,DCM, r.t., overnight (50%); (d) **10** or **11** HATU, DIPEA, DCM, r.t,overnight (18–69%); (e) **6c**, CuSO_4_, NaAsc, H_2_O, tBuOH,DCM, r.t., overnight (32–37%).

To facilitate the asymmetric functionalization and synthesis of hetero-FerroTACs (Scheme 2), we introduced an alkyne handle on the ferrocene core following an established literature procedure.^37^ The ferrocene building block was synthesized starting from the methyl ferrocene carboxylate to produce the 1,1′-carboxy-ethynylferrocene **2** in 74% overall yield, through Friedel–Crafts acylation followed by a Vilsmeier-type formylation and a final elimination step. After activation with HATU and subsequent addition of pomalidomide-(**8**) and JQ1-amino-derivatives (**10** and **11**, Scheme S1), the intermediates **3**, **4**, and **5** were obtained, respectively. Using a copper(I)-catalyzed azide–alkyne cycloaddition (CuAAC) protocol, 1,2,3-triazole moieties were formed as linker connections by reacting VHL-PEG3-azido **6c** with the previously described ferrocenylethynes yielding compounds **AS5**, **AS6**, and **AS7**, respectively.

### NMR Conformational Studies

To explore how the rotational flexibility of ferrocene could impact the dynamic conformational changes of those compounds, we performed proton nuclear magnetic resonance (^1^H NMR) studies to gain more structural insights into FerroTACs in solution. To date, several techniques are available to study a compound’s ability to adapt its conformation to the local environment. For example, X-ray crystallography offers structural insights but is limited in throughput and environmental relevance due to the solvent used. Physicochemical property measurements can provide valuable information but are independent of the environment. Chromatographic methods such as ChameLogD^42^ and ChamelogK^43^ assess compound adaptability without the need for a reference compound, though they do not directly reveal the conformational details. In our view, NMR remains the most effective technique that allows to capture structural insights at atomic-level, enabling detailed analysis of conformational behavior. However, for NMR studies of highly flexible molecules like PROTACs, the chemical properties and flexibility of linkers are crucial in shaping their behavior in solution. Such molecules generally exist as conformational ensembles, featuring diverse arrays of distinct conformers stabilized by weak intramolecular interactions. The dynamic flexibility of the linker allows for rapid interconversion of these conformers on the timescale of the NMR experiments time scale, leading to the observation of only an averaged NMR spectrum.^44^ Although the use of NAMFIS^45^ (NMR analysis of molecular flexibility in solution) algorithm to deconvolute time-averaged NMR data into distinct solution ensembles was a potential established option among the available NMR approaches,^22,46^ we had to discard it due to significant FerroTAC signal overlap, which prevented reliable data deconvolution.

With this premise, we performed NMR studies to quantify the HBD solvent exposure of the amide protons using the HBD Acidity NMR score (A_NMR_)^47^ and variable temperature studies (vt-NMR).^48^ To note, although vt-NMR has originally been developed and applied to the study of peptides,^49^ it is, together with the A_NMR_ value, a well-established method for studying the intramolecular hydrogen bonding (IMHB) patterns in systems such as PROTACs^10,12,18,26^ as well as in ferrocene structures.^29,50−53^

^1^H NMR spectroscopy experiments were conducted to investigate the intramolecular dynamics of FerroTAC **AS4** and its closest Fc-free linear analogue, **14a**. **AS4** was chosen for the conformational study because (i) it bears two different substitutes (CRBN and VHL ligands) and (ii) its two amide groups (N(3)-H and N(4)-H) are positioned near the ferrocene moiety, which facilitates monitoring of conformational changes compared to other FerroTACs in our library containing a 1,2,3-triazole group or the symmetric homo-FerrTACs. [For the full NMR characterization, please refer to the SI, Figures S1–S3, Table S1]. Specifically, we used NMR spectroscopy to determine the ensemble-averaged properties of **AS4** in solvents having different polarity (i.e., CDCl_3_ and methanol-*d*_*4*_ or DMSO-*d*_*6*_) and hydrogen bonding properties (i.e., methanol-*d*_*4*_ or DMSO-*d*_*6*_). CDCl_3_ was chosen to examine the behavior of the FerroTAC in an apolar environment. Its dielectric constant (ε = 4.8) closely approximates that of a lipid bilayer (ε = 3.0), making it an effective model for simulating the interior environment of a cell membrane.^54^ While water (ε = 78.5) would have been the preferred solvent to simulate extracellular and intracellular environments, DMSO-*d*_*6*_ and methanol-*d*_*4*_ were used instead because of the poor compound solubility. Methanol-*d*_*4*_ has a relatively high dielectric constant (ε = 32.7) and offers the added advantage of acting as a hydrogen-bond donor, unlike the more commonly used DMSO-*d*_*6*_ (ε = 46.7). Although DMSO-*d*_*6*_ has drawbacks such as higher viscosity and lack of hydrogen-bond donor capability, it serves as a strong hydrogen-bond acceptor, effectively disrupting weak hydrogen bonds and is the most commonly used solvent in reference literature.^47^ We thus chose to use DMSO-*d*_*6*_ to benchmark the data from vt-NMR and A_NMR_ experiments and prioritise the use of protic solvent (methanol-*d*_*4*_), resembling the hydrogen-bonding capabilities of water despite its slightly lower dielectric constant*_,_* for NOESY analysis.

**Figure 4:**
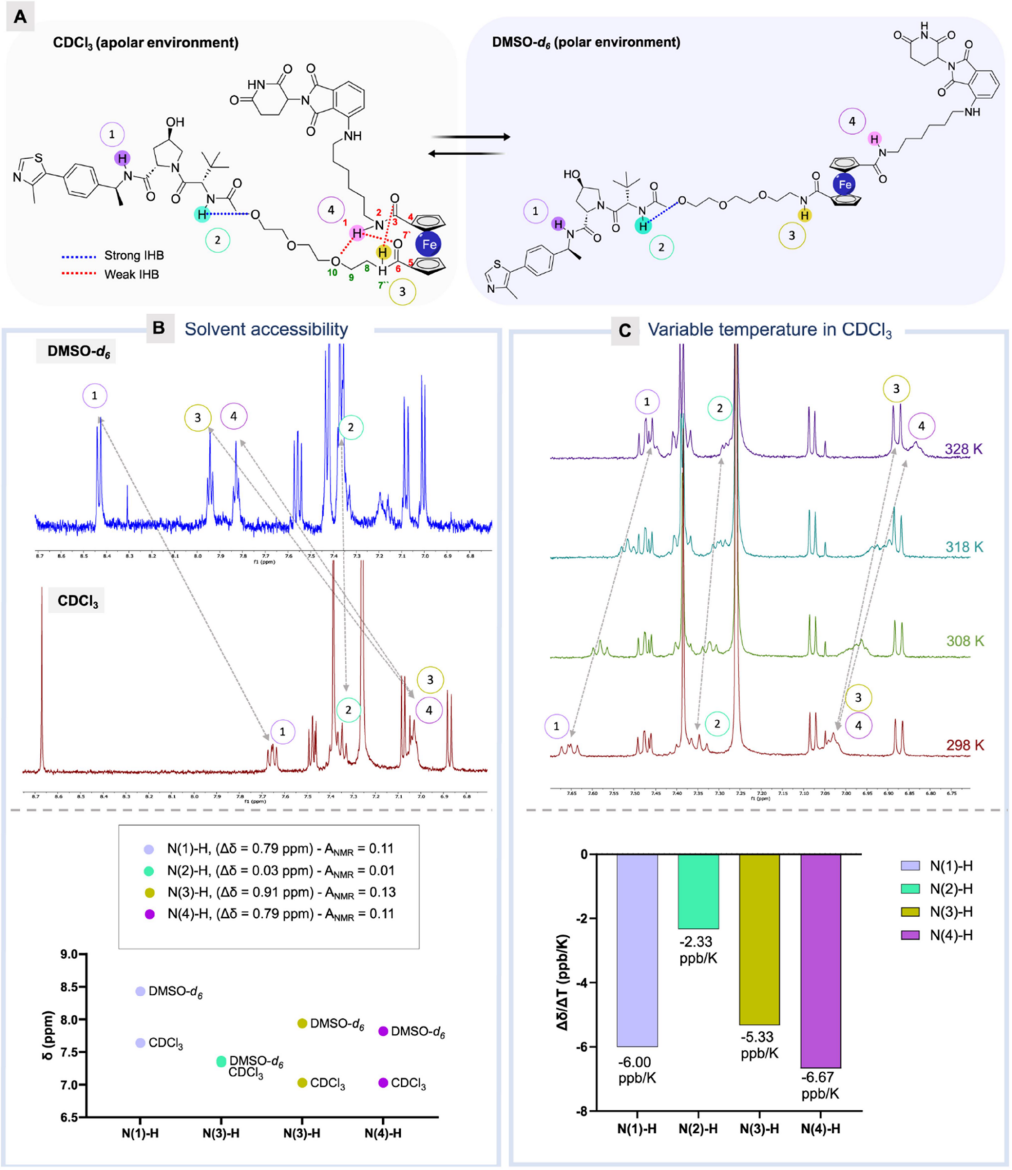
**Conformational studies on FerroTAC AS4 based on HBD shielding analysis.** (A) Conformational changes of FerroTAC **AS4** in nonpolar (CDCl_3_, left) and polar (DMSO-*d*_*6*_, right) environments, based on the assumed IMHB pattern. (B) NMR spectra of **AS4** recorded in CDCl_3_ and DMSO-*d*_*6*_ to evaluate solvent accessibility of the amide protons N(1–4)-H. A_NMR_> 0.15: no IMHB, 0.15 < *A*_NMR_ < 0.05: weak IMHB, A_NMR_< 0.05: strong IMHB. (C) Changes in chemical shifts (Δδ−tabulated in Table S1) observed in vt-NMR experiment of **AS4** in CDCl_3_ (*c* = 1.24 mM) from 298 to 328 K. Δδ/Δ*T* < 2.4 ± 0.5 ppb/K: strong IMHB or no IMHB, Δδ/Δ*T* > 2.4 ± 0.5 ppb/K: weak IMHB.

Considering the assumed flexibility of the ferrocene moiety, we anticipated the amide protons in **AS4** next to the ferrocene moiety, i.e., N(3)-H and N(4)-H, to show greater shielding effects in CDCl_3_ as compared to DMSO-*d*_*6*_, consistently with a preference for the *cis* conformation. In contrast, in the presence of a strong hydrogen-bond acceptor solvent such as DMSO-*d*_*6*_, the intercalation of polar solvent molecules is likely to disrupt the IMHB, favoring an expanded *trans* conformation^50^ of the substituent chains (Figure 4A). An initial analysis of the ^1^H NMR spectra was obtained on samples in deuterated CDCl_3_ and DMSO-*d*_*6*_. The gradual addition of DMSO-*d*_*6*_ typically leads to significant changes in the chemical shift of free or weakly hydrogen-bonded NH protons, while strong intramolecularly hydrogen-bonded NH groups tend to show little or no effect switching to DMSO-*d*_*6*_.^47^ As a result, NH protons with larger Δδ = (Δδ_DMSO_ - Δδ_CDCl3_) values indicate weak or absent IMHB, while smaller Δδ values suggest stronger IMHB. As shown **AS4** displayed overall smaller values of Δδ (Δδ_N(1)-H_ to Δδ_N(4)-H_ ≤ 0.91 ppm, Figure 4B *graph*), when comparing these amide protons in a chemical environment similar to the VHL ligand within its linear analogue **14a** (Δδ_N(1)-H_ = 1.16 ppm, Δδ_N(2)-H_ = 0.15 ppm, Figure S4). However, in both **14a** and **AS4,** N(2)-H appears to form stronger IMHB, consistent with crystallographic data^55^ and more recent NMR studies.^56^

The chemical shift difference between the two solvents (Δδ) can be converted into hydrogen bond acidity (A_NMR_) for a quantitative assessment of IMHB. Literature correlations provided threshold values associated with HBD participation in an IMHB and/or polarity shielding. An A_NMR_ ≥ 0.15 indicates the absence of IMHB, while values below 0.05 suggest strong IMHB. Values in the middle correlated with weak IMHB.^47^**AS4** exhibits overall A_NMR_ values for the NH around the threshold (≤0.13, Figure 4B, *graph*), indicating the presence of a flexible intermolecular hydrogen bond network likely mediated by the ferrocene moiety, which results in more shielded amides. Specifically, among the two amides adjacent to the ferrocene core, the N(4)-H appears to be more engaged in IMHB compared to N(3)-H, as indicated by slightly lower A_NMR_ values for N(4)-H (0.11 vs 0.13). This might be due to a hydrogen bonding pattern that resembles that found in heterochiral peptides through the formation of an antiparallel β-sheet-like structure. According to this speculation, both N(3)-H and N(4)-H seem to participate in a seven-membered ring (Figure 4A, in red),^57^ with N(4)-H accessing an additional conformation where the oxygen from the PEG group might serve as a hydrogen bond acceptor through a stabilized ten-membered ring (Figure 4A, in green). While highly indicative for conformational studies, the A_NMR_ values however reflect changes between solvents, which in turn indicate shifts in the solution conformational ensembles. For large, flexible molecules like PROTACs, the conformational ensembles can vary significantly between different solvents with distinct chemical properties and polarity. These differences may include variations in the number of selected conformers, their population distributions, and the specific conformations adopted. Consequently, A_NMR_ values can be affected by factors beyond IMHB, reflecting more extensive alterations in the molecule’s overall conformational landscape.

To gain an extra level of information, we investigated the stability of the intermolecular hydrogen-bonded structures by measuring the temperature dependence of the chemical shifts of the amide protons in the range of 298–328 K in CDCl_3_. The temperature coefficient (Δδ/ΔT) is primarily affected by alterations in conformational ensembles, which are mainly but not exclusively attributable to changes in hydrogen-bonding patterns. In the vt-NMR experiment performed in CDCl_3_, small temperature dependencies (Δδ/Δ*T* < 2.4 ± 0.5 ppb/K) can be observed for solvent-exposed NH protons as well as for those that are shielded from solvent and remain shielded over the temperature range of the measurements. Larger temperature dependencies are instead observed if the NH group is shielded from solvent initially but becomes exposed with increasing temperature, thus when intramolecularly hydrogen-bonded conformations unfold as the temperature increases.^48,49^ Variable temperature experiments in CDCl_3_ for **AS4** (Figure 4C, *graph* and full spectra at Figure S2B) showed Δδ/Δ*T* > - 2.4 ppb/K [N(4)-H = −6.67 ppb/K, N(1)-H = −6.00 ppb/K, N(3)-H = −5.33 ppb/K]. Such values might support the hypothesis of amide protons being initially shielded due to weak IMHB favored by a *cis* conformation. In detail, for the amides adjacent to ferrocene, N(4)-H seems to be more initially shielded compared to the counterpart N(3)-H in agreement with the intramolecular pattern of hydrogen bonds discussed above. The temperature coefficient suggests indeed that N(4)-H is more influenced by changes in the local environment compared to N(3)-H, which could be due to the disruption of an additional IMHB in a more stable and populated ensemble. It is reasonable to speculate that N(4)-H’s potential to form a secondary IMHB with PEG could account for its slightly greater tendency to engage in IMHBs and consequently, this disruption may have a larger effect on its temperature coefficient. In both **14a** (Figure S4) and **AS4**, the amide N(2)-H (Δδ/ΔT**_14a_** = −1.57 ppb/K and Δδ/ΔT**_AS4_** −2.33 ppb/K) appears to be involved in strong IMHB, as indicated by minimal chemical shift changes and aligned with the small A_NMR_ value. Interestingly, N(1)-H in the methylated analogue of VH032 of **AS4** appears to be initially shielded to a greater extent compared to **14a** (A_NMR**AS4**_ = 0.11 vs A_NMR**14a**_ = 0.16 and Δδ/ΔT**_AS4_** = −6.00 ppb/K vs Δδ/ΔT**_14a_** = −3.52 ppb/K) possibly due to interactions with the CRBN counterpart in a *cis* conformation or due to an enhanced shielding effect by the methyl group of MeVH032. Eventually, two-dimensional NOESY spectroscopy was used to gain better insight into the folding patterning of **AS4** using CDCl_3_ and the protic methanol-*d*_*4*_ that better resembles the hydrogen-bonding capabilities of water compared to DMSO-*d*_*6*_Table S2 summarizes the possible cross-ligand NOEs observed in both solvents as indicated in Figure S3A–L. The overlap of some peaks, however, hinders the definitive identification of the NOE origins, requiring careful interpretation. Nevertheless, a thorough analysis of the NOESY spectra indicated a higher likelihood of cross-ligand NOEs in CDCl_3_ than in methanol-*d*_*4*_. This suggests a potential *cis* configuration of the ferrocene in an apolar environment, but not in a polar one.

Taken together, the NMR experimental evidence suggests that **AS4** features a IMHB network of weak to moderate strength. These findings suggest furthermore that the FerroTACs can adopt more compact conformations in nonpolar solvents potentially acting as molecular chameleon. The conformational changes may occur through independent rotational adjustments of the ferrocene substituents around the central ferrocene core, as indicated by the shielding of amide signals in apolar solvents. Such conformational changes could lead to a reduced PSA and a smaller radius of gyration in the *cis* conformation, which would align with an enhanced permeability.

### Evaluation of FerroTACs-Mediated Protein Degradation

With the FerroTACs in hand, and the proposed conformational bias established, we proceeded to systematically evaluate their degradation profiles in cellular contexts. To determine the cellular potency of the VHL-targeting homo-FerroTACs, we conducted an initial screening by Western blot in HEK293 cells at two time points (4 and 24 h) and concentrations (0.1 μM and 1 μM), employing **CM11** and **CMP98** as positive and negative controls for VHL degradation, respectively. In the initial screening (Figure S5A), degradation of the pVHL30 isoform induced by FerroTAC **AS1** — a direct analogue of **CM11** — was notably less effective than that of AS2 and its parent analogue. Specifically, **AS1** achieved a maximal degradation (*D*_max_) of 48% for pVHL30 after 24 h at 1 μM, compared to the 100% degradation observed with **CM11** and **AS2** under identical conditions (Figure S5A).

**Figure 5:**
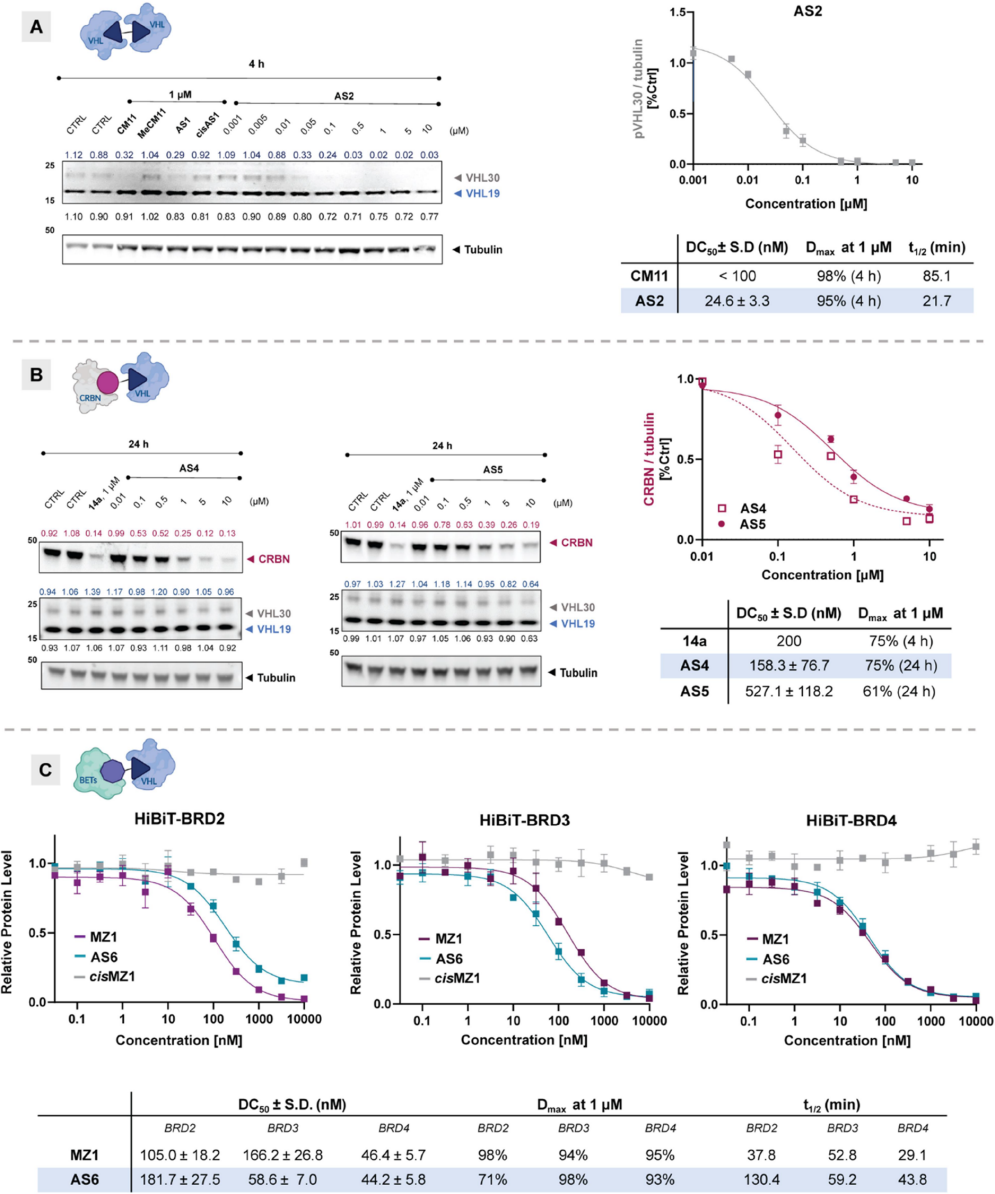
**Degradation profiles of FerroTACs AS2, AS4, AS5, and AS6.** (A) Representative blot of dose–response profile of HEK293 cells exposed to increasing concentrations of **AS2** for 4 h. Cells were treated with 0.5% ethanol with controls at 1 μM. The VHL/tubulin protein ratios were normalized to the average of the controls (100%). Data represent the mean values from two biologically independent experiments. (B) Representative blot of dose–response profile of HEK293 cells treated with increasing concentrations of **AS4** and **AS5** for 24 h. Cells were treated with 0.5% isopropanol, with controls at 1 μM. The VHL/tubulin protein ratios were normalized to the average of the controls (100%). Data represent the mean values from two biologically independent experiments. (C) Dose–response profile of HiBiT-tagged HEK293 cells treated with increasing concentration of **AS6** and **MZ1** in HiBiT-tagged BETs at 6 h. Data represent the mean values from two biologically independent experiments and four technical replicates.

Encouraged by the consistent and promising degradation results with **AS2**, we prioritised this compound for further detailed activity profiling. We subsequently assessed its concentration- and time-dependent activity in HEK293 cells (Figures 5A and S5B). Given that **AS2** incorporates a VHL ligand methylated at the benzylic position (MeVH032), we included its direct analogue **MeCM11** as benchmark in the concentration-dependent experiment (Figure 5A). However, **MeCM11** failed to induce significant degradation after 4 h at 1 μM, aligning with previous findings from our group^41^ and reinforcing that **AS2**’s superior activity is not simply due to incorporation of a more potent VHL binder. AS2 demonstrated robust pVHL30 degradation, with a half-maximal degradation concentration (DC_50_) of 24.6 nM (Figure 5A, *table*), achieving a *D*_max_ of 95% after 4 h at 1 μM. Notably, **AS2** also exhibited a significantly rapid degradation onset with half-life (*t*_1/2_) of 21.7 min at 1 μM, in contrast to **CM11**’s *t*_1/2_ of 85.1 min (calculated from^38^, Figure 5A, *table*). To further elucidate the mode of action of FerroTAC **AS2**, we explored its dependency on ubiquitination and proteasome activity. The degradation induced by **AS2** was effectively blocked by pre-treatment with MLN4924,^58^ MG132,^59^ and a competitive assay using VH032, confirming the anticipated E3-mediated ubiquitination and proteasome dependence (Figure S5C).

We next evaluated the cellular activities of CRBN/VHL-targeting FerroTACs. Using immunoblot analysis, we quantified VHL and CRBN protein levels in HEK293 cells following treatment with 0.1 μM and 1 μM of the compounds for 6 and 24 h, with **CM11**, **14a** and *cis***AS1**, serving as positive and negative controls, respectively (Figure S6A). Interestingly, significant CRBN degradation was observed with **AS4** and **AS5** at 1 μM, while no substantial VHL degradation was detected, consistent with the reported profile of **14a**. Consequently, **AS4** and **AS5** were selected for dose-dependent studies (Figure 5B), revealing DC_50_ values of approximately 158 nM for **AS4** and 527 nM for **AS5**, compared to **14a**’s DC_50_ of 200 nM^39^ (Figure 5B, *table*). For BET proteins, the degradation data from **AS6** and **AS7** in the initial screening via Western blot (Figure S6B) was confirmed by lytic and live-cell kinetic degradation assays in HEK293 cell lines expressing HiBiT-tagged BRD2, BRD3, and BRD4. **AS7** was found to be inactive in the initial screening at 6 and 24 h (Figure S6B) and was excluded from further evaluation. We investigated **AS6** in HiBiT-tagged cells with varying concentrations, alongside the positive control **MZ1** and the negative control *cis***MZ1**. **AS6** induced the degradation of BRD2, BRD3, and BRD4 with DC_50_ values of approximately 181, 59, and 44 nM, respectively, compared to **MZ1**, which showed DC_50_ values of 105 nM, 166 nM, and 46 nM (Figure 5C). Kinetic studies (Figure S7) further demonstrated rapid and complete degradation of these targets with comparable *t*_*1/2*_ to the reference compound (Figure 5C, *table*).

Together, our initial data demonstrate the degradation activity of FerroTACs in cellular context for all three considered model systems. The introduction of the ferrocene moiety did not negatively impact the PROTACs’ activity and in some cases, even enhanced their potency or selectivity. The retention of activity, especially when the modification is centrally located, suggests that ferrocene incorporation can be tolerated and favorable for PROTAC function.

### Evaluation of FerroTACs Cytotoxicity

**Figure 6:**
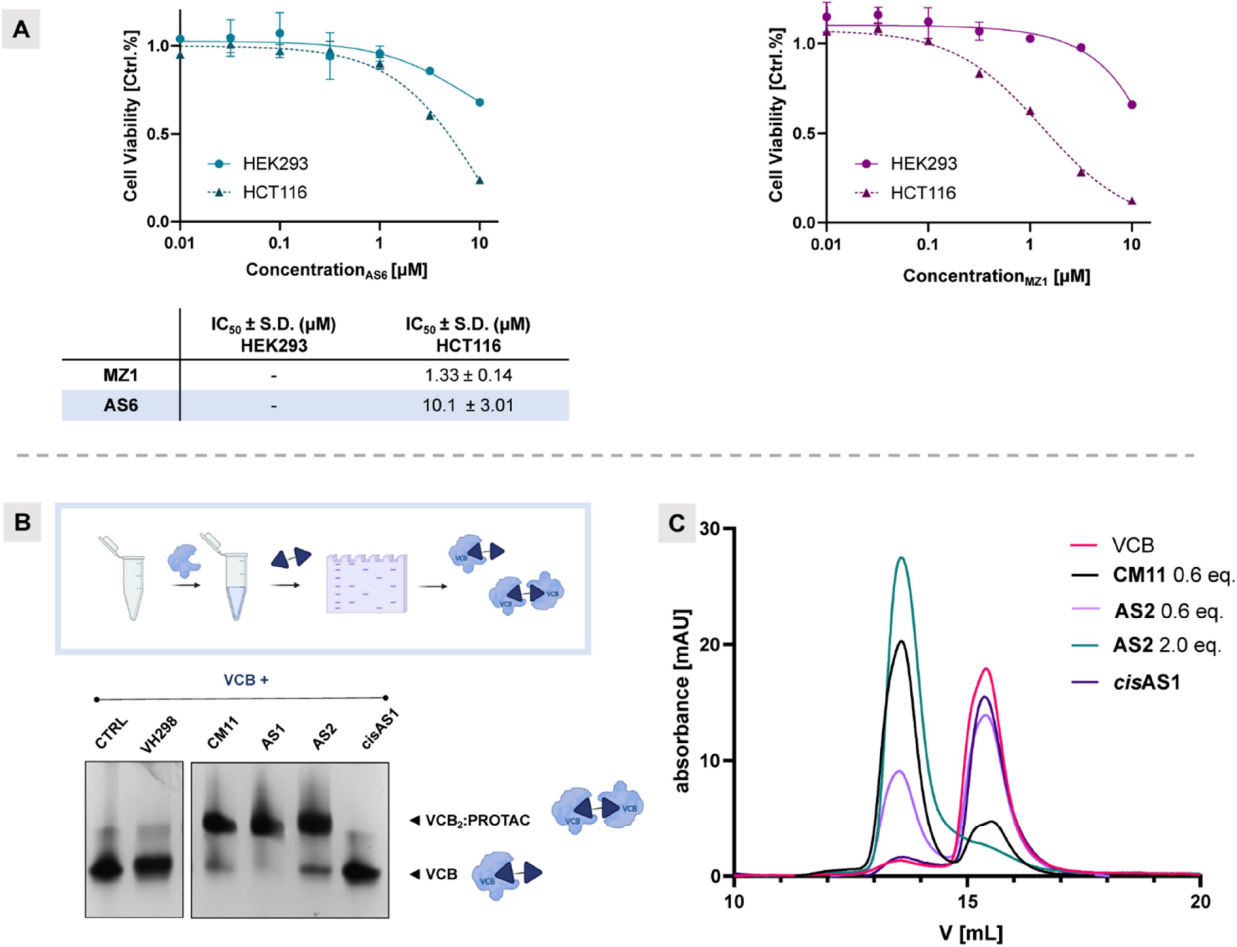
**Assessment of FerroTACs’ cytotoxicity and ability to induce ternary complexes.** (A) Cytotoxic effects on cellular proliferation were assessed with increasing concentrations of **AS6** (*left*) and **MZ1** (*right*) in HCT116 (dotted lines) and HEK293 (solid lines) after 72 h (mean ± S.D.; *n* = 2 biological replicates, four technical replicates). (B) Representative native gel for detection of ternary complex formation. VCB was preincubated with control **CM11**, **AS1**, **AS2**, *cis***AS1** or 5% EtOH for 30 min. (C) Size exclusion chromatography (SEC) UV chromatograms of complex formation after incubation of VCB with the vehicle (red), **CM11** (black), **AS2** (lilac), 2.0 equiv of **AS2** (green), and *cis***AS1** (purple).

Having confirmed FerroTACs̀ degradation activity, we investigated the potential for undesired intrinsic toxicity of ferrocene since this is commonly used to enhance cytotoxicity in antitumoral (i.e., ferrocifen^60^) and antiparasitic (i.e., ferroquine^35^) therapies due to its ability to mediate reactive oxygen species (ROS) production through the Fenton reaction.^32^ Therefore, we assessed the impact of the BET-degrading FerroTAC **AS6** on the viability of BET-sensitive HCT116 cells and HEK293 cells (Figure 6A). The best performing BET-degrading PROTAC **AS6** was chosen for cytotoxicity investigation due to the reliability of BET-sensitive cell lines (HCT116),^61^ which help differentiate between ferrocene unspecific cytotoxic effects from those arising from the on-target degradation. In HCT116, **AS6** exhibited a significant antiproliferative effect, with IC_50_ = 10.1 μM only 10-fold lower than **MZ1** (IC_50_ = 1.33 μM), and consistent with its less pronounced degradation profile. Importantly, **AS6**, similarly to **MZ1**, showed almost no intrinsic cytotoxicity in HEK293 cells (Figure 6A) highlighting the inherent tolerability and nontoxicity of ferrocene itself in this context. These findings further support that degradation is the primary mechanism driving cytotoxicity in BET-sensitive cell lines, while ferrocene is well tolerated in a cellular context up to high micromolar concentrations.

### FerroTAC-Mediated Ternary Complex Formation

To monitor ternary complex formation mediated by FerroTACs, we leveraged native gel electrophoresis assay, a methodology recently validated to assess VHL dimerization by homo-PROTACs.^41^ This in vitro approach serves as a rapid semiqualitative tool to evaluate homo-FerroTAC activity in promoting stable ternary protein complexes. The technique is user-friendly, suitable for higher throughput, and requires lower protein quantities compared to other biophysical methods. The recombinant VCB protein complex (VHL:ElonginC:ElonginB) was incubated with the corresponding Homo-PROTACs (**CM11**, **AS1**, *cis***AS1**, **AS2**) for 30 min at room temperature, followed by non-denaturing gel electrophoresis. Ternary complex formation was indicated by an upward shift in the protein band on the gel, in agreement with the higher molecular weight of the complex. Both **AS1** and **AS2**, similarly to reference compound **CM11**, induced formation of an up-shifted protein band, consistent with the formation of stable ternary complexes VCB_2_:PROTAC (Figure 6B). Competitive assays were conducted to unequivocally assign the migrating bands to the expected ternary complexes by preincubating VCB with increasing concentrations of the high-affinity inhibitor VH298 (Figure S8A) or PROTAC **AS2** (Figure S8B). For example, the concentration of VH298 was increased to displace PROTAC **AS2** from the ternary complex with VCB, promoting the formation of a preferred binary system, as indicated by the more prominent appearance of the lower band. Similarly, the formation of the **AS2**-mediated ternary complex after VH298 displacement was concentration-dependent, as evidenced by the increased intensity of the upper band (Figure S8).

To validate the ability of **AS2** to induce ternary complex formation, we turned to size exclusion chromatography (SEC) as an orthogonal method to the previously performed native gel electrophoresis assay.^38^ Using **CM11** as benchmark, we confirmed the formation of a VCB_2_:**AS2** ternary complex, which migrated quicker than the VCB vehicle control (Figure 6C). Various conditions were tested, both with and without an excess of **AS2** (green and lilac lines, Figure 6C), resulting in the complete or partial formation of the VCB_2_:**AS2** ternary complex, respectively. This was confirmed by the retention time comparison observed for the **CM11**-mediated ternary complex. As controls, *cis***AS1** did not form any ternary complex, leading to identical retention time as VCB in vehicle as control. The formation of the ternary complex with **AS2** was observed to be slightly less pronounced compared to **CM11**, a highly cooperative dimerizer.^38^ Despite this, **AS2** proved to be a potent and full degrader of pVHL30, much like **CM11** (Figure 5A), consistent with potential increased cell permeability of **AS2** compared to **CM11** which is known have very low cell permeability.^55^ Together the results from both native gels and SEC indicate that **AS2** works by effectively promoting the formation of a ternary complex, further supporting the general PROTAC-like behavior of FerroTAC compounds.

### Evaluation of Cellular Permeability

The data so far establish that FerroTACs act as degraders through ternary complex formation, with ferrocene exhibiting high cellular tolerance. We next turned our attention to the hypothesis that the rotational flexibility of ferrocene incorporated into FerroTACs—initially evaluated by NMR studies—could increase the chameleonic behavior, thus allowing for improved cellular permeability. To this end, we assessed the PROTAC cellular permeability by evaluating VHL target engagement using the Promega NanoBRET TE Intracellular E3 Ligase.^62^ Current PROTAC permeability assays use artificial systems like PAMPA and Caco-2, designed for small molecules but not always effective for PROTACs due to their low permeability, high molecular weight and assay caveats (i.e., unspecific binding and low recovery).^63,64^ The NanoBRET assay is a high-throughput method validated for measuring E3 ligase engagement in live and permeabilised cells. The assay allows to rank PROTAC intracellular availability through competitive probe displacement and offers a simple workflow for prioritising compounds based on properties associated with permeability. Additionally, the NanoBRET intracellular availability scores have been shown to align with permeability rankings from other methods.^62^ Specifically, in the live-cell mode, the assay measures the apparent cellular affinity, where the plasma membrane is intact and can impede the compound’s access to its target. In the permeabilised-cell mode, the barrier posed by the plasma membrane is removed and the intrinsic affinity of the compound for target is measured. Compounds with lower intracellular availability show a greater right-shift in potency in live-cell mode compared to permeabilised-cell mode. In contrast, highly permeable compounds exhibit similar potency across both conditions. The ratio of potencies between live-cell and permeabilised-cell modes, referred to as the relative-binding affinity (RBA) value increases as intracellular availability decreases and is thus a metric for assessing a PROTAC̀s cellular availability. To facilitate this assessment, the permeable control compound **VH298** was included to standardize the RBA value, generating an availability index (AI) for each PROTAC.^62^ The best-performing degraders for each system (**AS2**, **AS4**, and **AS6**) were selected and studied for VHL target engagement in comparison to the reference Fc-free PROTACs (**CM11**, **14a**, and **MZ1**, respectively).

**Figure 7:**
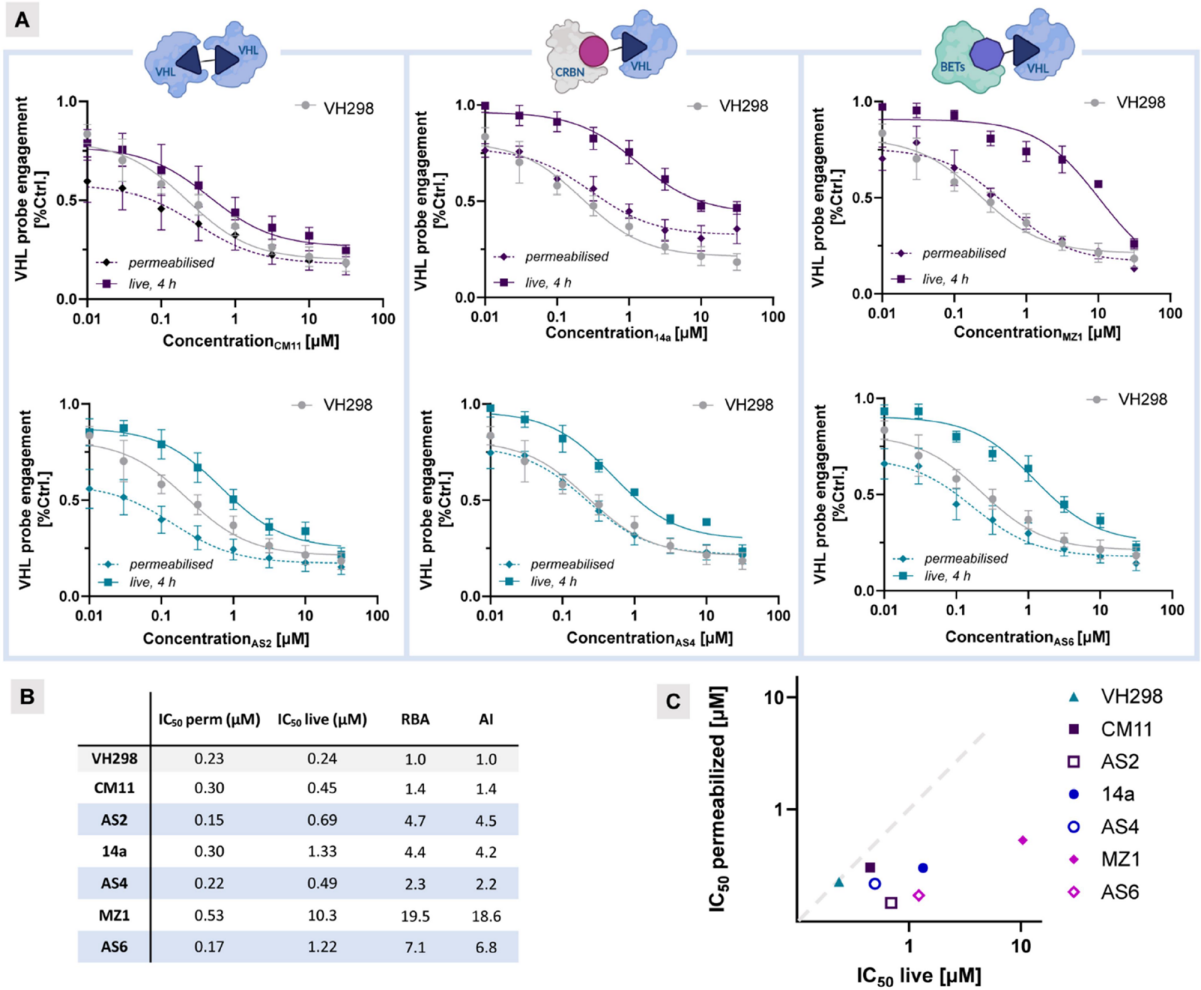
**NanoBRET E3 Ligase target engagement assays were performed for the tested compounds against VHL in both permeabilised and live-cell modes.** (A) HEK293 cells transfected with VHL-NanoLuc were treated with titrated concentrations of the indicated compounds (reference compounds **CM11**, **14a**, **MZ1**, *top*; FerroTACs, **AS2**, **AS4**, **AS6***bottom*) in the presence of VHL tracers (0.5 μM for permeabilised cells and 1 μM for live-cell assays). The fractional occupancy of the tracers was plotted against the concentrations of the tested compounds and fitted accordingly. Data points represent the mean ± SEM from three independent experiments. (B) Tabulated data of IC_50_ (live and permeabilised), relative binding affinity (RBA, calculated as RBA = IC_50*live*_÷ IC_50*perm*_), and availability index (AI, calculated as AI = RBA_PROTAC_ ÷ RBA_VH298_). (C) Graphical representation of the IC_50_ live/permeabilised ratio for FerroTACs (empty symbols) and reference compounds (solid symbols), with the diagonal line indicating AI = 1.

In the homo-VHL system, the method’s limitation in distinguishing between binary and ternary binding may mask potency differences, diminishing the contrast between **AS2** and **CM11** (Figure 7A). Consequently, it becomes challenging to differentiate the potency of **CM11** and **AS2**, although both compounds display a similar trend in VHL engagement. In contrast, within the CRBN/VHL system, compounds **14a** and **AS4** demonstrated 4-fold and 2-fold lower potency, respectively, in live-cell assays (IC_50*live*_ = 1.33 μM and 0.49 μM, Figure 7B) compared to permeabilised-cell assays (IC_50*perm*_ = 0.30 μM and 0.22 μM). This suggests that limited cellular permeability restricts VHL target engagement for both compounds. Notably, the smaller disparity in binding affinity between live-cell and permeabilised-cell conditions for **AS4** indicates that this compound possesses superior cell permeability relative to **14a**, as reflected by an AI that is 2-fold lower. Similarly, when tested for VHL engagement, both **MZ1** and **AS6** showed full engagement in cell-permeabilised mode (IC_50*perm*_ = 0.53 and 0.17 μM, respectively). However, the most significant difference was observed in live-cell mode, where **AS6** exhibited an IC_50_ only 10-fold lower than the permeabilised mode (IC_50*live*_ = 1.22 μM), in contrast to the larger IC_50_ difference observed with **MZ1** (20-fold, IC_50*live*_= 10.3 μM). Overall, FerroTACs demonstrated a smaller difference in VHL engagement between permeabilised and live-cell modes, suggesting higher intracellular availability, which may be linked to an improved cellular permeability. This ratio is depicted in Figure 7C, where the AI values of FerroTACs are positioned closer to the diagonal corresponding to AI = 1, indicating maximal permeability. However, exceptions such as **CM11/AS2** may highlight an inherent limitation of the assay.

### Investigations on the Role of Efflux Transporters in PROTAC Potency

**Figure 8:**
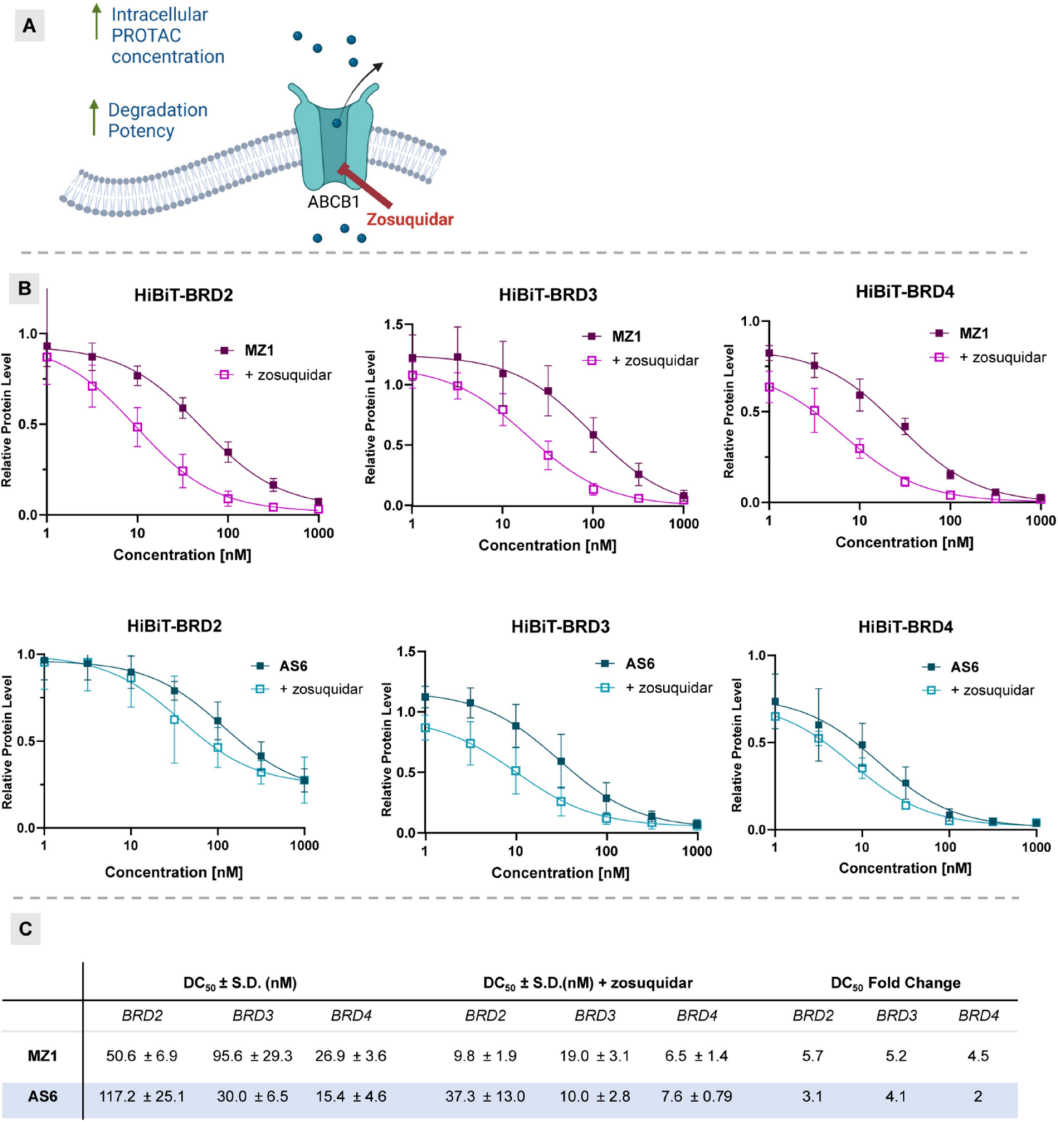
**Comparison of DC_50_values in HiBIT degradation assay with and without zosuquidar shows that FerroTACs are less susceptible to efflux mechanisms.** (A) Graphical representation of zosuquidar mechanism of action; (B) Dose–response curves of the tested compounds (**MZ1**, *top*; **AS6**, *bottom*) with (empty squares) and without (solid squares) zosuquidar (500 nM) pretreatment. The left-shift in DC_50_ values indicates the influence of ABCB1/MDR1 inhibition by zosuquidar on the cellular potency of the compounds. Mean ± S.D., *n* = 3 biological replicates, six technical replicates. (C) Tabulated data of DC_50_ from **AS6** and **MZ1** with and without zosuquidar pretreatment, alongside the corresponding fold change in DC_50_.

Multidrug resistance (MDR) poses a significant challenge in anticancer treatments like chemotherapy, kinase inhibitors- and degrader-based therapies. In light of this, we aimed to explore the role of efflux transporters on the degradation efficiency of FerroTAC degraders. A well-studied mechanism of MDR is indeed the enhanced export of drugs by ATP-binding cassette (ABC) efflux transporters, such as ABCB1/MDR1 (encoding for P-glycoprotein, P-gp), ABCC1/MRP1, and ABCG2/BCRP.^65^ Recent evidence indicates that PROTACs might be substrates for the ABCB1/MDR1^66,67^ and ABCC1/MRP1^68^ transporters, suggesting that efflux could limit compounds intracellular availability and reduce their efficacy (Figure 8A). Unlike other transporters, ABC ones are generally less restricted by substrate size and properties and recognize structurally and chemically unrelated hydrophobic and/or weakly amphipathic compounds.^69^

To investigate the effect of P-gp efflux on FerroTACs, we conducted a HiBiT degradation assay with and without subtoxic concentrations of the ABCB1/MDR1 inhibitor, zosuquidar.^70^ The HiBiT degradation assay is a sensitive technique that monitors protein degradation by detecting the luminescence emitted by the HiBiT tag on the target protein bound to the NanoLuc luciferase. In this study, we used this assay to assess the impact of blocking P-gp transporters on the intracellular availability and subsequent degradation potency of PROTAC **MZ1** and its corresponding FerroTAC, **AS6**. By doing so, we aimed to determine variations in degradation potency (DC_50_) as consequence to the efflux transporter inhibition, and thus providing insights into how P-gp transporters may influence the degradation efficacy of both Fc-free and FerroTAC compounds.

Our results showed that 1-h pre-treatment with zosuquidar significantly enhanced the potency of both **MZ1** and **AS6** (Figure 8B,C). Specifically, **AS6** exhibited 2- to 4.1-fold increase in DC_50_ for BRD2 and BRD3/4, while **MZ1** showed up to a 5.7-fold increased DC_50_ for BRD2, suggesting that this class of efflux transporters have a role in limiting the intracellular availability of **MZ1** to a greater extend compared to **AS6**.

It is well-established that the compound’s HBD count and, consequently, its PSA play a crucial role in determining P-gp efflux, with an HBD of less than 2 being necessary to maximize the likelihood of avoiding P-gp efflux.^71^ Formation of IMHB through structural folding mediated by the ferrocene moiety, could reduce the apparent HBD count, thereby increasing the likelihood of FerroTACs to evade efflux mechanisms compared to Fc-free analogues. Together our collective data suggests that by promoting more compact conformations, FerroTACs could benefit from enhanced passive diffusion or active uptake, as well as reduced susceptibility to P-gp efflux mechanisms.

### In Vitro Physicochemical Properties of FerroTACs

Following the characterization of FerroTACs in terms of conformational studies, degradation, ternary complex formation, permeability, and efflux ratio (as summarized in the Table 1A), we proceed with the evaluation of selected physicochemical properties and stability profiles (Table 1B) of the best-performing FerroTACs **AS2**, **AS4** and **AS6** in comparison with Fc-free references across each system.

**Table 1:** Experimental Overview and In Vitro Physicochemical Properties of FerroTACs^a^

system	VHL-VHL HomoPROTAC				CRBN-VHL				BETs-VHL		
compound	CM11	AS2	AS1	*cis*AS1	14a	AS3	AS4	AS5	MZ1	AS6	AS7
**(A) Overview of the Experimental Data**											
**conformational study (IMHB by NMR**)	–	–	–	–	•	–	•	–	*–*	*–*	–
**degradation**											
DC_50_	•	•	–	–	•	–	•	•	•	•	–
*D*_max_	•	•	•	–	•	–	•	•	•	•	–
*T*_1/2_ (min)	•	•	–	–	–	–	–	–	•	•	–
**ternary complex**	•	•	•	•	–	–	–	–	–	–	–
**cytotoxicity**	–	–	–	–	–	–	–	–	•	•	–
**permeability**	•	•	–	–	•	–	•	–	•	•	–
**efflux investigation**	–	–	–	–	–	–	–	–	•	•	–
**(B) In vitro Pharmacokinetic Parameters**											
**ChromLogD**	3.2	3.8	3.4	3.5	3.7	3.2	3.9	3.4	4.1	4.8	4.9
**LogD**	2.9	4.3	–	–	3.3	–	4.0	–	3.8	4.3	–
**kinetic solubility (μM)**											
PBS (pH 7.4)	58.2	6.4	–	–	8.7	–	0.34	–	22.35	2.52	–
FeSSIF (pH 5.8)	38.6	41.0	–	–	59.5	–	2.4	–	51.10	63.50	–
**stability (min)**											
*T*_1/2_ in mouse plasma	234.2	285.5	–	–	33.4	–	64.4	–	347.05	24.07	–
*T*_1/2_ in mouse microsome	25.8	31.3	–	–	1.1	–	2.3	–	2.50	21.40	–
Cl_int_ (mL/min/kg)	211.5	174.1			5089.1		2400.7		2183.6	255.06	

a • performed, – not performed.

The modulation of lipophilicity could play a critical role in optimizing PROTACs. We thus utilized traditionally measured LogD values from shake flask experiments alongside chromatographically assessed ChromLogD values.^72^ Despite minor differences between the two methods, both indicate that the incorporation of ferrocene significantly enhances the overall lipophilicity of the FerroTACs (LogD_7.4_**CM11** = 2.9 vs **AS2** = 4.3; **14a** = 3.3 vs **AS4** = 4.0, **MZ1** = 3.8 vs **AS6** = 4.3). This trend aligns with previous findings on ferrocene incorporation^36^ and suggests that this approach could be valuable in optimizing the pharmacokinetic profiles of PROTACs and other bifunctional molecules containing highly polar ligands as well as to direct specific compartment accumulation. By incorporating ferrocene, permeability may be improved due to the feature of promoting IMHB-mediated compact conformations, while also balancing the lipophilicity of the polar ligand/PEG linkers. However, while sufficient lipophilicity is required for passive uptake over the cell membrane, it may also drive poor aqueous solubility.^73^ In fact, FerroTACs exhibited a 10-fold lower solubility (0.34–6.4 μM) in phosphate-buffered saline (PBS, pH 7.4) compared to the Fc-free reference compounds (8.7–58.2 μM). However, the FerroTACs biorelevant solubility in Fed State Simulated Intestinal Fluid (FeSSIF) at pH 5.8, was found to be higher than that in buffered aqueous solution in two out three systems, potentially reducing the extent of solubility-limited absorption in vivo for FerroTACs.

The complex metabolism of PROTACs can potentially influence pharmacokinetics, therefore plasma and microsomal stability were assessed. The plasma stability of the FerroTACs showed varying trends depending on the degrader system (*T*_1/2_ in mouse plasma, reference vs FerroTAC: VHL-VHL 234.2 min vs 285.5 min, CRBN-VHL 33.4 min vs 64.4 min, and BETs-VHL 347.05 min vs 24.07 min). In contrast, microsomal stability for FerroTACs was consistently 2–10 times higher, with corresponding reductions in metabolic clearance (CL*_int_*). As expected, thalidomide-based degraders (**14a** and **AS4**), which are known to undergo spontaneous hydrolysis in aqueous solutions, exhibited similar low stability trends under both plasma and microsomal settings.

Overall, the data highlights the robustness of the FerroTACs in terms of lipophilicity, solubility, and metabolic stability, strengthening their potential for further development.

## Conclusions

In this proof-of-concept study, we have explored incorporating an organometallic moiety in PROTAC linkers to act as a molecular hinge and enabling dynamic conformational changes. By introducing ferrocene into homo- and hetero-PROTAC systems (FerroTACs), we demonstrate its potential to enhance protein degradation by facilitating the rational design of molecular chameleons and improving cellular uptake. The study of molecular chameleons is gaining attention in the field, although a standardized high-throughput method for quantifying chameleonicity descriptors in early drug discovery is still lacking, despite recent advancements in chromatography.^42,43^ We herein opted to perform conformational analyses using NMR spectroscopy to assess FerroTACs conformational adjustment in solution. Our findings indicate that in those structures, amide protons are more shielded in apolar solvent engaging in IMHB and other intramolecular interactions. This supports our hypothesis that the flexible rotation of ferrocene might enable dynamic conformational changes and foster more compact conformations that likely enhance cellular permeability. Cellular studies show that FerroTACs are well-tolerated in cellular contexts and effectively induce target degradation *via* a PROTAC-like mechanism, with degradation activity comparable or enhanced relative to reference analogues. The NanoBRET VHL-engagement assay further validated the improved cellular permeability, and the compounds’ lower dependency on efflux mechanisms was shown. In vitro physicochemical properties evaluation revealed that incorporating ferrocene into bifunctional designs could also enhance permeability by effectively increasing LogD and lipophilicity. We foresee in this a strategy that holds significant potential for optimizing the pharmacokinetic profiles of PROTACs and other bifunctional molecules bearing highly polar ligands, where this can hinder compound cellular uptake and target engagement. Notably, the addition of ferrocene did not compromise solubility or metabolic stability that remained unchanged or in some cases, enhanced. This further underscores the versatility of this unconventional chemotype for modulating PROTAC drug-like characteristics.

Overall, our findings demonstrate the feasibility of incorporating ferrocene as a versatile linker moiety in bifunctional molecules, with potential applications extending beyond PROTACs to a broader range of induced-proximity strategies and therapeutic modalities,^74^ opening avenues for future exploration of novel bifunctional designs. Future work will also explore the potential of this strategy for difficult-to-degrade targets, such as those with low PROTACtability,^75^ and investigate its applicability to additional E3 ligase–target combinations, to expand the scope of bifunctional degraders. Additionally, significant emphasis will arise on expanding linker chemical space by incorporating alternative metal-based systems and advanced designs that might allow control of the *cis–trans* conformational equilibrium, unlocking future applications in chemical biology and bioconjugation.

## Supporting Information Available

Additional synthesis schemes; signal assignment and NOESY spectra for **AS4**; vt-NMR spectra for **14a**; representative immunoblots from initial degradation assay screening of **AS1**-**AS7**; representative immunoblots of time-course and mechanistic treatment for **AS1**; BET-HiBiT kinetic degradation for **AS6**; additional native gel data; and general experimental details and compound characterization (PDF)
