# Supplementary material for: Rational Design of PROTAC Linkers Featuring Ferrocene as a Molecular Hinge to Enable Dynamic Conformational Changes
Source: J Am Chem Soc. 2025 Apr 10;147(16):13328–44. doi: 10.1021/jacs.4c18354 (PMC12022980; doi:10.1021/jacs.4c18354)
Supplement: Supplementary file 1 — ja4c18354_si_001.pdf [file ja4c18354_si_001.pdf]

SUPPORTING INFORMATION

Rational Design of PROTAC Linkers Featuring Ferrocene as a Molecular Hinge to Enable Dynamic Conformational Changes

Alessandra Salerno, Lianne H. E. Wieske, Claudia J. Diehl, Alessio Ciulli*

Centre for Targeted Protein Degradation, School of Life Sciences, University of Dundee, 1 James Lindsay Place, Dundee DD1 5JJ, Scotland, United Kingdom

***Email: a.ciulli@dundee.ac.uk**

Table of Contents

Synthetic Schemes ____ 3

Scheme S1. 3

Conformational Analysis by NMR ____ 3

Figure S1. 3

Table S1. 4

Figure S2. 6

Table S2. 9

Figure S3A-L. 9

Biological Assays ____ 22

Figure S5. 22

Figure S6 23

Figure S7. 23

Figure S8. 24

General Experimental Details ____ 25

Chemistry 25

NMR studies. 31

Biology. 31

HPLC/HRMS Traces for compounds AS1-AS7 ____ 36

References ____ 44

Synthetic Schemes

Scheme S1.

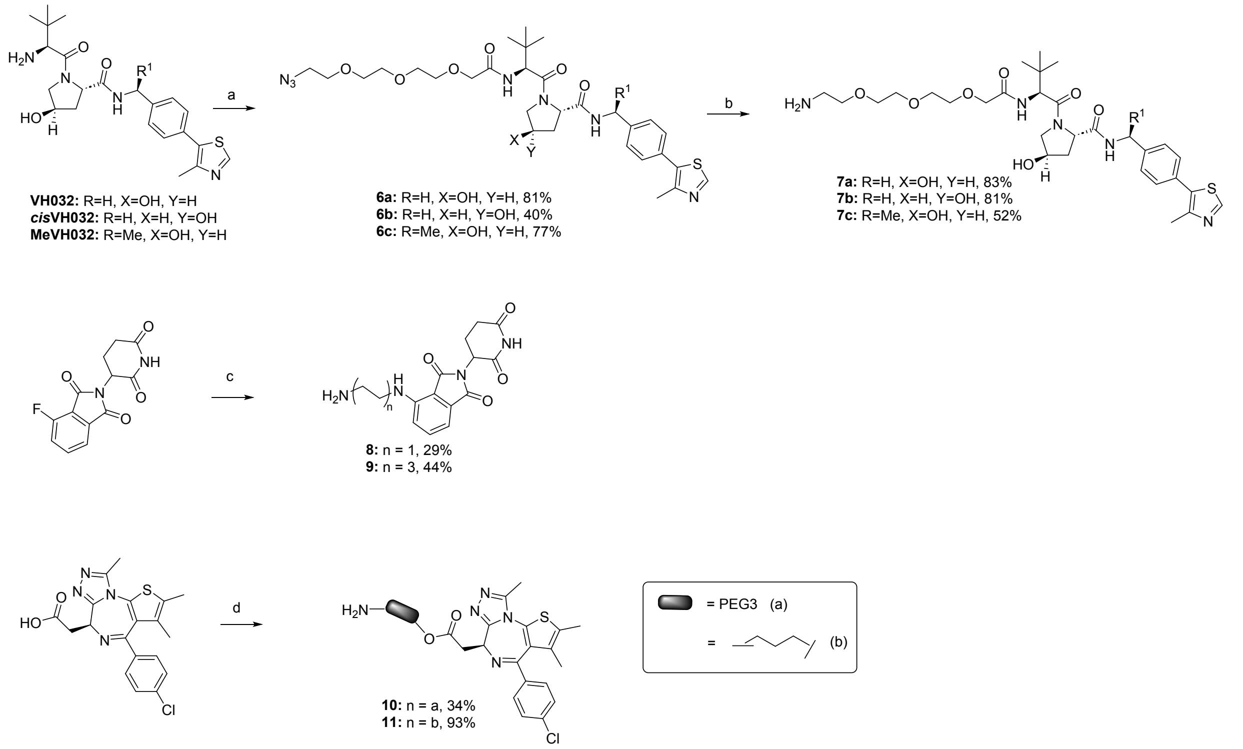

[a]**Reagents and conditions.** a) N_3-PEG3-COOH, HATU, DIPEA, dry DCM, r.t., overnight (40-81%); b) 1 atm H_2 on Pd/C10wt%, r.t., 3 h (52-83%); c) i) dry DMSO, DIPEA, 110 °C, overnight (29-44%); ii) Trifluoroacetic acid, dry DCM/MeOH, r.t., 2 h; d) i) $SOCl_2$, TEA, dry DCM, 0 °C to r.t.; 3 h; ii) *N*-Boc Amino-PEG3-alcohol or *tert*-butyl (3-hydroxypropyl)carbamate, dry DCM, r.t., overnight; iii) hydrogen chloride 4.0 M in 1,4-dioxane, r.t., 2 h (34-93%).

Conformational Analysis by NMR

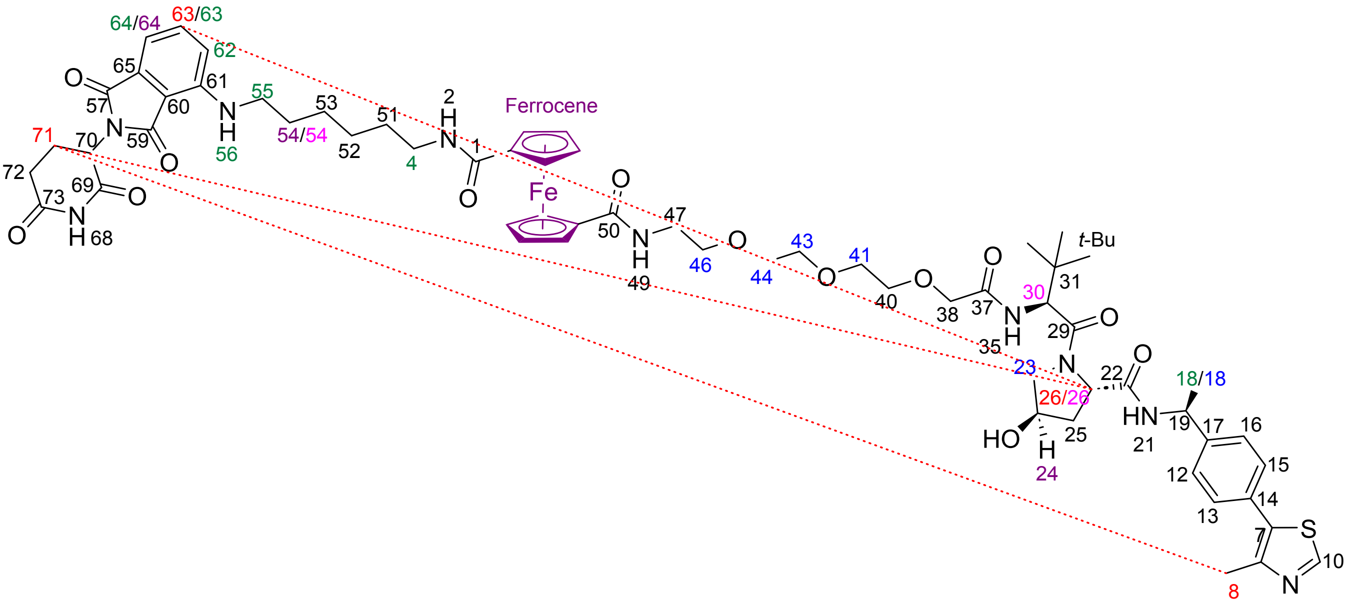

Figure S1.
Structure of **AS4** with numbering used for the conformational analysis. The main NOE cross-peaks from non-overlapped signals and detected across the VHL and CRBN ligand (shown in Figures S3A-L) are highlighted in red. *In green,* cross peaks originated from 18 (overlapping with the alkyl chain), *in blue* those from 23 (overlapping with PEG), *in purple* from ferrocene (overlapping with 24) and *in pink* those from 54 (overlapping with alkyl chain). All the cross peaks are tabulated in Table S2.

Table S1.

^{1}H and ^{13}C signal assignment for **AS4** in DMSO-d_6, $CDCl_3$ and methanol-d_4. In brackets signal assignment of amide protons at 328 K. Due to the large extent of signal overlap not all signals could be assigned unambiguously

Atom	DMSO-d_6 ^{1}H (ppm)	$CDCl_3$ ^{13}C (ppm)	$CDCl_3$ ^{1}H (ppm)	Methanol-d_4 ^{13}C (ppm)	Methanol-d_4 ^{1}H (ppm)
**VHL**					
10	-	150.4	8.67	152.9	8.87
8	-	148.6	-	149.1	-
8-Me	-	16.3	2.52	15.8	2.47
7	-	131.8	-	133.4	-
14	-	130.9	-	131.4	-
13-15	-	126.6	7.38	130.6	7.43
12-16	-	129.6	7.38	127.7	7.43
17	-	143.6	-	145.6	-
19	-	48.9	5.11	50.2	5.00
19-Me	-	22.5	1.48	22.5	1.50
21-NH (also **N(1)-H**)	8.43	-	7.64 (7.46)	-	n.a.[a]
22	-	170.39	-	173.2	-
26	-	58.8	4.75	60.6	4.60
25	-	36.4	2.41; 2.17	38.9	2.22; 1.96
24	-	70.7	4.53	71.0	4.44
24-OH	-	-	4.53 (bs)	-	n.a.[a]
23	-	57.2	4.07; 3.66	58.2	3.85; 3.75
29	-	n.a.[a]	-	171.8	-
30	-	56.9	4.65	58.1	4.69
31	-	35.7	-	37.1	-
t-Bu	-	26.6	1.04	27.0	1.04
35-NH (also **N(2)-H**)	7.37	-	7.34 (7.27)	-	n.a.[a]
**VHL linker**					
37	-	n.a.[a]	-	172.0	-
38	-	71.6-70.0	3.80-3.58	71.1	4.02; 3.94
40	-	71.6-70.0	3.80-3.58	72.2	3.72-3.63
41	-	71.6-70.0	3.80-3.58	71.7	3.72-3.63
43	-	71.6-70.0	3.80-3.58	71.7	3.72-3.63
44	-	71.6-70.0	3.80-3.58	71.7	3.72-3.63
46	-	71.6-70.0	3.80-3.58	71.7	3.72-3.63
47	-	71.6-70.0	3.80-3.58	70.7	3.69; 3.53
49-NH (also **N(4)-H**)	7.82	-	7.03 (6.83)	-	n.a.[a]
50	-	n.a.[a]	-	172.6	-
**Ferrocene**					
CH	-	71.2; 70.6; 70.5	4.59; 4.53; 4.51; 4.36; 4.33; 4.32	73.6; 73.5; 73.4; 71.3	4.76; 4.73; 4.41; 4.37
C	-	78.5; 77.9	-	78.8; 78.4	-
**CRBN**					
68-NH	-	-	n.a.[a]	-	n.a.[a]
69	-	168.9	-	174.37	-
70	-	49.1	4.91	50.2	5.04
71	-	23.0	2.76; 2.11	23.8	2.70; 2.09
72	-	31.6	2.85; 2.72	32.2	2.84; 2.75
73	-	168.9	-	174.8	-
59	-	169.7	-	170.9	-
57	-	167.8	-	169.3	-
60	-	110.1	-	148.3/133.9/111.2[b]	-
65	-	132.6	-	148.3/133.9/111.2[b]	-
61	-	116.9	-	148.3/133.9/111.2[b]	-
64	-	111.6	7.07	118.0	7.03

62	-	116.9	6.87	137.3	7.52
63	-	136.3	7.47	111.7	7.01
56-NH	-	-	6.23	-	
**CRBN linker**					
55	-	42.7	3.27	43.3	3.31
54	-	29.1	1.66	30.7	1.64
53	-	29.9/29.2/26.8[b]	1.71-1.41	27.8	1.51
52	-	29.9/29.2/26.8[b]	1.71-1.41	27.8	1.51
51	-	29.9/29.2/26.8[b]	1.71-1.41	30.3	1.71
4	-	39.9	3.55; 3.35	40.7	3.53; 3.34
2-NH (also **N(3)-H**)	7.94	-	7.03 (6.87)	-	n.a.[a]
1	-		-	172.3	-

[a]n.a. not assigned signals, these signals could not be assigned unambiguously.

[b]One of the indicated signals corresponds to the assigned atom, which one exactly could not be determined.

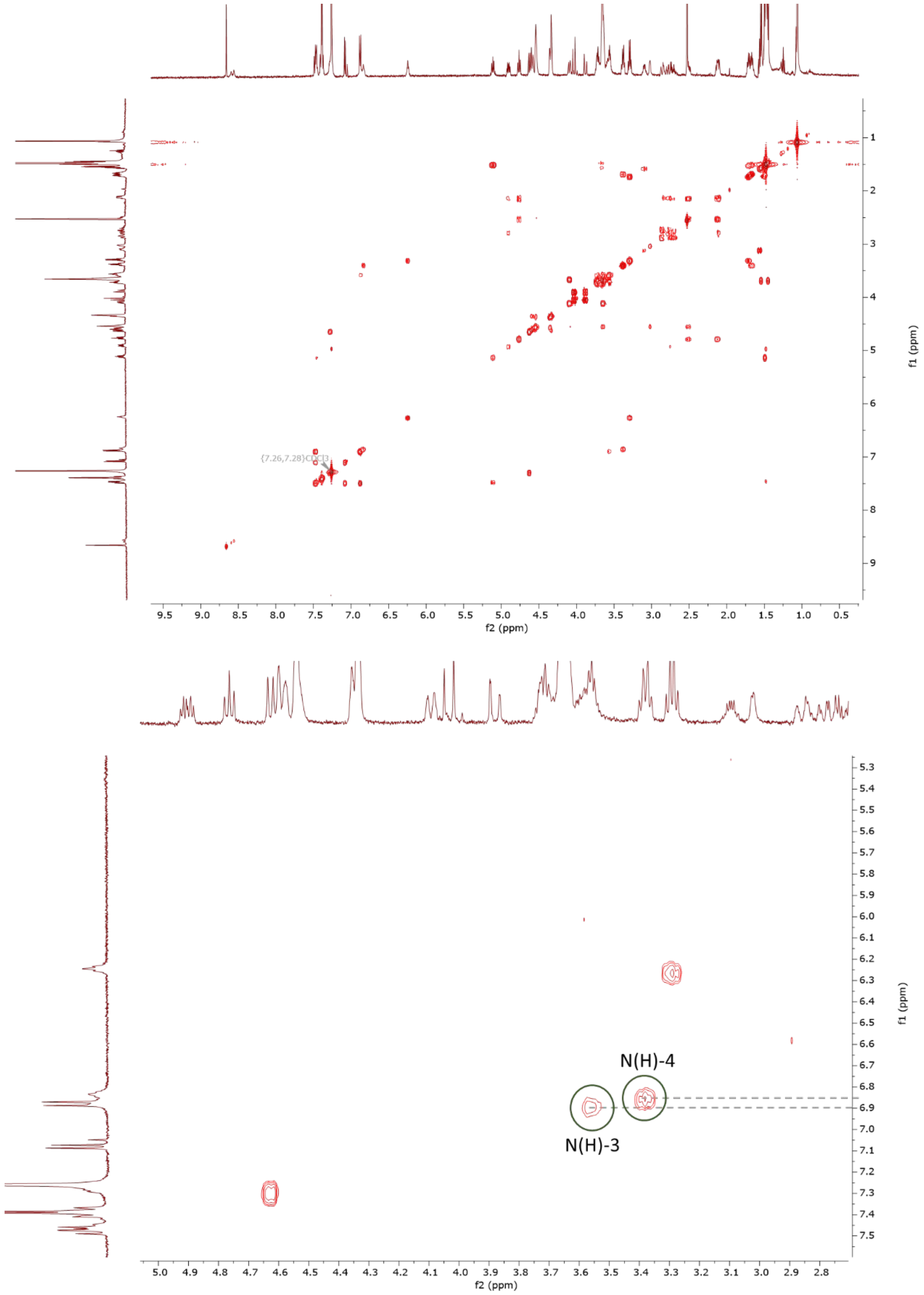

Figure S2A.
COSY spectra of **AS4** registered at 328 K. Full spectra *(top)* and zoom in (*bottom*) for the signal assignment of amides N(3)-H and N(4)-H that overlapped with other signals in the ^{1}H-NMR. Chemical shifts in ppm are reported in brackets in Table S1.

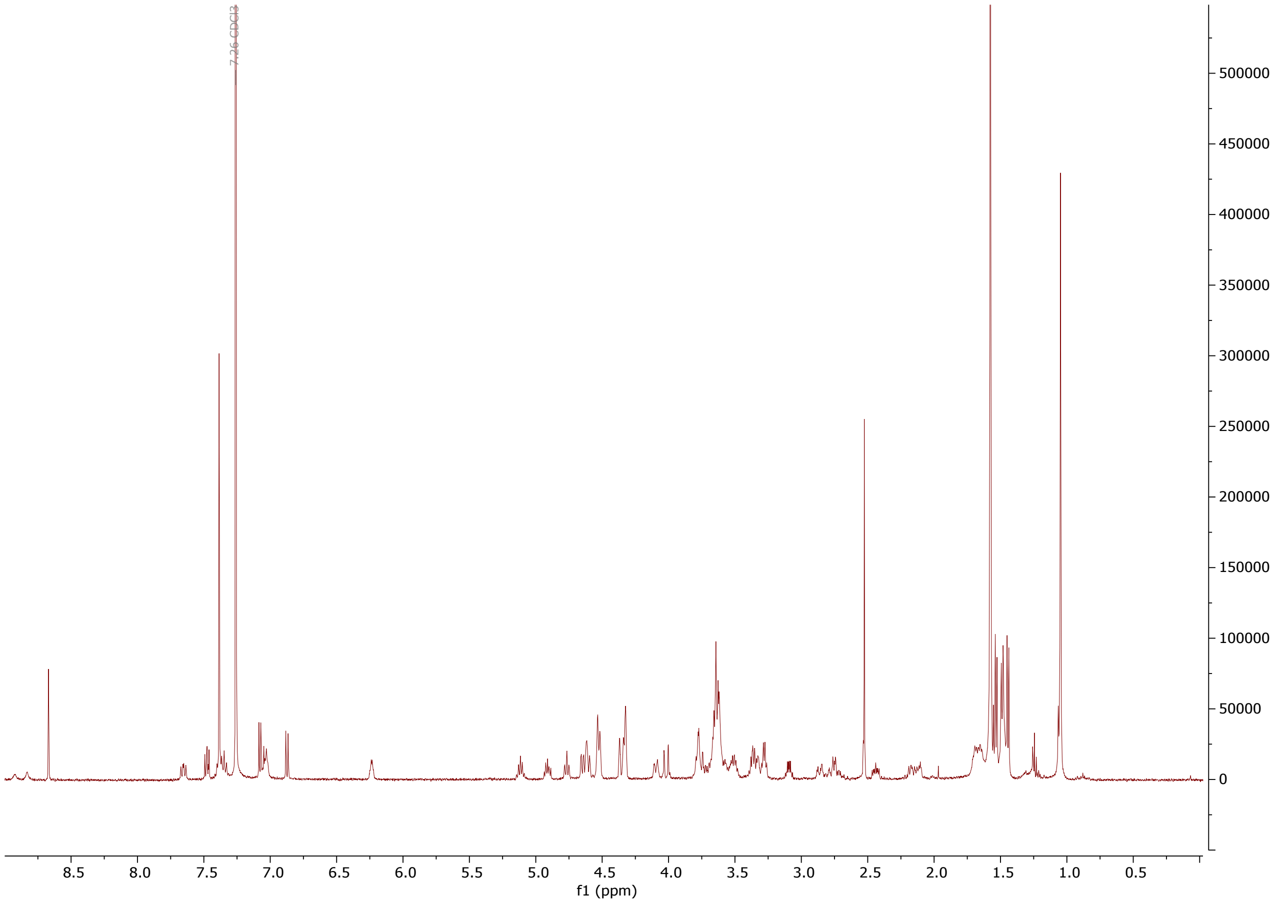

7.26 CDCl3
f1 (ppm)

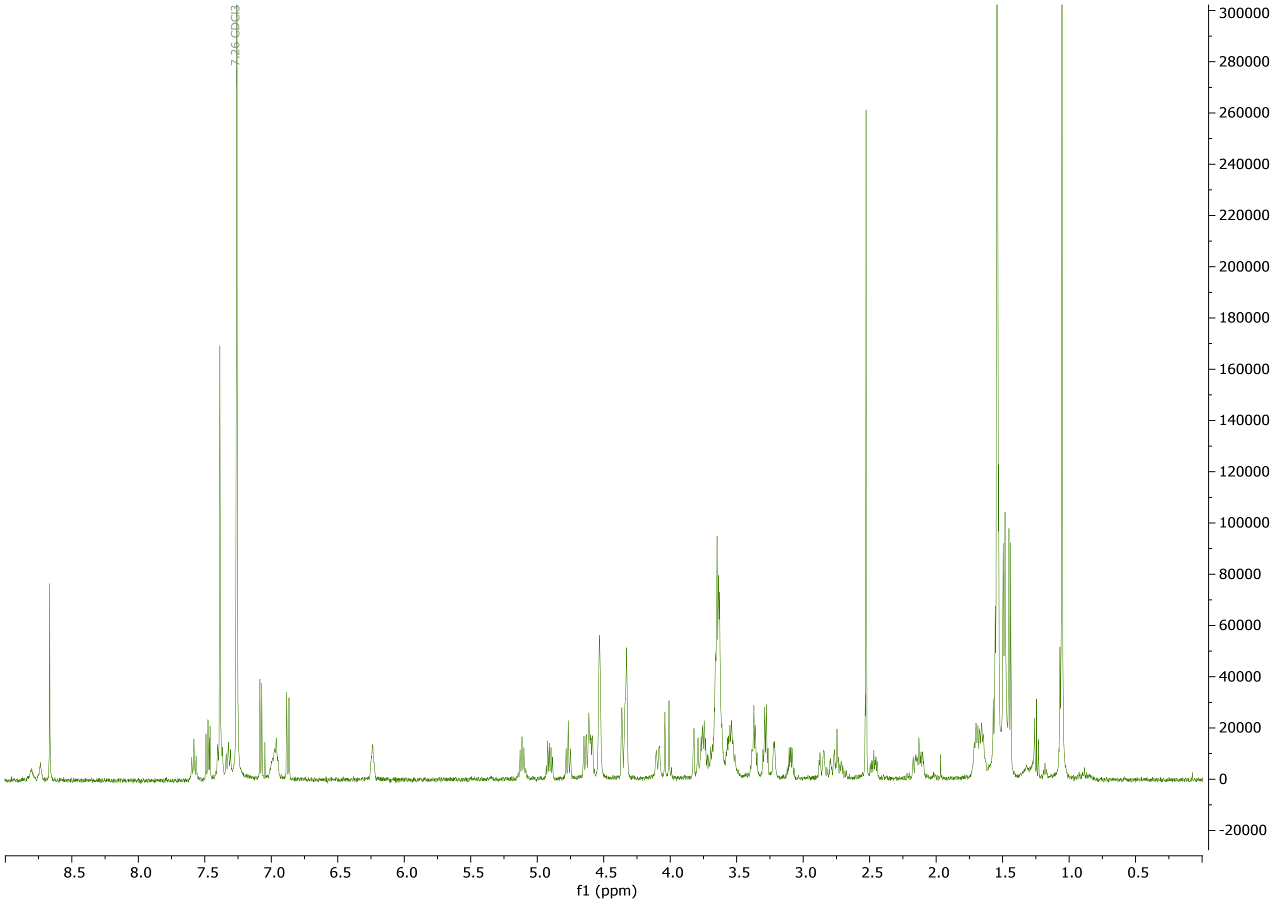

7.26 CDCl3
f1 (ppm)

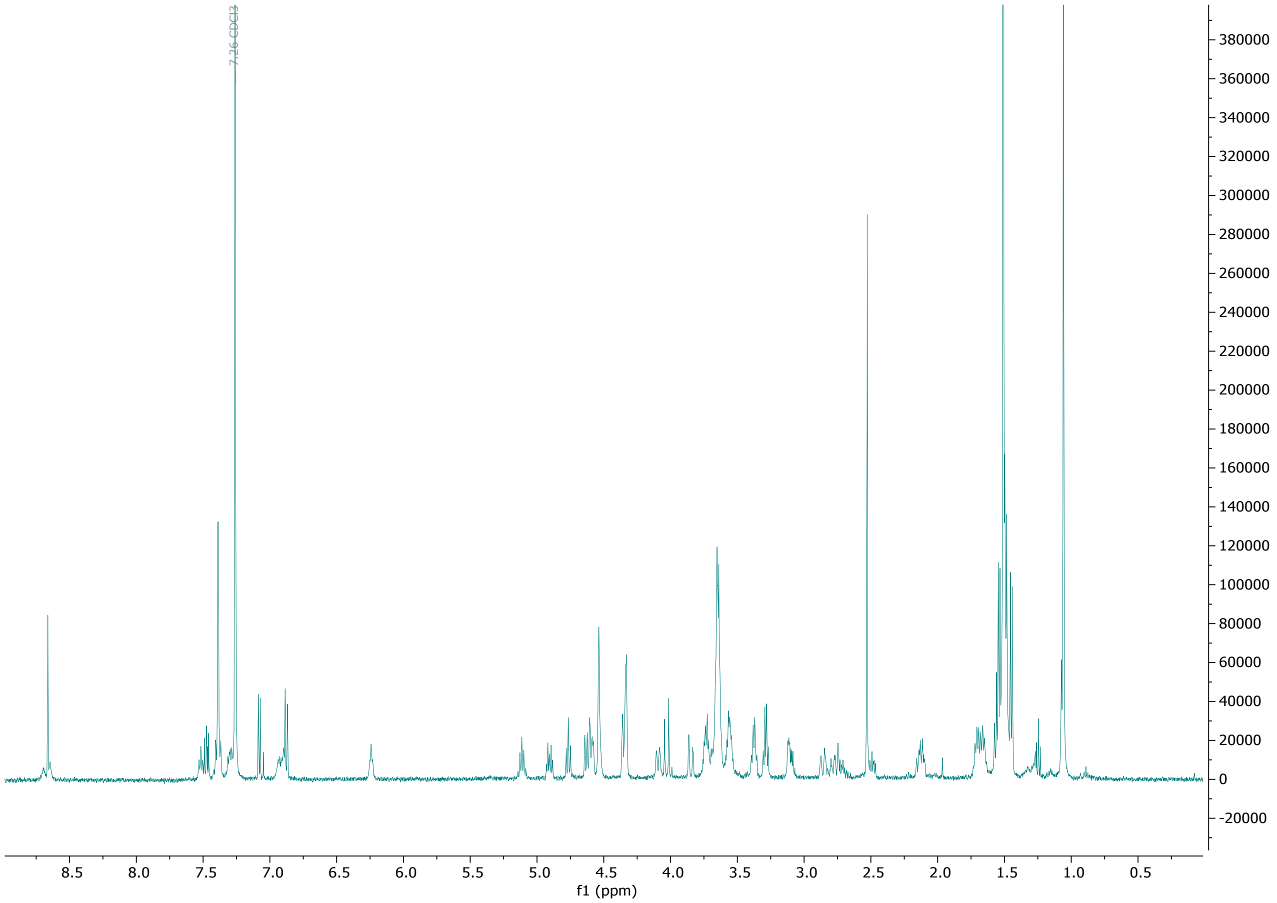

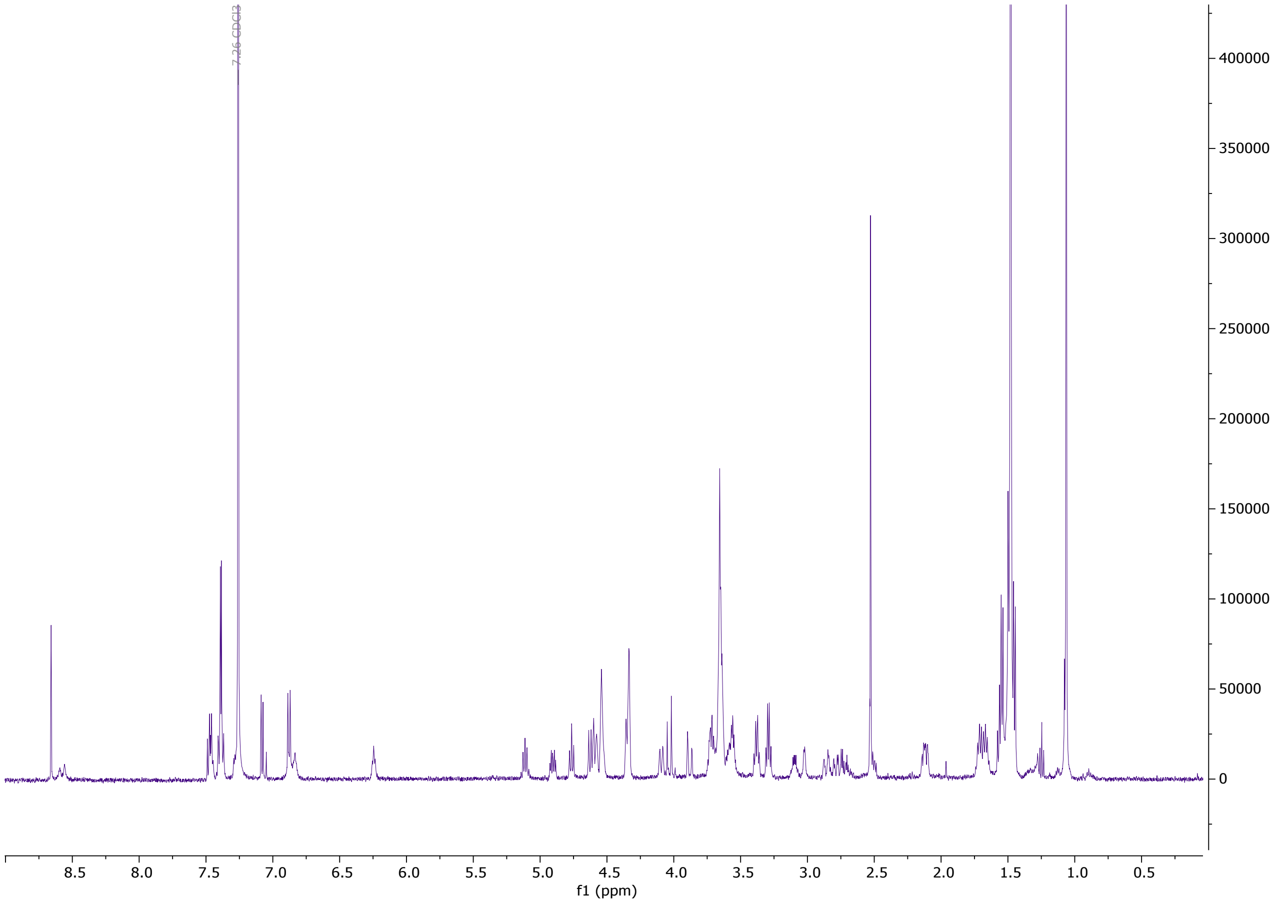

Figure S2B.
Full spectra of **AS4** recorded at 298K (in red), 308K (in green), 318K (in blue), 328K (in purple).

Table S2.
Tabulated NOE cross-peaks across the VHL and CRBN ligands.

VHL	CRBN	Solvent
8-Me[a]	71 (rac)[a]	Chloroform-d_3
26 (cis)[a]	63[a]	Chloroform-d_3
26 (cis)[a]	71 (rac)[a]	Chloroform-d_3
18; alkyl chain	4	Chloroform-d_3
18; alkyl chain	55	Chloroform-d_3
18; alkyl chain	56	Chloroform-d_3
18; alkyl chain	62	Chloroform-d_3
18; 52; 53	62	Methanol-d_4
18; alkyl chain	63	Chloroform-d_3
18; alkyl chain	64; unassigned amide	Chloroform-d_3
18; 52; 53	64	Methanol-d_4
23; PEG	18; alkyl chain	Chloroform-d_3
24; ferrocene	64; unassigned amide	Chloroform-d_3
24; ferrocene	54; alkyl	Chloroform-d_3
26	54; alkyl	Chloroform-d_3
30	54; alkyl	Chloroform-d_3
30; ferrocene	64; unassigned amide	Chloroform-d_3

[a]The position of the relevant cross-peak appears to be slightly off-centre from the central peak position. A combination of the racemic centre at C-70 in combination with a *cis*/*trans* isomerisation can result in a small chemical shift change as observed in Figure 3C-E. The main peak position corresponds to the population-averaged chemical shift, whereas the resulting cross-peak likely results from the minor species only.

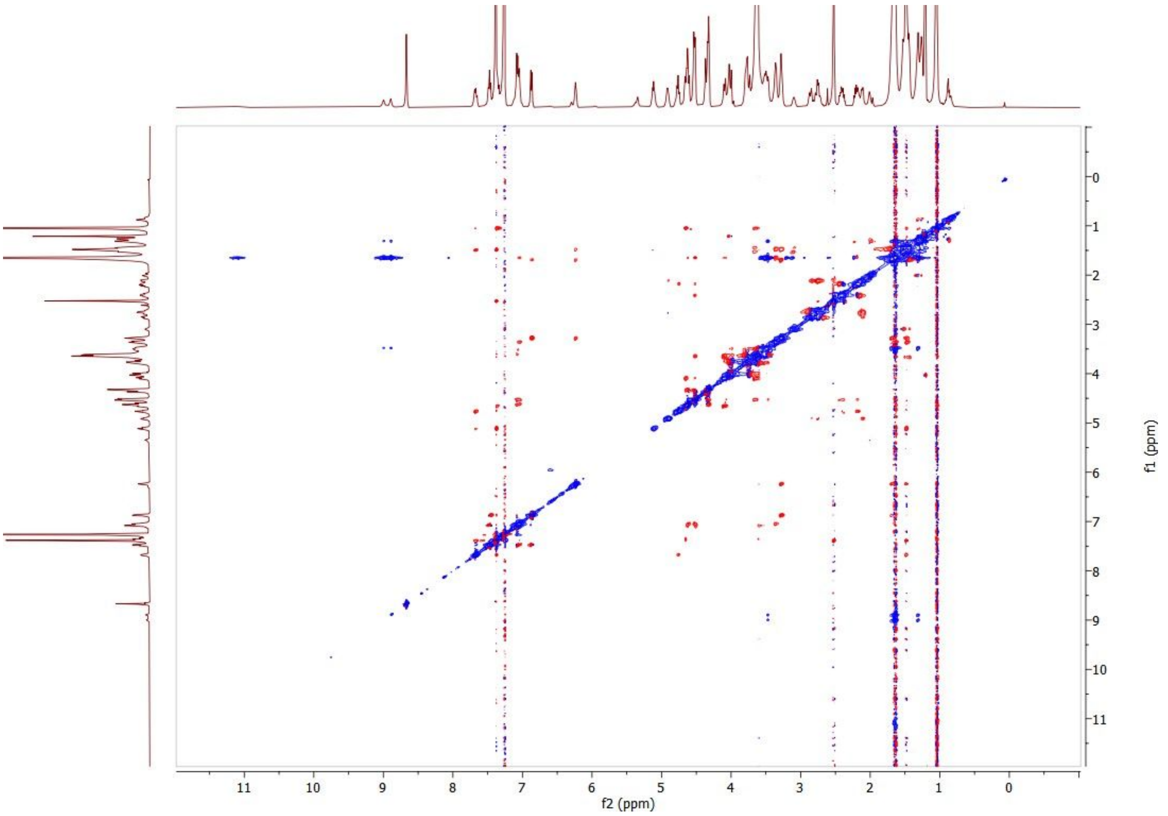

Figure S3A.
Full NOESY spectrum of **AS4** in $CDCl_3$.

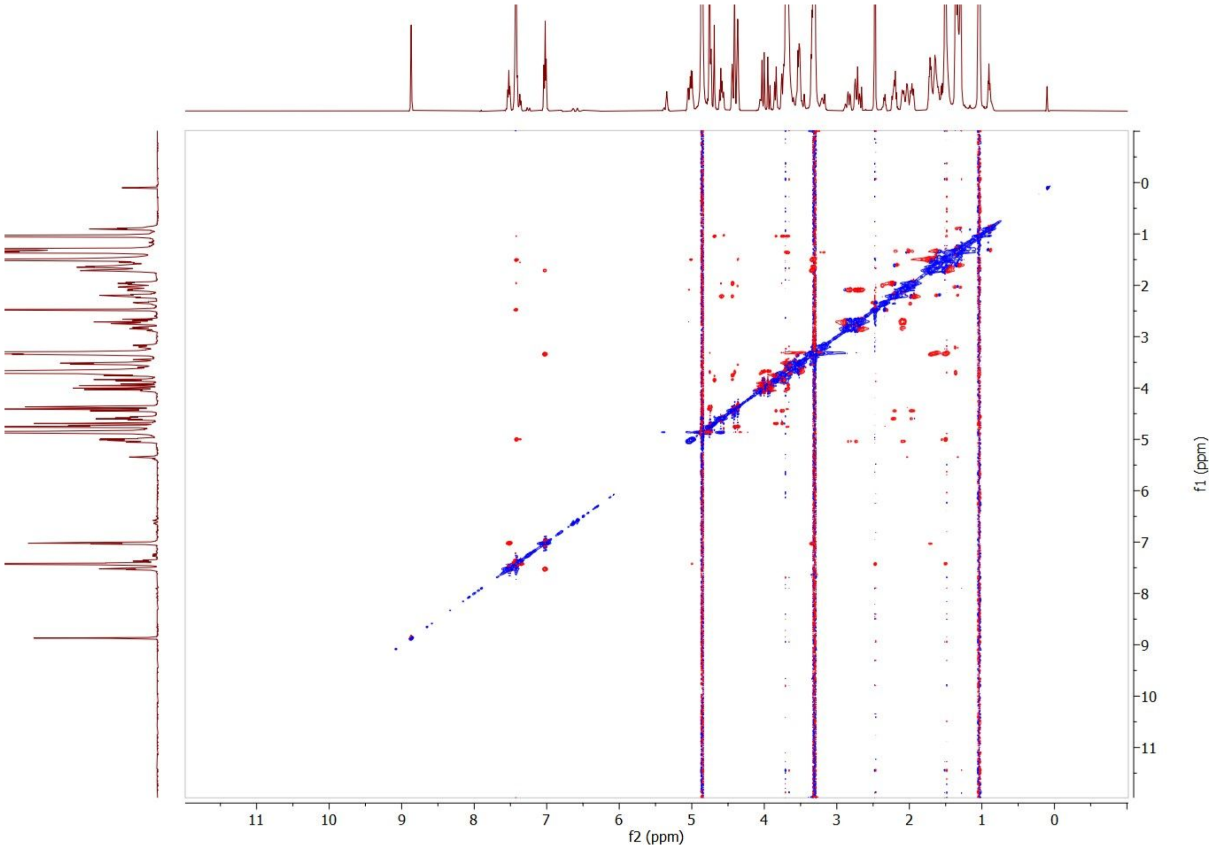

Figure S3B. Full NOESY spectrum of **AS4** in methanol-d_4.

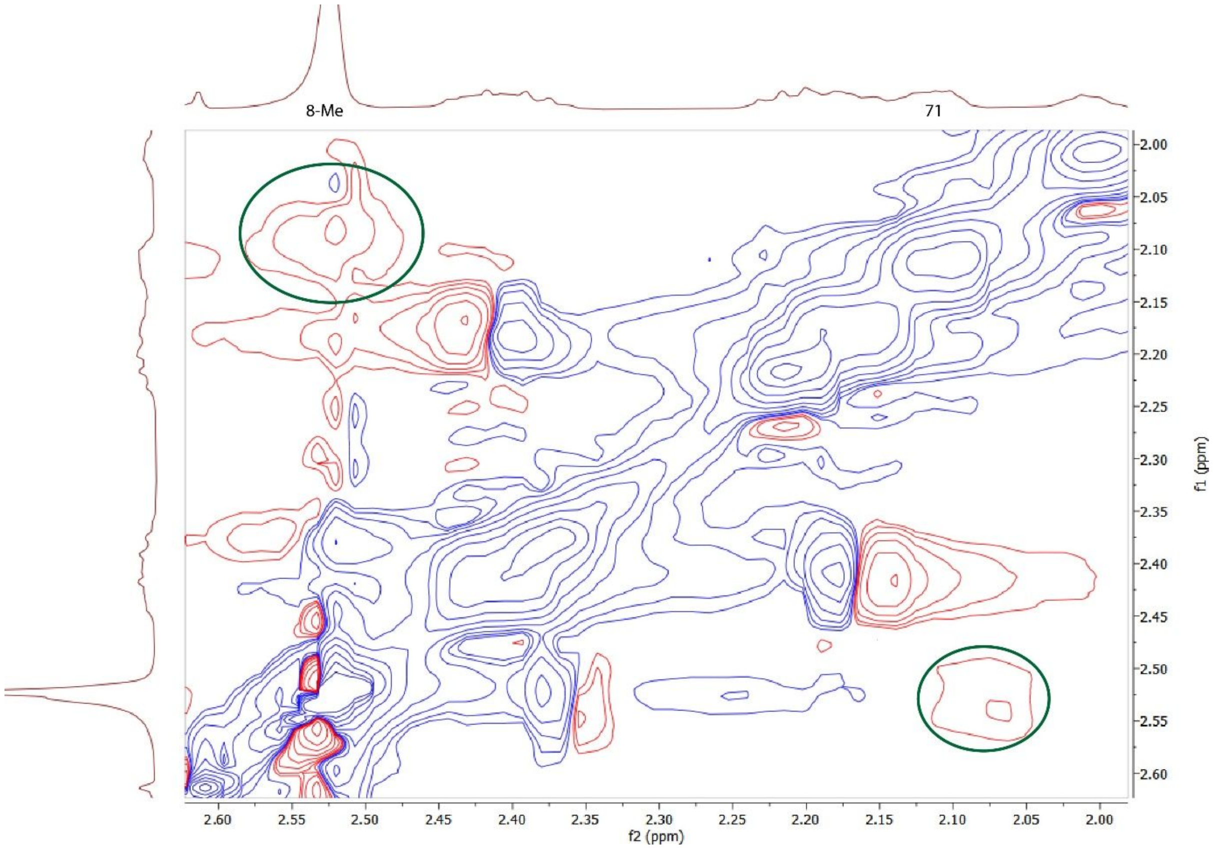

Figure S3C. The NOE correlation of **AS4** between 8-Me and 71 in $CDCl_3$.

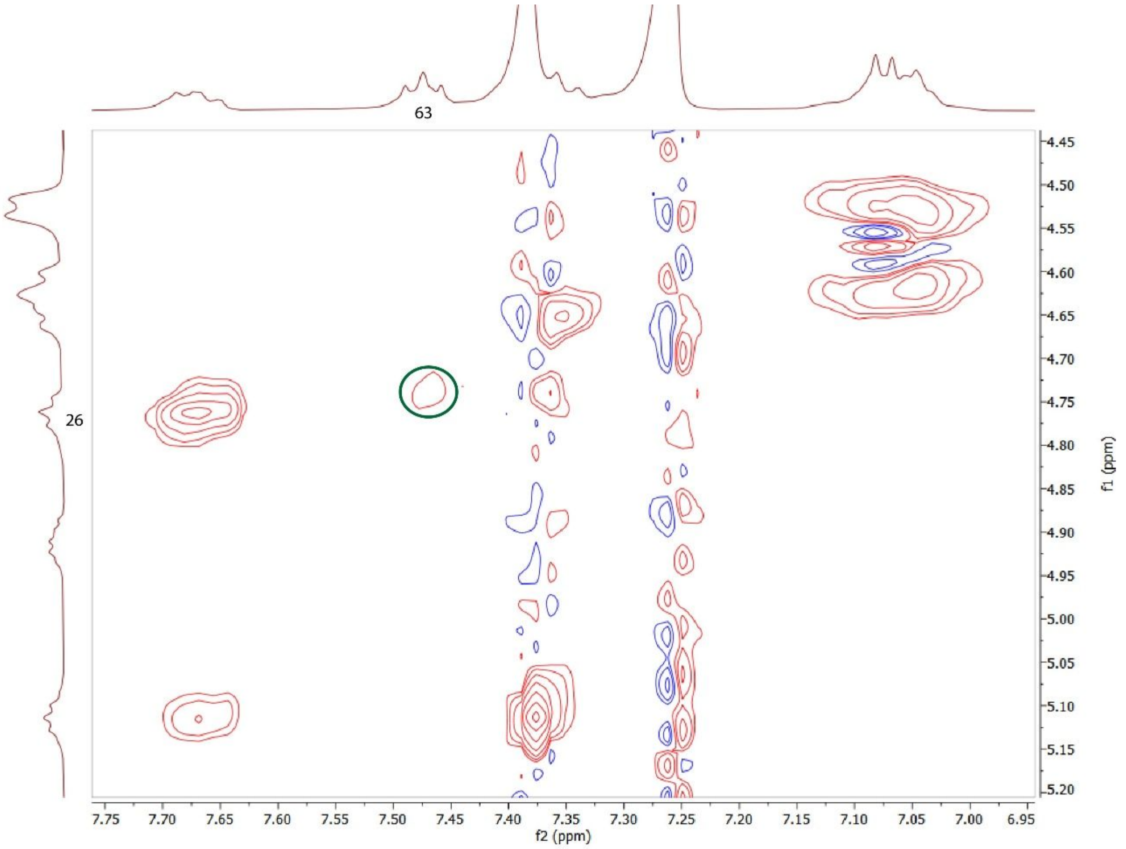

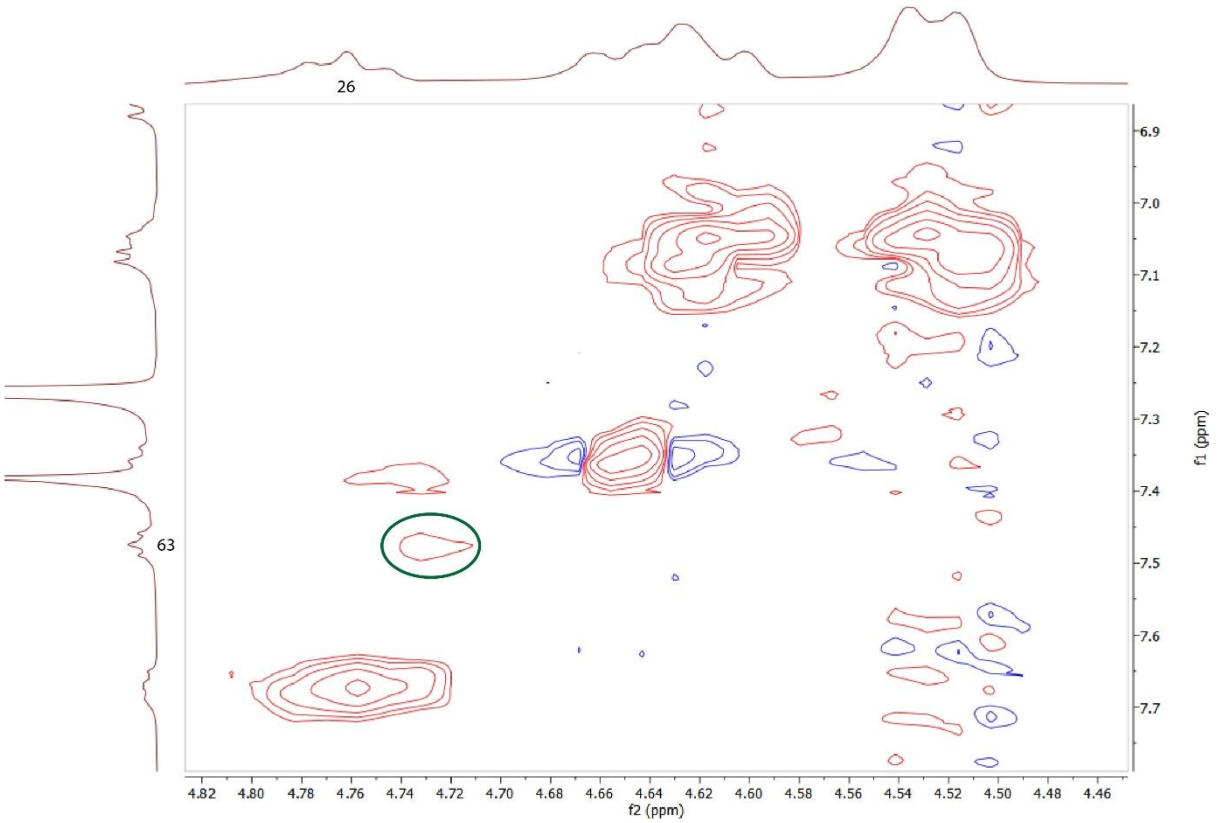

Figure S3D. The NOE correlation of **AS4** between 26 and 63 in $CDCl_3$.

.

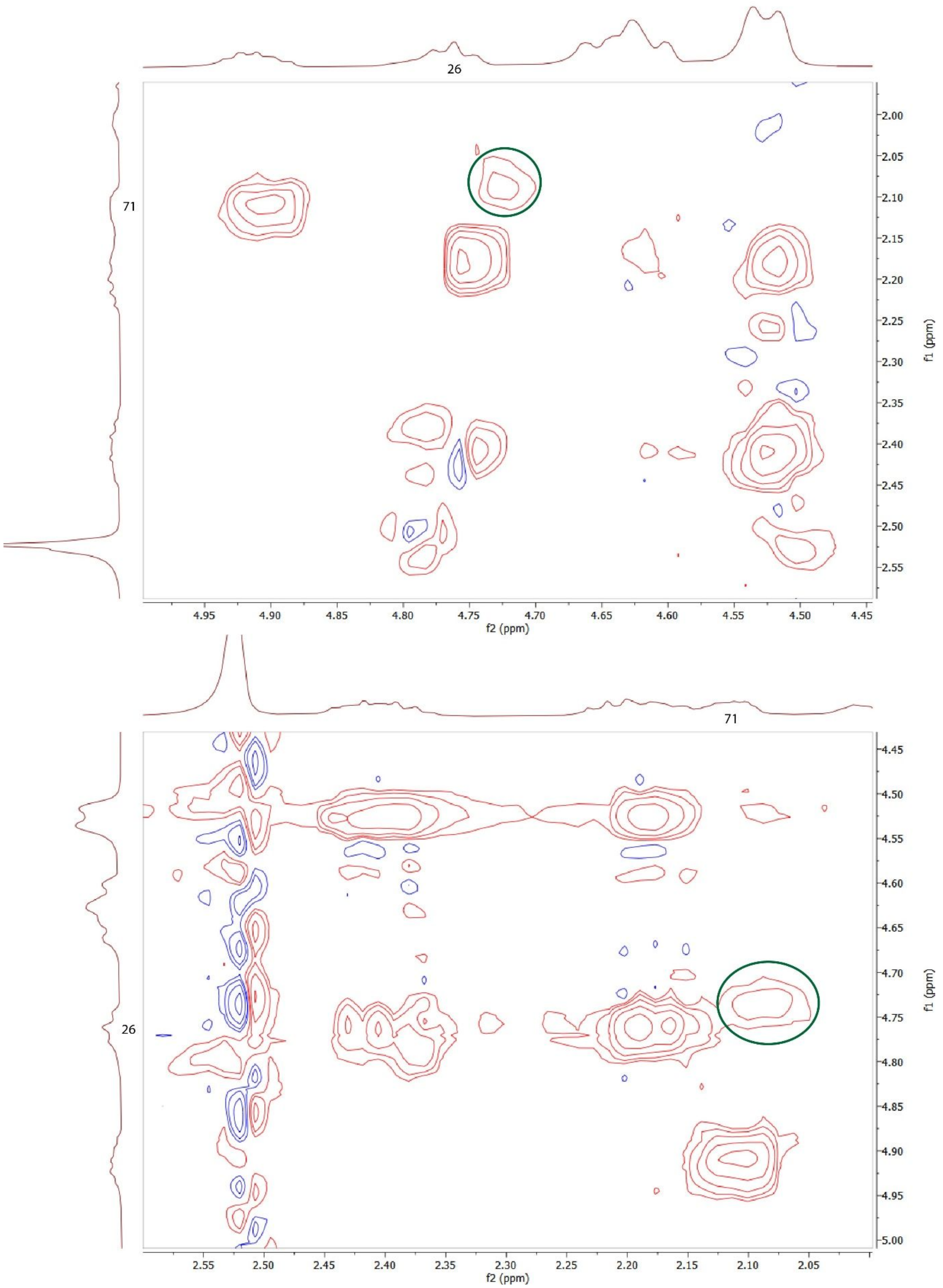

Figure S3E. The NOE correlation of **AS4** between 26 and 71 in $CDCl_3$.

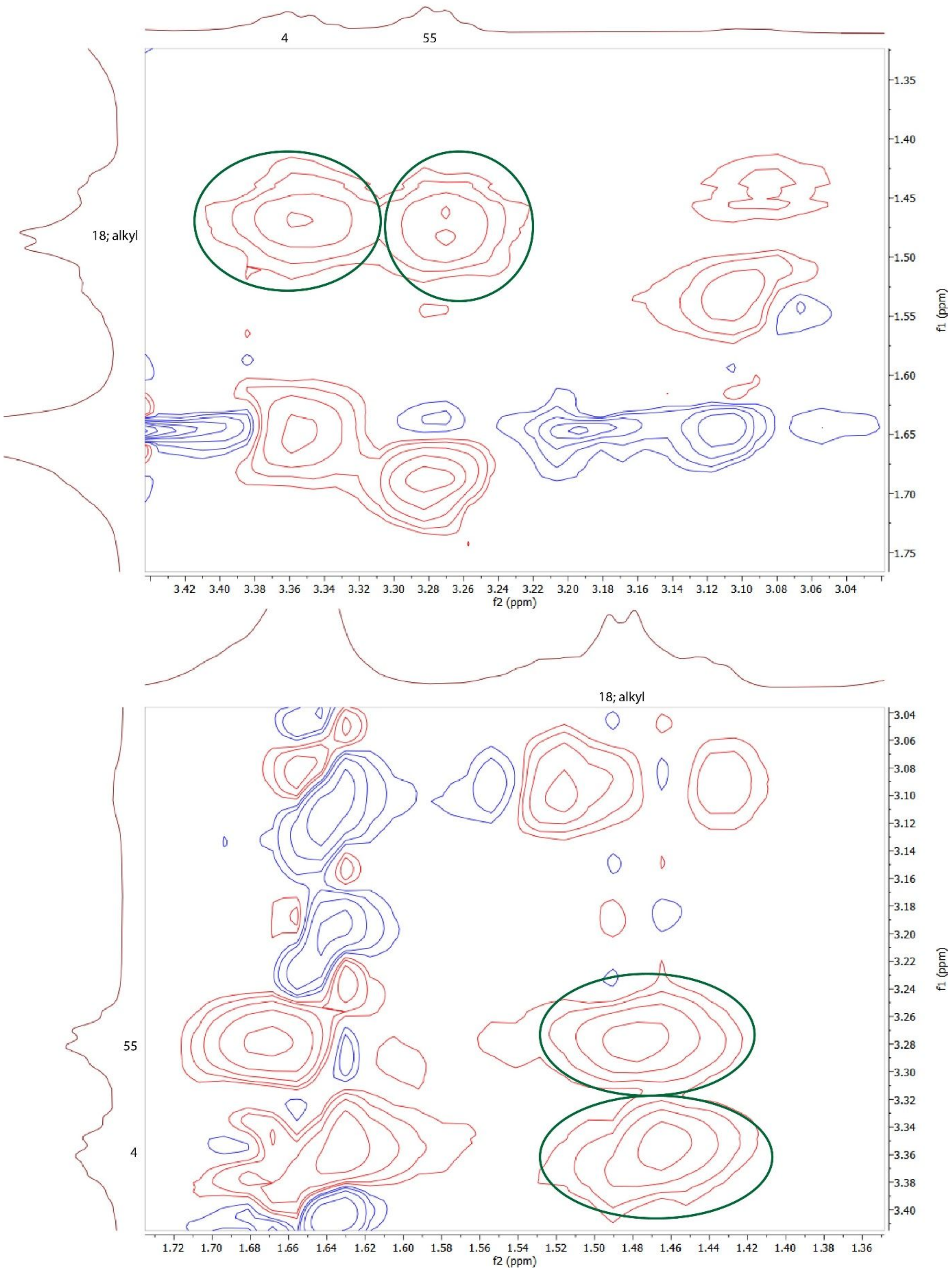

Figure S3F. The NOE correlations of **AS4** between 18; alkyl and 4, and 18; alkyl to 55 in $CDCl_3$.

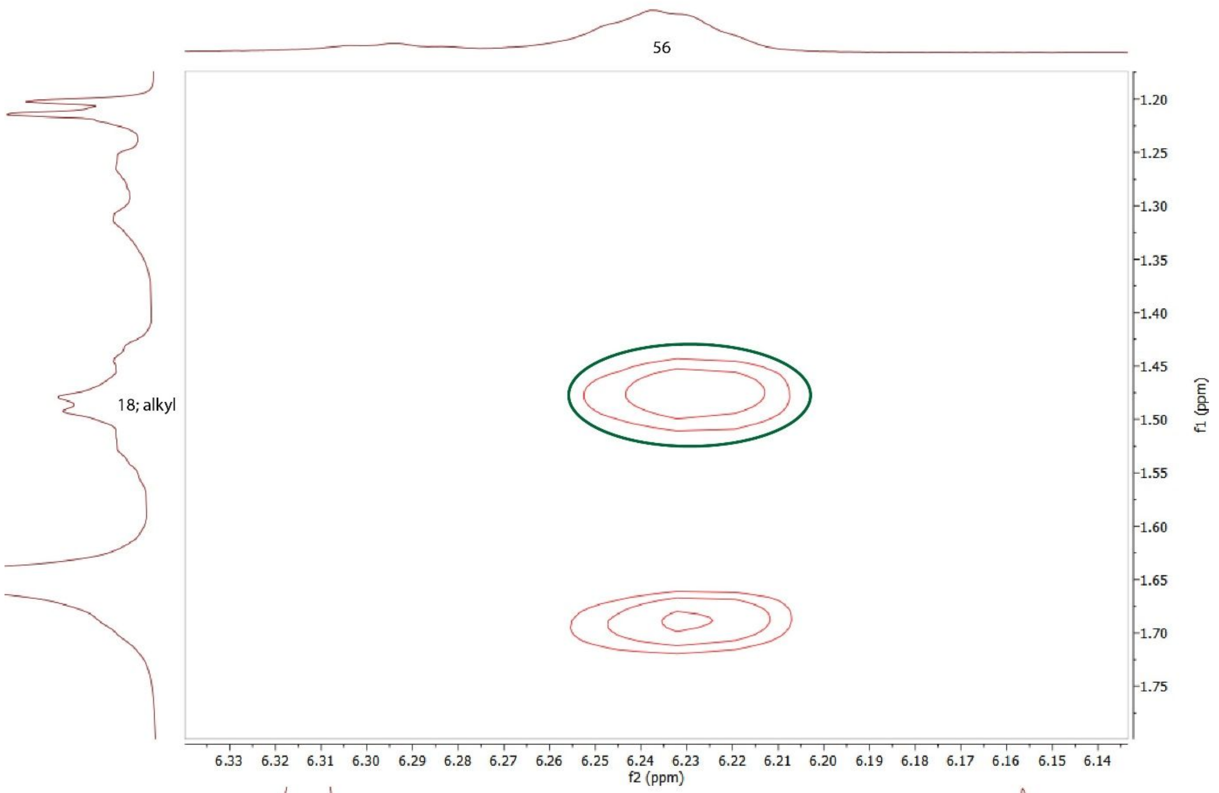

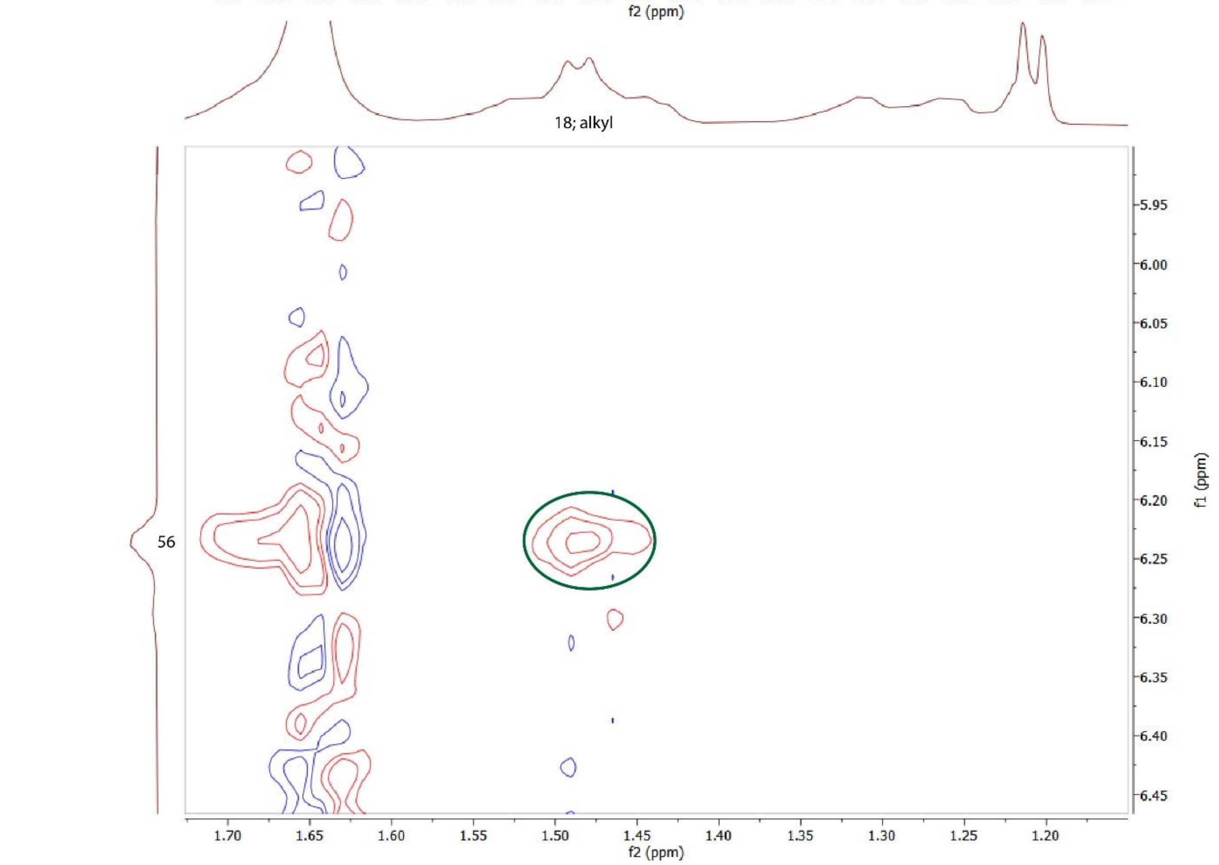

Figure S3G. The NOE correlation of **AS4** between 18; alkyl and 56 in $CDCl_3$.

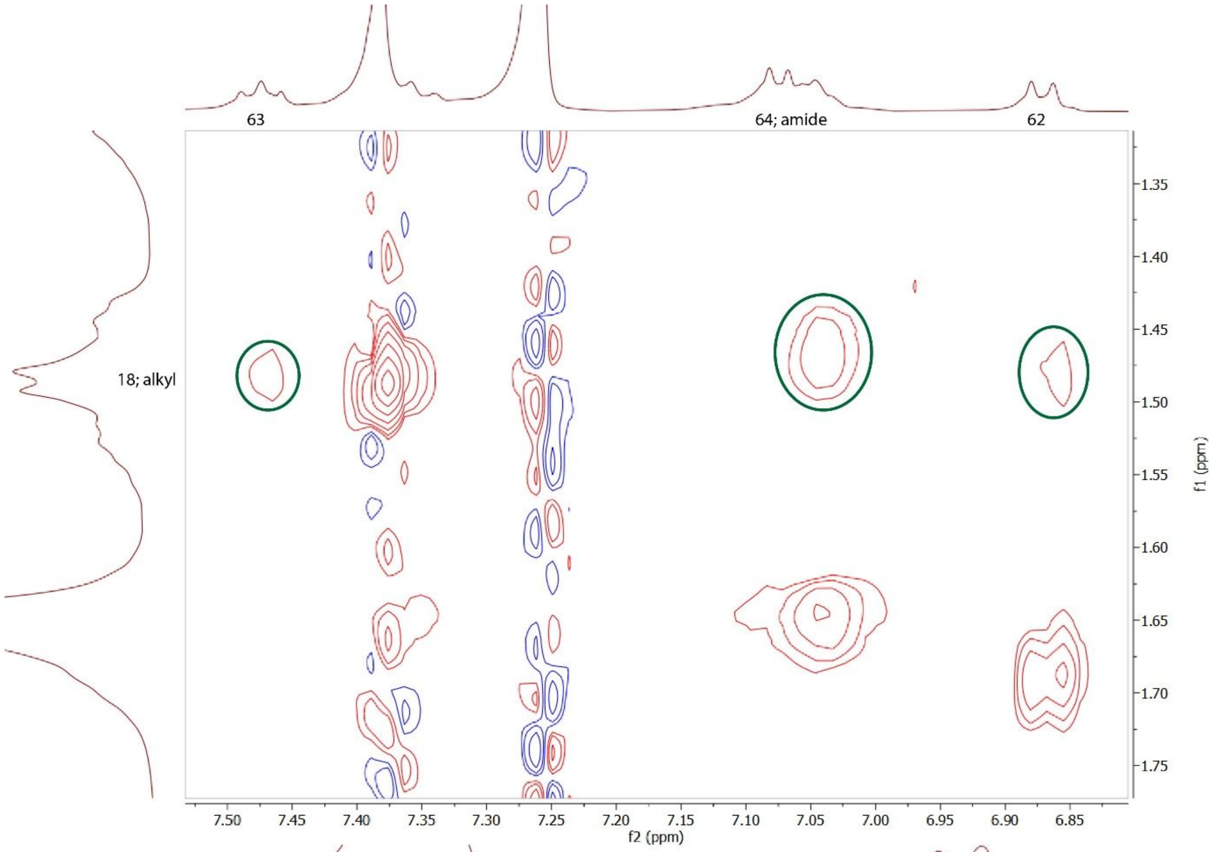

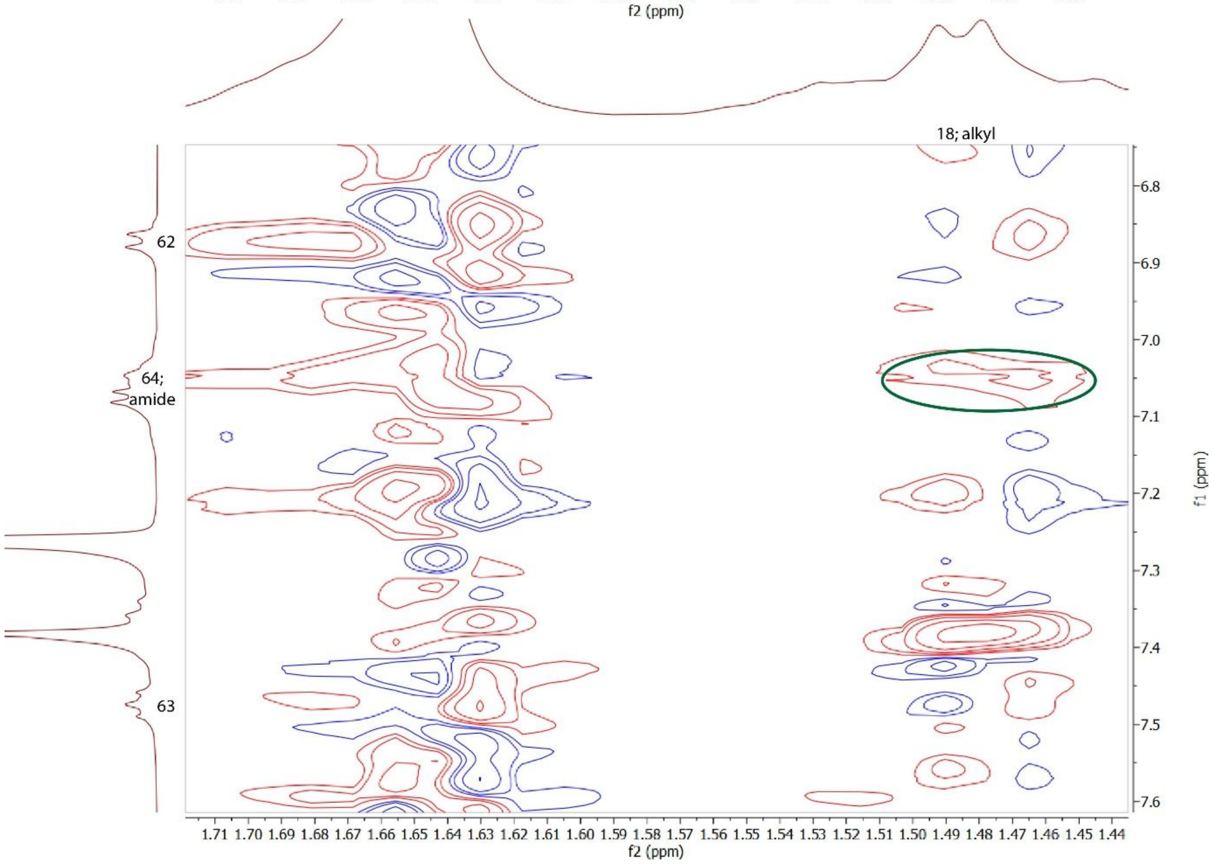

Figure S3H. The NOE correlations of **AS4** between 18; alkyl and 63, 64; amide, and 62 in $CDCl_3$.

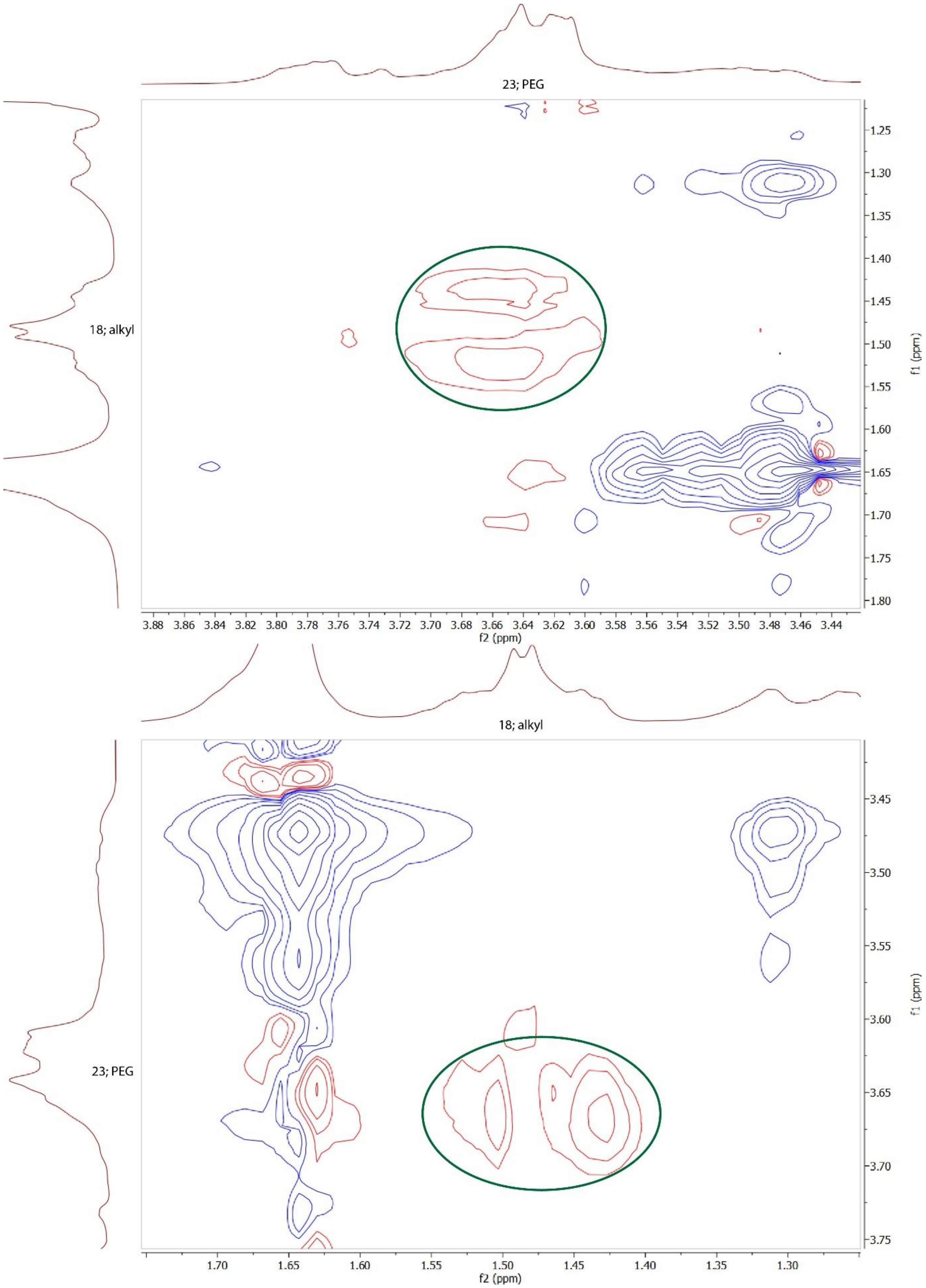

Figure S3I. The NOE correlation of **AS4** between 18; alkyl and 23; PEG in $CDCl_3$.

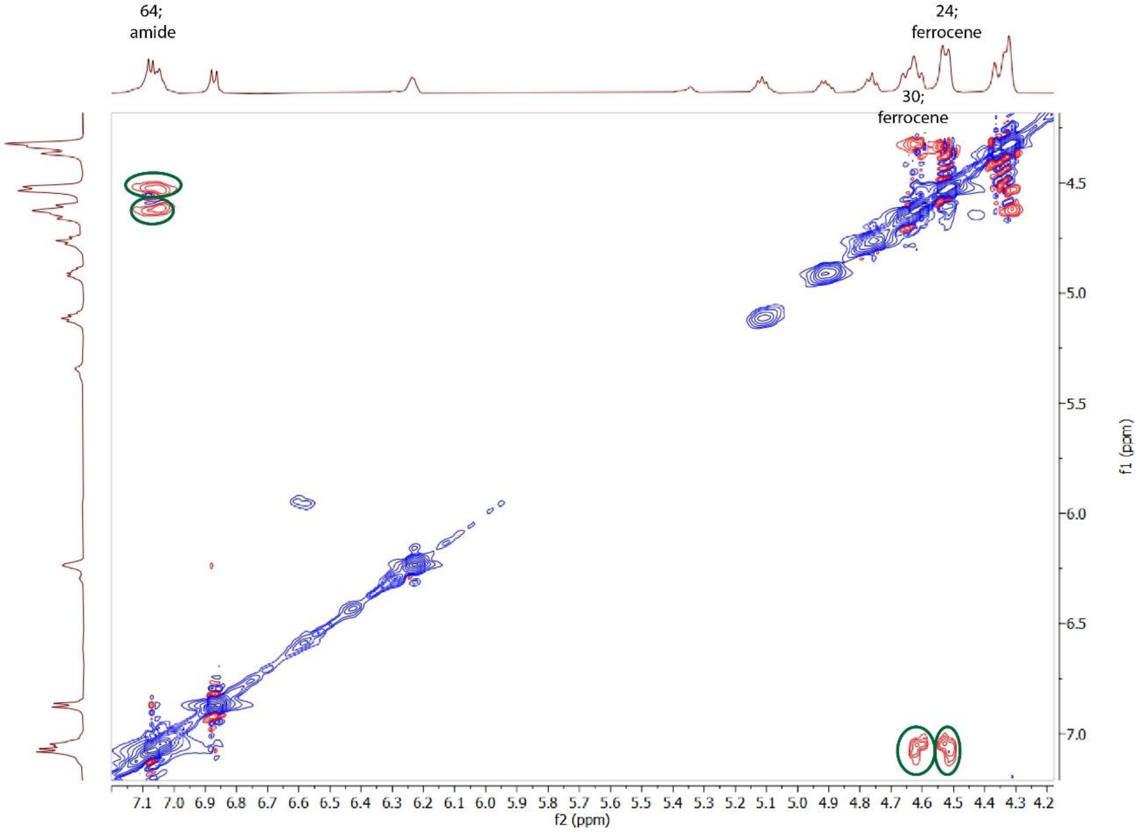

Figure S3J. The NOE correlations of **AS4** between 64; amide 2 or 49 and 24; ferrocene in $CDCl_3$.

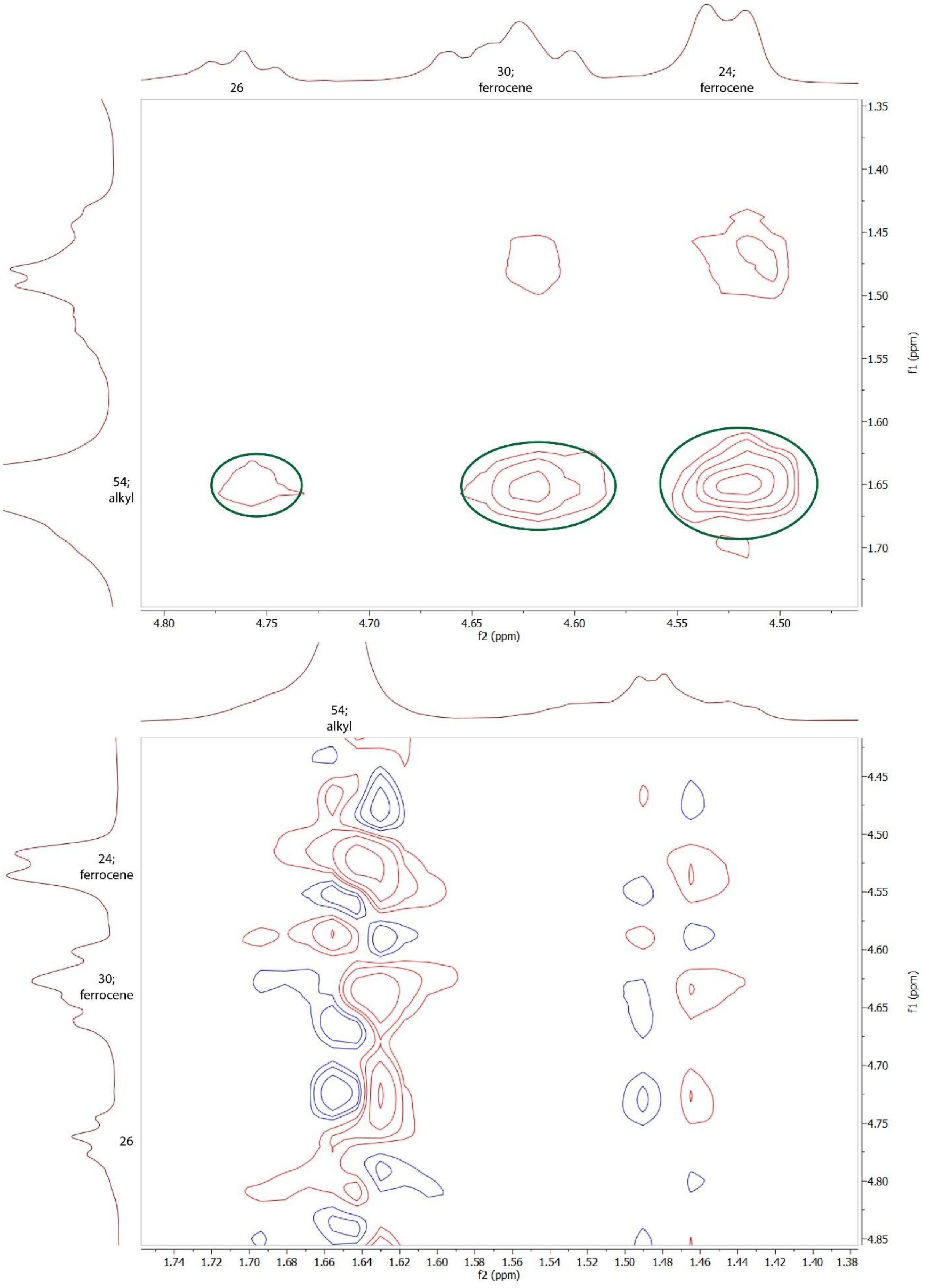

Figure S3K. The NOE correlations of **AS4** between 54[a]; alkyl and 26, 30; ferrocene, and 24; ferrocene in $CDCl_3$.

[a]54 suffers from t1 noise, therefore these cross-peaks can only be observed on one side of the diagonal.

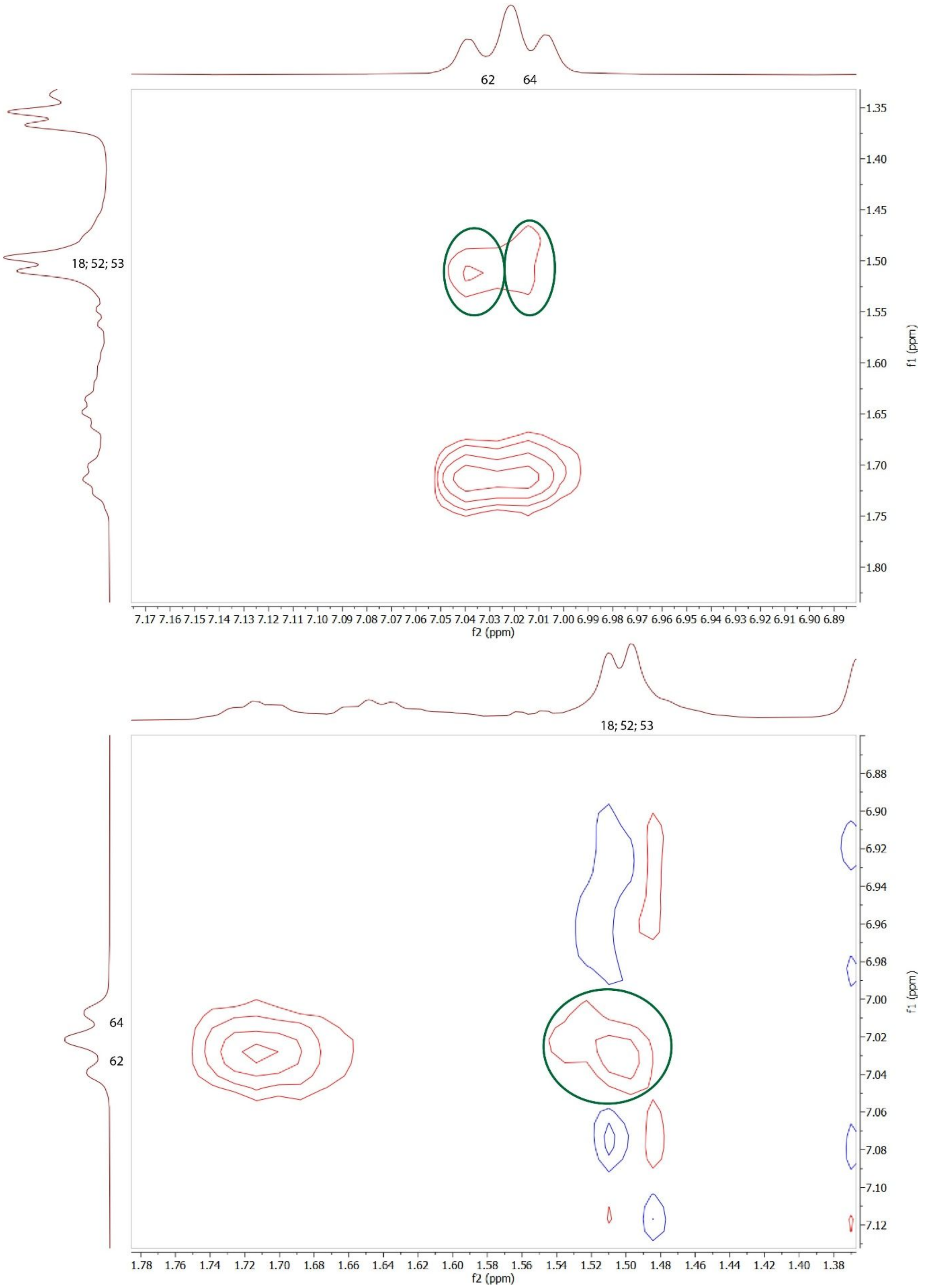

Figure S3L. The NOE correlations of **AS4** between 18; 52; 53 and 62 and 64 in methanol-d_4.

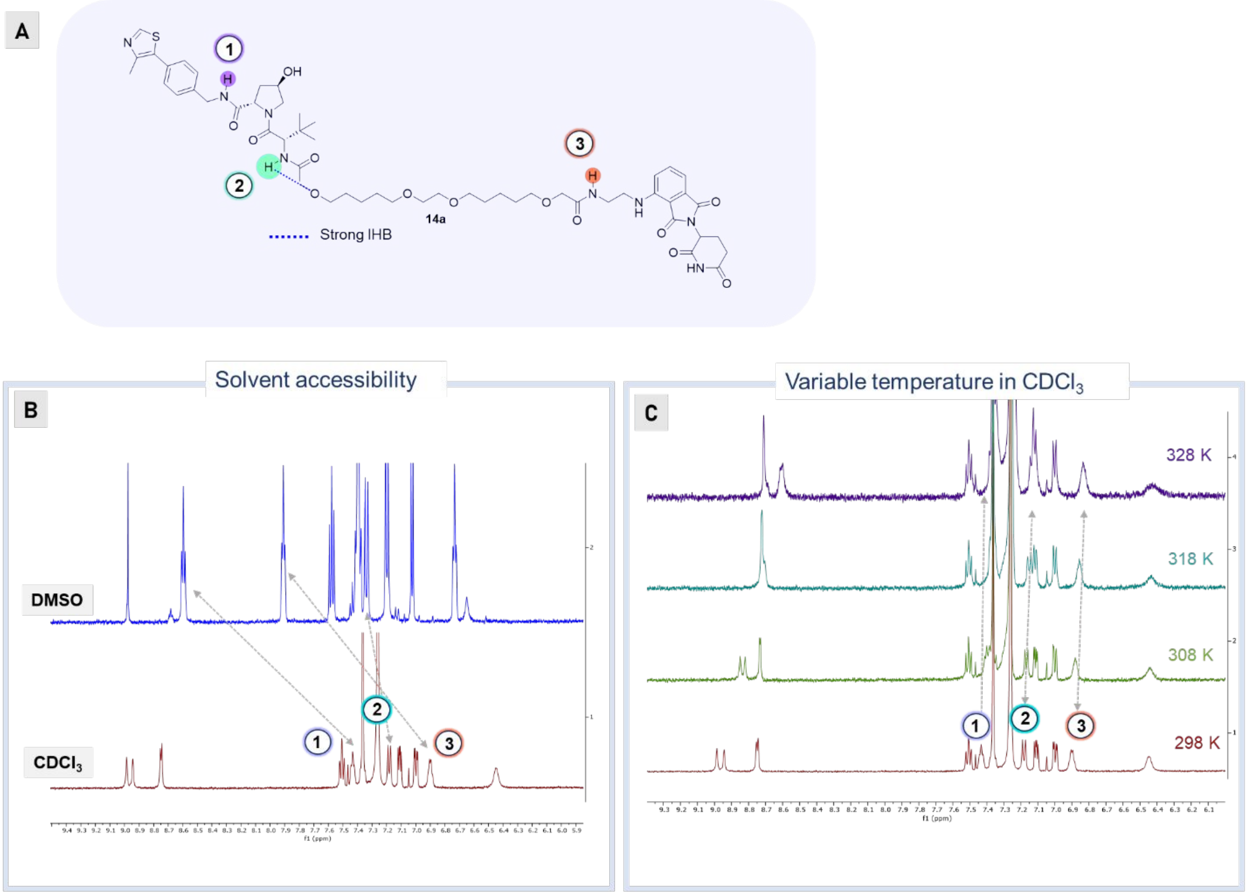

D

		δ/ppm at 298K		Δδ (δ_{DMSO}-δ_{CDCl3}) (ppm)	A_{NMR}	δ/ppm at 328 K	Δδ/ΔT (ppb)
		$CDCl_3$	DMSO-*d6*			$CDCl_3$	
**14a**	N(1)-H	7.43	8.59	1.16	0.16	7.32	-3.51
	N(2)-H	7.18	7.33	0.15	0.03	7.13	-1.57
	N(3)-H	6.89	7.90	1.00	0.15	6.83	-2.12

Figure S4.
Conformational studies on 14a based on HBD shielding analysis. A) Structure of **14a** with numbering of amide protons. **B**) NMR spectra recorded in $CDCl_3$ and DMSO-d_6 to evaluate solvent accessibility of the amide protons N(*1*-*3*)-H. **C**) Changes in chemical shifts (Δδ) observed in vt-NMR experiment of **14a** in $CDCl_3$ (*c* = 1.24 mM) from 258 to 328 K. **D**) Tabulated chemical shift in $CDCl_3$ and DMSO-d_6 at 298 K and the Δδ; calculated A_{NMR} values, chemical shift at 328 K and temperature coefficient (in ppb).

Biological Assays

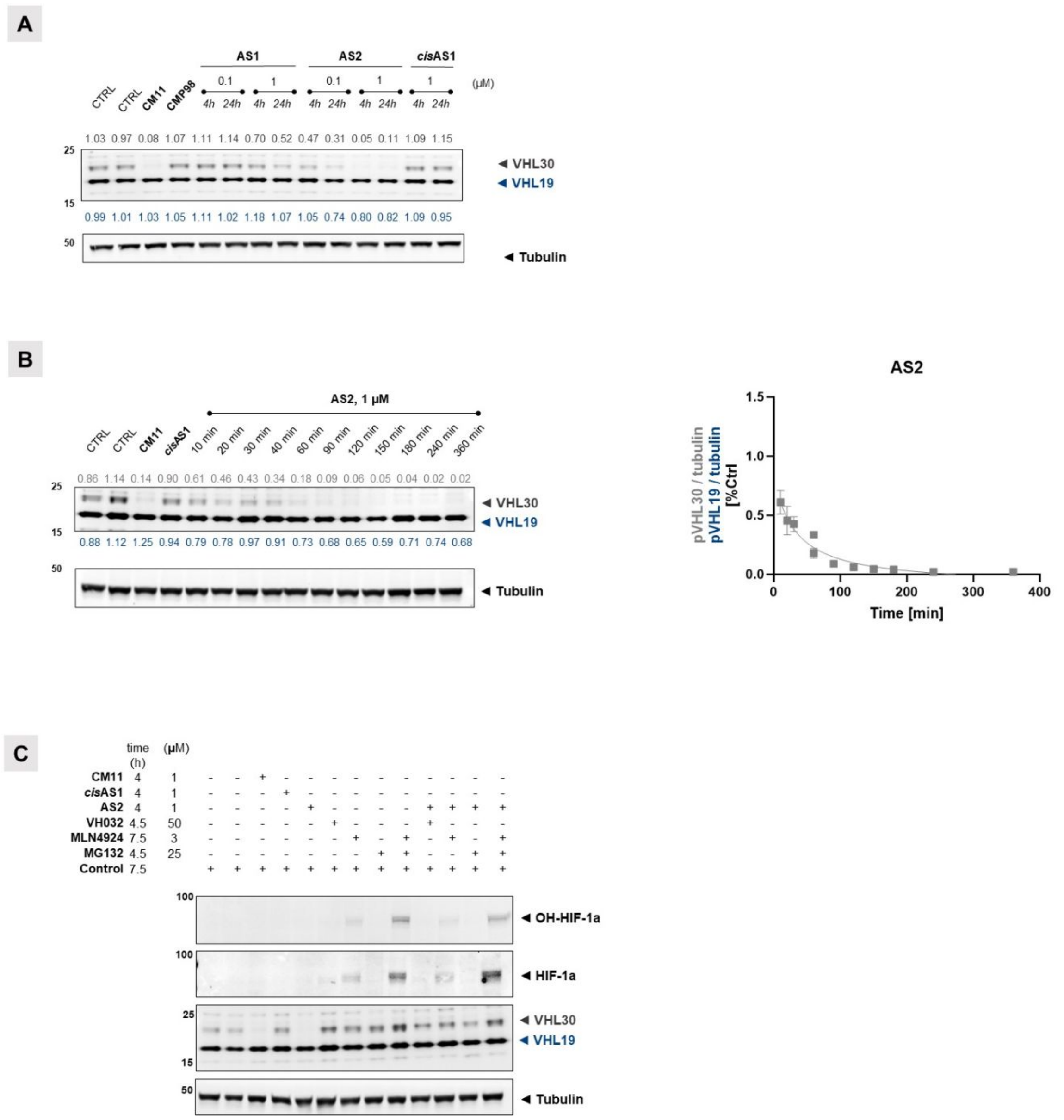

Figure S5.

Homo-FerroTACs AS1 and AS2 induce knockdown of both VHL isoforms in a time-, proteosome- and ubiquitin-dependent mechanism. **A)** Representative immunoblots of VHL protein levels after treatment of HEK293 cells. Cells were treated with 0.1% EtOH, 0.1 µM or 1 µM of the indicated compounds for 4 and 24 h. Controls were included at 1 µM for 24 h. Mean values of two biologically independent experiments. **B)** Representative time-course immunoblots of VHL protein levels after treatment of HEK293 cells treated with 1 µM of **AS2** up to 6 h. Cells were treated with 0.1% EtOH and controls at 1 µM. Mean values of two biologically independent experiments. Apparent half-life values ($t_{1/2}$) were estimated by fitting band intensity against time using a single-phase exponential decay model. **C)** Representative immunoblots of VHL protein levels in HEK293 cells after treatment with **AS2** in the absence or presence of proteasome inhibitor **MG132** (25 µM), neddylation inhibitor **MLN4924** (3 µM) and VHL inhibitor VH032 (50 µM). The amount of EtOH (0.1%) and DMSO (0.5%) is fixed in all samples. Band quantification was performed using ImageJ software and reported (in black and black) as relative amount as ratio of each protein band relative to the lane's loading control (tubulin). VHL/tubulin protein ratios were normalised to the average of the controls (100%).

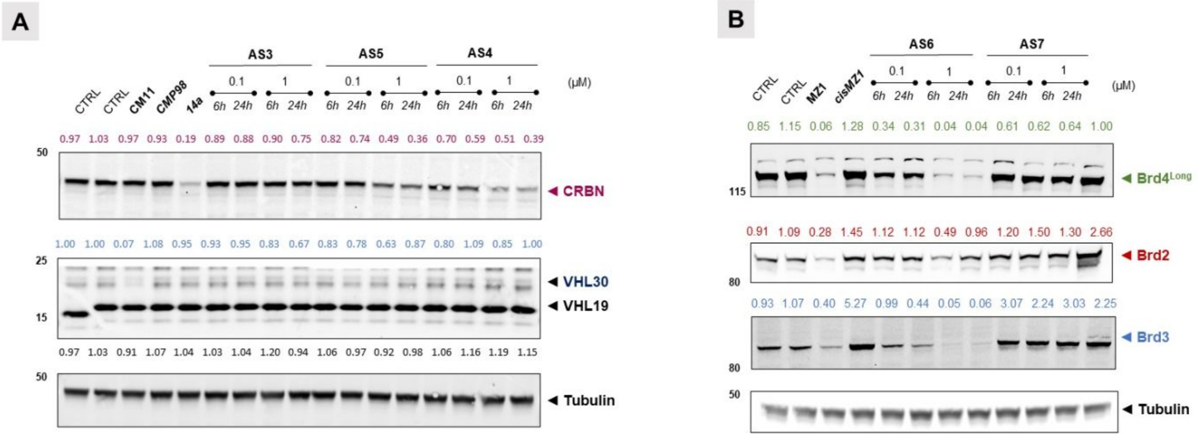

Figure S6

Initial degradation screening on HEK293 cells.

A) Representative immunoblots of VHL and CRBN protein levels after treatment of HEK293 cells with FerroTACs **AS3**, **AS5**, **AS4**. Cells were treated with 0.1% EtOH, 0.1 µM or 1 µM of the indicated compounds for 6 and 24 h. Controls were included at 1 µM for 24 h. Mean values of two biologically independent experiments. **B)** Representative immunoblots of BRD2, BRD3 and BRD4 protein levels after treatment of HEK293 cells with FerroTACs **AS6** and **AS7**. Cells were treated with 0.1% EtOH, 0.1 µM or 1 µM of the indicated compounds for 6 and 24 h. Controls were included at 1 µM for 24 h. Mean values of two biologically independent experiments. Band quantification was performed using ImageJ software and reported as relative amount as ratio of each protein band relative to the lane's loading control (tubulin). VHL/tubulin protein ratios were normalised to the average of the controls (100%).

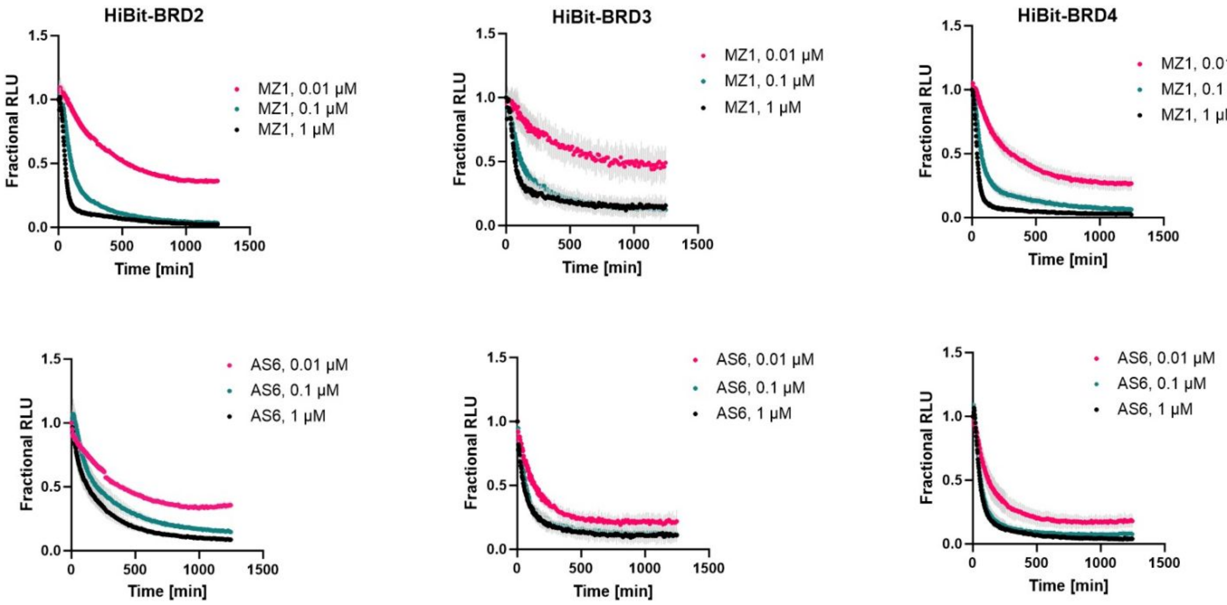

Figure S7.

BET-HiBit Kinetic Degradation.

HiBiT-BRD2, HiBiT-BRD3 and HiBiT-BRD4 HEK293 cells were treated with iPrOH, 1, 0.1 and 0.01 µM of **MZ1** and **AS6**. Luminescence (relative light units (RLU)) was continuously monitored over a 21 h-time (1246 min) period and is plotted normalised to the control as fractional RLU. Apparent half-life values ($t_{1/2}$) were estimated by fitting fractional RLU against time using a single-phase exponential decay model.

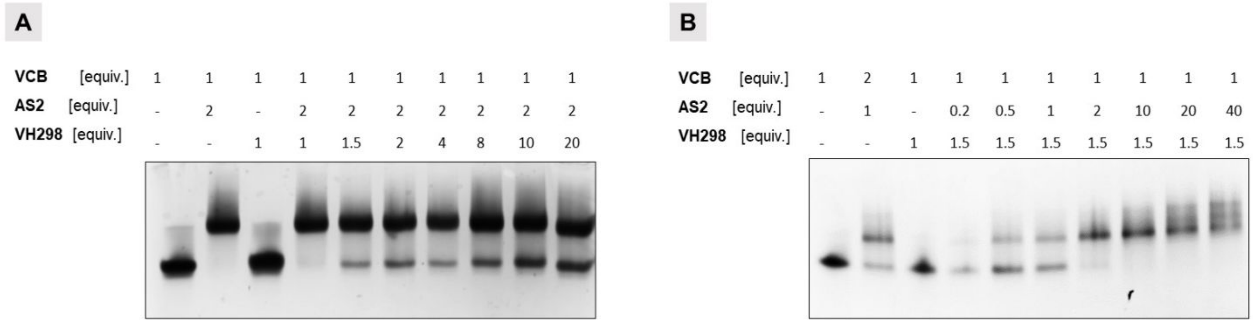

Figure S8.
Native gel electrophoresis assay for ternary complex identification.
A) Representative native gel of competition experiment with VCB (12.11 µM) being pre-incubated with increasing concentration of **VH298** before addition of **AS2** at indicated ratios (24.22 µM). Increasing **VH298** concentration (12.11 to 244.4 µM) was intended to displace **AS2**, favouring the formation of a binary complex as observed by the dose-dependent displacement. **B**) Representative native gel of competition experiment with VCB (2.42 µM) being pre-incubated **VH298** (2.42 or 3.63 µM) before addition of **AS2** at increasing ratios (0.48 to 96.8 µM). A competitive assay was performed by maintaining a fixed concentration of **VH298** while increasing the PROTAC ratio. Solvent concentration was 2.5% DMSO and 2.5% EtOH in each sample. Gels were stained with Instant Blue Coomassie protein stain solution.

To gain additional information about VCB:PROTAC:VCB stability and to assign the migrating bands, competitive assays were performed incubating VCB with increasing concentration of the high-affinity inhibitor **VH298** (Figure S8A) or PROTAC **AS2** (Figure 88B). By increasing the concentration of **VH298**, we aimed to displace PROTAC **AS2** and shift the complex toward the formation of a preferential binary system (lower band, as indicated by incubation with the inhibitor **VH298** alone). In the first competition experiment we observed a dose-dependent displacement mechanism. Notably, even at the highest inhibitor concentration (VCB:PROTAC:VH298, 1:2:20), the band corresponding to the ternary complex (upper band) remained visible, suggesting limited displacement of PROTAC **AS2** from the ternary complex VCB_2:**AS2**. This may indicate a high stability of the complex.
A similar competitive assay was performed by keeping **VH298** at a fixed concentration (VCB:VH298, 1:1.5) while increasing the PROTAC ratio (1:0.2 to 1:40, VCB:PROTAC). In line with the previous experiment, the VCB_2:**AS2**. band corresponding to the ternary complex remained visible even at low PROTAC concentrations (VCB:VH298:PROTAC, 1:1.5:0.5), further supporting the earlier findings.

General Experimental Details

Chemistry.

Reagents and starting materials that are commercially available were purchased from Apollo Scientific, Sigma Aldrich, Fluorochem, TCI and Enamine at reagent grade and were used as received. *N*-Boc derivatives of **VH032**, benzylic methylated **MeVH032** and ***cis*VH032** were all prepared using literature procedures.[1, 2] The reference compounds **VH298**, **CM11**,[3] **CMP98**,[3] **MeCM11**,[4] **14a**,[5] **MZ1**[6] and ***cis*MZ1**[6] were available in-house and synthesized following the referenced procedure. Anhydrous DCM, DMF, dioxane, MeOH, and THF, purchased from Thermo Scientific, were used for synthesis. Solvents for work-up, purification and preparative HPLC were HPLC grade. Thin layer chromatography (TLC) was performed on pre-coated alumina plates (Silica gel 60 F254, Merck) and visualised *via* UV light (UV 254 and/or 365 nm). Flash column chromatography was performed using a Teledyne Isco Combiflash Rf with prepacked Redisep RF Normal phase disposable columns (230 – 400 mesh, 40 – 63 mm: SiliCycle).

High-resolution mass spectrometry (HR-MS) was performed on a Bruker MicroTOF II focus ESI mass spectrometer connected in parallel to a Dionex Ultimate 3000 RSLC system with a diode array detector and a Waters XBridge C18 column (50 mm × 2.1 mm, 3.5 µm particle size). Samples were eluted with a 6 min gradient of 5–95% acetonitrile: water containing 0.1% formic acid at a flow rate of 0.6 mL/min. Liquid chromatography-mass spectrometry (LC-MS) was carried out on a Shimadzu HPLC/MS 2020 equipped with a Hypersil Gold column (1.9 µm particle size, 50 × 2.1 mm), photodiode array detector and ESI detector. Samples were eluted with either a 3 min or 5 min gradient of 5–95% acetonitrile:water containing 0.1% formic acid at a flow rate of 0.7 mL/min.

General Procedure A – HATU-mediated amide coupling.

The desired amine (1.0 equiv) was introduced into a solution of the carboxylic acid (1.0–1.2 equiv) in dry dichloromethane or dry *N,N*-dimethylformamide. To this mixture, HATU (1.5 equiv) and DIPEA (4.0 equiv) were successively added, and the reaction was stirred at room temperature overnight. After completion, the reaction mixture was quenched with water and extracted twice with ethyl acetate. The combined organic phases were washed twice with 5% citric acid solution, twice with saturated sodium bicarbonate solution, and twice with brine. The organic layer was then dried over Na_2SO_4, filtered, and concentrated under reduced pressure. The crude product was purified by flash column chromatography on silica gel.

General Procedure B – Azide reduction.

Azides (1.0 equiv) were dissolved in dry methanol (0.04 M), and a catalytic amount of 10 wt% Pd/C was added. The reaction was stirred under a hydrogen atmosphere (1 atm) at room temperature for 3 hours. After completion, the reaction mixture was filtered through a plug of Celite®, and the filtrate was concentrated to dryness to yield the desired amine, which was used without further purification.

General Procedure C – Acyl chloride formation and conjugation.

Adapted from literature procedure,[7] a mixture of 1,1′-ferrocenedicarboxylic acid (1.0 equiv), thionyl chloride (2.5 equiv), and triethylamine (TEA, 2.0 equiv) was stirred in dry dichloromethane under a nitrogen atmosphere at room temperature. After 2 hours, the solvent was evaporated, and the residue was dissolved in dry heptane. The solution was filtered, and the filtrate was concentrated under reduced pressure, yielding the crude acyl chloride as a red solid. To a solution of this acyl chloride (1.0 equiv) in dry dichloromethane, TEA (3.5 equiv), DMAP (10 mol%), and the appropriate amine (1.5 equiv) were added. The reaction mixture was stirred overnight at room temperature under an inert atmosphere. Upon completion, the mixture was diluted with 15 mL ethyl acetate and washed twice with 5% aqueous citric acid (10 mL each). The organic layer was dried over Na_2SO_4, filtered, and concentrated *in vacuo*. The resulting residue was purified by flash column chromatography on silica gel.

General Procedure D – JQ1 ester formation.

JQ1-carboxylic acid was dissolved in 1 mL of dry dichloromethane under a nitrogen atmosphere, followed by the addition of thionyl chloride (15 equiv). The reaction mixture was stirred at room temperature for 3 hours, with the conversion to the acid chloride monitored by LCMS in methanol [observing the mass of the methyl ester (~443)]. The reaction mixture was then evaporated to dryness, yielding the acyl chloride intermediate

quantitatively. Alcohols (1.0 equiv) were dissolved in dichloromethane (10 mL per mmol) and added to the acyl chloride. After stirring for 18 hours, the reaction was evaporated to dryness, and the residue was purified by flash column chromatography on silica gel.

General Procedure E – CuAAC reaction.

The azide **6c** was added to a suspension of 1′-ethynylferrocene **3**, **4** or **5**, sodium ascorbate and copper sulfate in H_2O (1 mL), *t*BuOH (0.5 mL) and dichloromethane (0.5 mL). The reaction mixture was stirred at room temperature overnight. Upon completion, the reaction was quenched with saturated aqueous sodium bicarbonate and extracted with dichloromethane. The combined organic layers were dried over sodium sulfate and concentrated under reduced pressure. The residue was purified by flash column chromatography on silica gel.

1`-MeVH032-PEG3-ferrocene-1-carboxylic acid (1). Amine **7c** (76 mg, 0.12 mmol) was stirred with DIPEA (60 µL, 0.36 mmol) in 2 mL of dry dichloromethane, then added to a solution of 1,1′-ferrocenedicarboxylic acid (100 mg, 0.36 mmol) in 2 mL of dry dichloromethane, followed by HATU (160 mg, 0.36 mmol, 1.0 equiv). The reaction mixture was stirred at room temperature overnight, then concentrated and purified by normal phase flash column chromatography on silica gel using a linear gradient of 0% to 20% methanol in dichloromethane, yielding compound 1 as a pale yellow oil (44 mg, 33%). ^{1}H NMR (500 MHz, $CDCl_3$) δ 8.60 (s, 1H), 7.55 (s, 1H), 7.28 (d, *J* = 8.9 Hz, 4H), 7.04 (s, 1H), 5.07 – 4.98 (m, 1H), 4.72 (s, 1H), 4.63 (s, 2H), 4.58 (d, *J* = 8.9 Hz, 1H), 4.54 (s, 2H), 4.45 (s, 2H), 4.33 (s, 2H), 4.27 (s, 2H), 4.07 – 3.96 (m, 2H), 3.93 (d, *J* = 15.2 Hz, 1H), 3.61 – 3.53 (m, 12H), 3.41 (s, 3H), 2.44 (s, 3H), 2.33 – 2.21 (m, 1H), 2.18 – 2.04 (m, 1H), 1.41 (d, *J* = 6.8 Hz, 3H), 0.98 (s, 9H). LCMS *m/z* $[M+H]^+$ 890.2.

1′-Ethynylferrocene-1-carboxylic acid (2). Acetyl chloride (96 mg, 1.22 mmol) was added at 0 °C to a suspension of anhydrous aluminium chloride (164 mg, 1.22 mmol) in 1 mL of dichloromethane, and the mixture was stirred for 15 minutes. This mixture was then added over 15 minutes at 0 °C to a solution of methyl ferrocene-1-carboxylate (100 mg, 0.40 mmol) in 1 mL of dichloromethane, stirred under a nitrogen atmosphere, and allowed to stir overnight. The reaction mixture was poured into water and extracted with dichloromethane. The combined organic layers were dried over Na_2SO_4 and concentrated under reduced pressure, yielding 1-acetyl-1′-methoxycarbonylferrocene as an orange solid (102 mg, 87%), used without further characterisation.

To a chilled solution of dry DMF (1.0 mL) at 0 °C, $POCl_3$ (0.138 mL, 1.48 mmol) was added dropwise. After stirring for 15 minutes, the resulting Vilsmeier reagent was added to an ice-cold mixture of 1–acetyl-1′-methoxycarbonylferrocene (102 mg, 0.356 mmol) in anhydrous DMF (0.5 mL) over 15 minutes. The mixture was then warmed to room temperature and stirred for 2 hours. The reaction complex was quenched by adding sodium acetate trihydrate (500 mg, 3.56 mmol), followed by the addition of saturated aqueous sodium bicarbonate until the aqueous phase was neutral. The aqueous phase was extracted with dichloromethane, and the combined organic layers were dried over Na_2SO_4 and concentrated *in vacuo.*

The intermediate product, dissolved in anhydrous dioxane (1 mL), was heated to reflux for 5 minutes and treated with a boiling solution of 0.5 N NaOH (2 mL). The reaction mixture was refluxed for 25 minutes, then cooled with ice water, neutralised with diluted HCl, and extracted with dichloromethane. The organic phase was dried, concentrated under reduced pressure, and purified by normal phase flash chromatography (ethyl acetate/heptane, 1:1), affording compound **2** as a red-orange solid (35 mg, 74% yield over three steps). ^{1}H NMR (500 MHz, $CDCl_3$) δ 4.90 (t, *J* = 2.0 Hz, 2H), 4.52 (t, *J* = 1.9 Hz, 2H), 4.50 (t, *J* = 2.0 Hz, 2H), 4.28 (t, *J* = 1.9 Hz, 2H), 2.80 (s, 1H). LCMS *m/z* $[M+H]^+$ 255.0

1′-Ethynyl-4-((2-aminoethyl)amino)-2-(2,6-dioxopiperidin-3-yl)isoindoline-1,3-dione ferrocene-1-carboxamide (3). Following general procedure A, the desired compound was obtained from **2** (17 mg, 0.07 mmol) and **8*TFA** (28 mg, 0.07 mmol). The crude reaction mixture was purified by flash column chromatography using a gradient of 0% to 10% of methanol in dichloromethane to afford **3** as a light-yellow oil (7 mg, 18 %). The compound used in the next step without further characterization. LCMS *m/z* $[M+H]^+$ 553.2.

1′-Ethynyl-N- 2-(2-(2-acetamidoethoxy)ethoxy)ethyl (S)-2-(4-(4-chlorophenyl)-2,3,9-trimethyl-6H-thieno[3,2-f][1,2,4]triazolo[4,3-a][1,4]diazepin-6-yl)} ferrocene-1-carboxamide (4) . Following general procedure A, the desired compound was obtained from **2** (10 mg, 0.04 mmol) and **10*HCl** (21 mg, 0.041

mmol). The crude reaction mixture was purified by flash column chromatography using a gradient of 0% to 10% of methanol in dichloromethane to afford **4** as a light-yellow oil (22 mg, 69%). ^{1}H NMR (500 MHz, $CDCl_3$) δ 7.40 (d, *J* = 8.3 Hz, 2H), 7.32 (d, *J* = 8.3 Hz, 2H), 6.46 (t, *J* = 5.6 Hz, 1H), 4.70 – 4.65 (m, 2H), 4.65 – 4.58 (m, 1H), 4.44 (t, *J* = 1.9 Hz, 2H), 4.40 – 4.29 (m, 4H), 4.23 – 4.29 (m, 2H), 3.65 – 3.58 (m, 12H), 2.86 (s, 1H), 2.66 (s, 3H), 2.40 (s, 3H), 1.68 (s, 3H). LCMS *m/z* $[M+H]^+$ 768.1

1′-Ethynyl- 3-aminopropyl (S)-2-(4-(4-chlorophenyl)-2,3,9-trimethyl-6H-thieno[3,2-f][1,2,4]triazolo[4,3-a][1,4]diazepin-6-yl)acetate} ferrocene-1-carboxamide (5)

Following general procedure A, the desired compound was obtained from **2** (10 mg, 0.04 mmol) and **11*HCl** (19 mg, 0.04 mmol). The crude reaction mixture was purified by flash column chromatography using a gradient of 0% to 10% of methanol in dichloromethane to afford **5** as colourless oil (5 mg, 18 %). The compound used in the next step without further characterization. LCMS *m/z* $[M+H]^+$ 694.5.

(2*S*,4*R*)-1-((*S*)-14-Azido-2-(tert-butyl)-4-oxo-6,9,12-trioxa-3-azatetradecanoyl)-4-hydroxy-*N*-(4-(4-methylthiazol-5-yl)benzyl)pyrrolidine-2-carboxamide (6a). Following general procedure A, the desired compound was obtained from 2-(2-(2-(2-azidoethoxy)ethoxy)ethoxy)acetic acid (42 mg, 0.18 mmol) and **VH032** (70 mg, 0.15 mmol) in dichloromethane (6 mL). The crude reaction mixture was purified by normal phase flash column chromatography using a gradient of 0% to 6% of methanol in dichloromethane to afford **6a** (78 mg, 81%). ^{1}H NMR ($CDCl_3$, 400 MHz) δ 8.68 (s, 1H), 7.53 – 7.50 (m, 1H), 7.39 – 7.33 (m, 4H), 7.18 (d, *J* = 9.1 Hz, 1H), 5.53 (d, *J* = 9.9 Hz, 1H), 4.74 (d, *J* = 9.0 Hz, 1H), 4.64 (dd, *J* = 7.1, 14.9 Hz, 1H), 4.53 (d, *J* = 9.2 Hz, 1H), 4.50 – 4.45 (m, 1H), 4.30 (dd, *J* = 5.1 Hz, 14.9 Hz, 1H), 4.05 – 3.95 (m, 1H), 3.95 – 3.91 (m, 1H), 3.82 – 3.79 (m, 1H), 3.68 – 3.63 (m, 10H), 3.36 (t, *J* = 5.1 Hz, 2H), 2.52 (s, 3 H), 2.39 – 2.35 (s, 1 H), 2.21 – 2.14 (m, 1H), 0.93 (s, 9H). LCMS *m/z* $[M+H]^+$ 646.6.

(2*S*,4*S*)-1-((*S*)-14-Azido-2-(*tert*-butyl)-4-oxo-6,9,12-trioxa-3-azatetradecanoyl)-4-hydroxy-*N*-(4-(4-methylthiazol-5-yl)benzyl)pyrrolidine-2-carboxamide (6b). Following general procedure A, the desired compound was obtained from 2-(2-(2-(2-azidoethoxy)ethoxy)ethoxy)acetic acid (58 mg, 0.25 mmol) and the amine ***cis*VH032** (100 mg, 0.21 mmol) in DMF (1 mL). The crude was purified by flash column chromatography using a linear gradient of 0% to 6% of methanol in dichloromethane to afford **6b** a colourless oil (55 mg, 40%). ^{1}H NMR (500 MHz, $CDCl_3$) δ 8.71 (s, 1H), 7.55 (t, *J* = 5.8 Hz, 1H), 7.41 (d, *J* = 8.2 Hz, 2H), 7.37 (d, *J* = 8.2 Hz, 2H), 7.21 (d, *J* = 9.1 Hz, 1H), 5.57 (d, *J* = 9.9 Hz, 1H), 4.76 (d, *J* = 9.0 Hz, 1H), 4.67 (dd, *J* = 14.9, 7.1 Hz, 1H), 4.56 (d, *J* = 9.2 Hz, 1H), 4.54 – 4.47 (m, 1H), 4.33 (dd, *J* = 14.9, *J* = 5.1 Hz, 1H), 3.95 (dd, *J* = 10.9, 4.2 Hz, 1H), 3.84 (s, 1H), 3.75 – 3.64 (m, 10H), 3.45 – 3.34 (m, 2H), 2.55 (s, 3H), 2.40 (d, *J* = 14.2 Hz, 1H), 2.20 (ddd, *J* = 14.0, 9.1, 4.9 Hz, 1H), 0.96 (s, 9H). LCMS *m/z* $[M+H]^+$ 646.8.

(2*S*,4*R*)-1-((*S*)-14-azido-2-(*tert*-butyl)-4-oxo-6,9,12-trioxa-3-azatetradecanoyl)-4-hydroxy-*N*-((*S*)-1-(4-(4-methylthiazol-5-yl)phenyl)ethyl)pyrrolidine-2-carboxamide (6c). Following general procedure A, the desired compound was obtained from 2-(2-(2-(2-azidoethoxy)ethoxy)ethoxy)acetic acid (22 mg, 0.10 mmol) and the amine **MeVH032** (40 mg, 0.08 mmol) in dichloromethane (1 mL). The crude reaction mixture was purified by flash column chromatography using a linear gradient of 0% to 6% of methanol in dichloromethane to afford **6c** as colourless oil (40 mg, 77%). ^{1}H NMR (500 MHz, $CDCl_3$) δ 8.70 (s, 1H), 7.50 (d, *J* = 7.8 Hz, 1H), 7.43 (d, *J* = 8.3 Hz, 2H), 7.39 (d, *J* = 8.2 Hz, 2H), 7.35 (d, *J* = 8.4 Hz, 1H), 5.16 – 5.06 (m, 1H), 4.78 (t, *J* = 7.8 Hz, 1H), 4.55 (d, *J* = 8.4 Hz, 2H), 4.17 (d, *J* = 11.5 Hz, 1H), 4.05 (d, *J* = 8.8, 2H), 3.76 – 3.66 (m, 10H), 3.62 (dd, *J* = 11.4, 3.7 Hz, 1H), 3.45 – 3.36 (m, 2H), 2.99 (d, *J* = 4.2 Hz, 1H), 2.65 – 2.57 (m, 1H), 2.12 – 2.03 (m, 1H), 1.70 (s, 3H), 1.50 (d, *J* = 6.9 Hz, 3H), 1.10 (s, 9H). LCMS *m/z* $[M+H]^+$ 660.8.

(2*S*,4*R*)-1-((*S*)-14-Amino-2-(*tert*-butyl)-4-oxo-6,9,12-trioxa-3-azatetradecanoyl)-4-hydroxy-*N*-(4-(4-methylthiazol-5-yl)benzyl)pyrrolidine-2-carboxamide (7a). Following general procedure B, the desired compound was obtained from the azide **6a** (78 mg, 0.12 mmol) as a light-yellow oil (61 mg, 83%) and used in the next step without further purification. LCMS *m/z* $[M+H]^+$ 620.7

(2*S*,4*S*)-1-((*S*)-14-Amino-2-(*tert*-butyl)-4-oxo-6,9,12-trioxa-3-azatetradecanoyl)-4-hydroxy-*N*-(4-(4-methylthiazol-5-yl)benzyl)pyrrolidine-2-carboxamide (7b). Following general procedure B, the desired compound was obtained from the azide **6b** (55 mg, 0.08 mmol) as a light-yellow oil (40 mg, 81%) and used in the next step without further purification. LCMS *m/z* $[M+H]^+$ 620.6

(2*S*,4*R*)-1-((*S*)-14-Amino-2-(*tert*-butyl)-4-oxo-6,9,12-trioxa-3-azatetradecanoyl)-4-hydroxy-*N*-((*S*)-1-(4-(4-methylthiazol-5-yl)phenyl)ethyl)pyrrolidine-2-carboxamide (7c). Following general procedure B, the desired compound was obtained from the azide **6c** (40 mg, 0.06 mmol) as a light-yellow oil (20 mg, 52%) and used in the next step without further purification. LCMS *m/z* $[M+H]^+$ 634.8.

4-((2-aminoethyl)amino)-2-(2,6-dioxopiperidin-3-yl)isoindoline-1,3-dione (8*TFA). To a solution of 2-(2,6-dioxopiperidin-3-yl)-4-fluoroisoindoline-1,3-dione (88 mg, 0.30 mmol) in DMSO (3 mL) were added the amine *tert*-butyl (2-aminoethyl)carbamate (57 mg, 0.36 mmol), DIPEA (20 µL, 1.20 mmol) and then the mixture was heated at 110°C. After 18h, the reaction mixture was diluted with water (15 mL) and extracted with ethyl acetate (3 × 15 mL), dried over Na_2SO_4, filtered and concentrated to dryness. To ***N*-Boc-8** dissolved in dichloromethane and methanol, trifluoroacetic acid (10 mL/mmol) was added, and the reaction mixture stirred at r.t. for 2 h. Solvent and the volatiles were removed under reduced pressure to give **8*TFA (28 mg, 29 % over two steps)**. ^{1}H NMR (500 MHz, CD_3OD) δ 7.68 – 7.59 (m, 1H), 7.17 (dd, *J* = 11.5, 5.9 Hz, 2H), 5.10 (dd, *J* = 12.4, 5.4 Hz, 1H), 3.70 (t, *J* = 6.0 Hz, 2H), 3.22 (t, *J* = 6.0 Hz, 2H), 2.98 – 2.66 (m, 3H), 2.14 (dd, *J* = 9.1, 3.6 Hz, 1H). LCMS *m/z* $[M\text{-}TFA+H]^+$ 317.2.

Tert-butyl (6-((2-(2,6-dioxopiperidin-3-yl)-1,3-dioxoisoindolin-4-yl)amino)hexyl)carbamate (*N*-Boc-9). To a solution of 2-(2,6-dioxopiperidin-3-yl)-4-fluoroisoindoline-1,3-dione (200 mg, 0.72 mmol) in DMSO (3 mL) were added the amine tert-butyl (6-aminohexyl)carbamate (157 mg, 0.72 mmol), DIPEA (50 µL, 2.89 mmol), and then the mixture was heated at 110°C. After 18h, the reaction mixture was diluted with water (15 mL) and extracted with ethyl acetate (3 × 15 mL), dried over Na_2SO_4, filtered and concentrated to dryness. The residue was purified by flash column chromatography using heptane/ethyl acetate 1:1 to afford compound ***N*-Boc-9** (55 mg, 44%). To ***N*-Boc-9** dissolved in dichloromethane and methanol, trifluoroacetic acid (10 mL/mmol) was added, and the reaction mixture stirred at r.t. for 2 h. Solvent and the volatiles were removed under reduced pressure to give **9*TFA (quant.)**, used without further purification. ^{1}H NMR (500 MHz, $CDCl_3$) δ 7.49 (dd, *J* = 8.5, 7.0 Hz, 1H), 7.09 (d, *J* = 7.0 Hz, 1H), 6.87 (d, *J* = 8.5 Hz, 1H), 6.22 (t, *J* = 5.6 Hz, 1H), 4.91 (dd, *J* = 12.4, 5.3 Hz, 1H), 3.26 (td, *J* = 7.0, 5.6 Hz, 2H), 3.12 (q, *J* = 7.0 Hz, 2H), 2.93 – 2.84 (m, 1H), 2.84 – 2.68 (m, 2H), 2.13 (ddt, *J* = 10.2, 5.2, 2.2 Hz, 1H), 1.67 (quint, *J* = 7.2 Hz, 2H), 1.53 – 1.47 (m, 2H), 1.44 (s, 11H), 1.40 – 1.34 (m, 2H). LCMS *m/z* $[M\text{-}TFA+Na]^+$ 495.2

2-(2-(2-aminoethoxy)ethoxy)ethyl (S)-2-(4-(4-chlorophenyl)-2,3,9-trimethyl-6H-thieno[3,2-f][1,2,4]triazolo[4,3-a][1,4]diazepin-6-yl)acetate hydrochloride (10*HCl). Following general procedure D, the desired compound was obtained from JQ1-carboxilic acid (50 mg, 0.09 mmol) and *tert*-butyl (3-hydroxypropyl)carbamate (29 mg, 0.11 mmol). The crude reaction mixture was purified by flash column chromatography using a gradient of 0% to 10% of methanol in dichloromethane to afford ***N*-Boc-10** as a colourless oil. To ***N*-Boc-10** dissolved in dichloromethane (10 ml/mmol), hydrogen chloride 4.0M in 1,4-dioxane (10 mL/1 mmol) was added, and the reaction mixture stirred at r.t. for 2 h. Solvent and the volatiles were removed under reduced pressure to give **10*HCl** (21 mg, 34%). ^{1}H NMR (500 MHz, $CDCl_3$) δ 7.50 (d, *J* = 8.1 Hz, 2H), 7.36 (d, *J* = 7.9 Hz, 2H), 4.80 (t, *J* = 7.0 Hz, 1H), 4.44 – 4.37 (m, 1H), 4.32 (dt, *J* = 12.3, 3.8 Hz, 1H), 3.89 – 3.57 (m, 12H), 2.95 (s, 3H), 2.44 (s, 3H), 1.68 (s, 3H). LCMS *m/z* $[M\text{-}Cl+H]^+$ 533.1

3-((*tert*-butoxycarbonyl)amino)propyl (S)-2-(4-(4-chlorophenyl)-2,3,9-trimethyl-6H-thieno[3,2-f][1,2,4]triazolo[4,3-a][1,4]diazepin-6-yl)acetate (*N*-Boc-11). Following general procedure D, the desired compound was obtained from JQ1-carboxilic acid (100 mg, 0.25 mmol) and *tert*-butyl (3-hydroxypropyl)carbamate (52 mg, 0.30 mmol). The crude reaction mixture was purified by flash column chromatography using a gradient of 0% to 10% of methanol in dichloromethane to afford **11** as a colourless oil (130 mg, 93%). To ***N*-Boc-11** dissolved in dichloromethane and methanol (10 ml/mmol), hydrogen chloride 4.0M in 1,4-dioxane (10 mL/1 mmol) was added, and the reaction mixture stirred at r.t. for 2 h. Solvent and the volatiles were removed under reduced pressure to give **11*HCl**, used without further characterization. ^{1}H NMR (500 MHz, $CDCl_3$) δ 7.34 (d, *J* = 8.5 Hz, 2H), 7.27 (d, *J* = 8.5 Hz, 2H), 4.54 (dd, *J* = 7.9, 6.3 Hz, 1H), 4.17 (h, *J* = 5.2 Hz, 2H), 3.61 (dd, *J* = 16.8, 6.3 Hz, 1H), 3.52 (dd, *J* = 16.8, 8.0 Hz, 1H), 3.15 (dt, *J* = 9.6, 4.9 Hz, 2H), 2.62 – 2.57 (s, 3H), 2.34 (s, 3H), 1.81 (quint, *J* = 6.3 Hz, 2H), 1.62 (s, 3H), 1.37 (s, 9H). LCMS *m/z* $[M+H]^+$ 559.2

1,1`-VH032-PEG3-ferrocene (AS1). Following general procedure C, the desired compound was obtained from 1,1'-ferrocenedicarboxylic acid (13 mg, 0.04 mmol) and **7c** (40 mg, 0.06 mmol) in dichloromethane (3 mL). Purification by normal phase flash column chromatography using a linear gradient of methanol in dichloromethane from 0 to 10% afforded **AS1** as light yellow oil (4.96 mg, 8%).^{1}H NMR (500 MHz, $CDCl_3$) δ 9.05 (d, *J* = 5.7 Hz, 2H), 8.61 (s, 2H), 7.51 – 7.43 (m, 4H), 7.39 (d, *J* = 8.1 Hz, 4H), 7.28 (d, *J* = 8.1 Hz, 4H), 4.83 (t, *J* = 8.3 Hz, 2H), 4.71 – 4.65 (m, 4H), 4.59 (dd, *J* = 15.3, 6.3 Hz, 2H), 4.44 (s, 2H), 4.38 (d, *J* = 7.0 Hz, 4H), 4.36 – 4.23 (m, 6H), 4.19 (s, 2H), 4.01 (d, *J* = 10.9 Hz, 2H), 3.81 – 3.71 (m, 4H), 3.64 (d, *J* = 15.7 Hz, 2H), 3.46 – 3.42 (m, 6H), 3.36 – 3.20 (m, 6H), 3.17 – 3.11 (m, 2H), 3.02 – 2.93 (m, 4H), 2.67 (d, *J* = 15.8 Hz, 2H), 2.58 (d, *J* = 10.1 Hz, 2H), 2.46 (s, 6H), 2.29 (dd, *J* = 12.9, 7.8 Hz, 2H), 2.18 (ddd, *J* = 13.2, 8.9, 4.6 Hz, 2H), 0.98 (s, 18H). HRMS (ESI) calculated for $C_{72}H_{97}FeN_{10}O_{16}S_2$ [M+H$^+$] 1477.5796; found 1477.5892.

1,1`-*cis*VH032-PEG3-ferrocene (*cis*AS1). Following general procedure C, the desired compound was obtained from 1,1'-ferrocenedicarboxylic acid (11 mg, 0.04 mmol) and amine **7b** (40 mg, 0.06 mmol) in dichloromethane (3.2 mL). Purification by normal phase flash column chromatography using a linear gradient of methanol in dichloromethane from 0 to 10% afforded ***cis*AS1** as light-yellow oil (6.2 mg, 9%). ^{1}H NMR (500 MHz, $CDCl_3$) δ 9.38 (dd, *J* = 6.9, 5.0 Hz, 2H), 8.68 (s, 2H), 7.60 (t, *J* = 5.4 Hz, 2H), 7.45 (d, *J* = 8.1 Hz, 4H), 7.36 (d, *J* = 8.1 Hz, 4H), 6.24 (d, *J* = 11.8 Hz, 2H), 5.30 (s, 2H), 4.99 – 4.88 (m, 4H), 4.78 (d, *J* = 10.2 Hz, 2H), 4.73 (s, 2H), 4.59 (s, 2H), 4.57 – 4.49 (m, 2H), 4.45 (s, 2H), 4.29 (t, *J* = 10.4 Hz, 2H), 4.26 – 4.22 (m, 2H), 4.20 (s, 2H), 4.10 (d, *J* = 10.9 Hz, 2H), 3.95 (dd, *J* = 11.1, 4.0 Hz, 2H), 3.84 (d, *J* = 1.1 Hz, 2H), 3.72 (d, *J* = 15.8 Hz, 2H), 3.57 (d, *J* = 10.5 Hz, 2H), 3.46 (dd, *J* = 16.3, 6.9 Hz, 4H), 3.34 (dd, *J* = 14.8, 7.0 Hz, 6H), 3.28 – 3.20 (m, 2H), 3.03 (t, *J* = 8.3 Hz, 2H), 2.94 (t, *J* = 8.0 Hz, 2H), 2.81 (d, *J* = 15.8 Hz, 2H), 2.53 (d, *J* = 13.0 Hz, 8H), 2.43 (ddd, *J* = 14.1, 9.6, 4.6 Hz, 2H), 2.30 (d, *J* = 14.1 Hz, 2H), 1.05 (s, 18H). HRMS (ESI) calculated for $C_{72}H_{97}FeN_{10}O_{16}S_2$ [M+H$^+$] 1477.5796, found 1477.5891.

1,1`-MeVH032-PEG3-ferrocene (AS2). Following general procedure C, the desired compound was obtained from 1,1'-ferrocenedicarboxylic acid (11 mg, 0.04 mmol) and **7c** (42 mg, 0.07 mmol) in dichloromethane (2.2 mL). Purification by normal phase flash column chromatography using a linear gradient of methanol in dichloromethane from 0 to 10% afforded **AS2** as pale-yellow oil (13 mg, 19%). ^{1}H NMR (500 MHz, $CDCl_3$) δ 8.60 (s, 2H), 8.38 (d, *J* = 7.3 Hz, 2H), 7.50 (t, *J* = 5.5 Hz, 2H), 7.44 (d, *J* = 10.0 Hz, 2H), 7.37 (d, *J* = 8.2 Hz, 4H), 7.31 (d, *J* = 8.3 Hz, 4H), 5.09 (quint, *J* = 6.9 Hz, 2H), 4.85 (t, *J* = 8.2 Hz, 2H), 4.71 – 4.63 (m, 4H), 4.49 (s, 2H), 4.40 (d, *J* = 15.7 Hz, 6H), 4.33 (t, *J* = 8.4 Hz, 2H), 4.22 (s, 2H), 4.00 (d, *J* = 11.2 Hz, 2H), 3.80 (s, 2H), 3.73 (d, *J* = 15.6 Hz, 2H), 3.66 (dd, *J* = 11.1, 3.2 Hz, 2H), 3.56 (d, *J* = 9.7 Hz, 6H), 3.40 (d, *J* = 8.1 Hz, 2H), 3.36 (d, *J* = 9.7 Hz, 2H), 3.30 (d, *J* = 10.0 Hz, 4H), 3.17 (s, 2H), 3.12 (s, 2H), 2.99 (d, *J* = 15.8 Hz, 2H), 2.45 (d, *J* = 7.6 Hz, 6H), 2.23 (dd, *J* = 13.2, 8.3 Hz, 2H), 2.12 (ddd, *J* = 8.1, 6.4, 3.5 Hz, 2H), 1.47 (d, *J* = 7.0 Hz, 6H), 0.96 (s, 18H). HRMS (ESI) calculated for $C_{74}H_{101}FeN_{10}O_{16}S_2$ [M+H$^+$] 1505.6109, found 1505.6179.

1`-PEG3-MeVH032-ferrocene-1-ethylamino-pomalidomide (AS3). Following general procedure A, the desired compound was obtained from **1** (20 mg, 0.02 mmol) and **8*TFA** (10 mg, 0.02 mmol) in dichloromethane. Purification by normal phase flash column chromatography using a linear gradient of methanol in dichloromethane from 0 to 5% afforded **AS3** as light-yellow oil (2.87 mg, 10 %). ^{1}H NMR (500 MHz, $CDCl_3$) δ 8.67 (s, 1H), 7.76 – 7.68 (m, 1H), 7.58 (dd, *J* = 10.1, 7.9 Hz, 1H), 7.50 (ddd, *J* = 8.6, 7.1, 3.2 Hz, 1H), 7.37 (d, *J* = 1.7 Hz, 4H), 7.30 (d, *J* = 9.1 Hz, 1H), 7.10 (dd, *J* = 7.2, 2.7 Hz, 1H), 7.05 (dd, *J* = 8.6, 1.6 Hz, 1H), 6.93 (s, 1H), 6.66 (d, *J* = 18.7 Hz, 1H), 5.10 (quint, *J* = 7.0 Hz, 1H), 4.93 (ddd, *J* = 17.2, 11.9, 5.6 Hz, 1H), 4.72 (td, *J* = 8.0, 3.4 Hz, 1H), 4.64 (dd, *J* = 9.2, 4.0 Hz, 1H), 4.59 (dddt, *J* = 5.2, 3.9, 2.7, 1.4 Hz, 2H), 4.56 – 4.53 (m, 1H), 4.53 – 4.49 (m, 2H), 4.38 – 4.33 (m, 2H), 4.33 – 4.28 (m, 1H), 4.05 (d, *J* = 11.4 Hz, 1H), 3.98 (dd, *J* = 15.5, 2.0 Hz, 1H), 3.80 (d, *J* = 5.8 Hz, 1H), 3.76 (dt, *J* = 10.9, 4.2 Hz, 2H), 3.72 – 3.54 (m, 12H), 3.52 (d, *J* = 6.9 Hz, 2H), 2.88 – 2.63 (m, 3H), 2.52 (s, 3H), 2.39 (dp, *J* = 13.3, 4.4 Hz, 1H), 2.12 (d, *J* = 46.7 Hz, 3H), 1.47 (dd, *J* = 7.1, 1.5 Hz, 3H), 1.08 – 0.98 (m, 9H). HRMS (ESI) calculated for $C_{58}H_{70}FeN_9O_{13}S$ 1188.4084 [M+H$^+$], found 1188.4152.

1`-PEG3-MeVH032-ferrocene-1-hexylamino-pomalidomide (AS4). Following general procedure A, the desired compound was obtained from **1** (22.0 mg, 0.02 mmol) and **9*TFA** (9.72 mg, 0.02 mmol) in dichloromethane. Purification by normal phase flash column chromatography using a linear gradient of methanol in dichloromethane from 0 to 5% afforded **AS4** as light-yellow oil (3.84 mg, 16 %). ^{1}H NMR (500 MHz, $CDCl_3$) δ 11.15 (s, 1H), 8.97 (d, *J* = 47.8 Hz, 1H), 8.60 (s, 1H), 7.60 (t, *J* = 8.6 Hz, 1H), 7.40 (dd, *J* = 8.5,

7.1 Hz, 1H), 7.31 (s, 5H), 7.00 (d, *J* = 7.1 Hz, 2H), 6.80 (d, *J* = 8.5 Hz, 1H), 6.16 (td, *J* = 5.6, 3.1 Hz, 1H), 5.04 (quint, *J* = 7.0 Hz, 1H), 4.85 (ddd, *J* = 12.4, 7.1, 5.3 Hz, 1H), 4.73 – 4.66 (m, 1H), 4.61 – 4.51 (m, 3H), 4.47 (dt, *J* = 2.7, 1.3 Hz, 1H), 4.45 (d, *J* = 2.6 Hz, 1H), 4.30 (q, *J* = 2.2 Hz, 1H), 4.27 (t, *J* = 2.5 Hz, 1H), 4.26 – 4.23 (m, 2H), 4.01 (dd, *J* = 11.4, 2.8 Hz, 1H), 3.98 – 3.92 (m, 1H), 3.76 – 3.68 (m, 2H), 3.60 – 3.42 (m, 12H), 3.34 – 3.26 (m, 2H), 3.20 (q, *J* = 6.5 Hz, 2H), 3.02 (d, *J* = 7.5 Hz, 1H), 2.82 – 2.61 (m, 3H), 2.45 (s, 3H), 2.34 (ddd, *J* = 13.0, 8.0, 4.6 Hz, 1H), 2.10 (dd, *J* = 13.6, 8.2 Hz, 1H), 2.07 – 2.01 (m, 1H), 1.60 (tdd, *J* = 13.7, 7.2, 3.6 Hz, 4H), 1.43 (dp, *J* = 23.4, 8.1 Hz, 8H), 0.98 (s, 9H). ^{13}C NMR (126 MHz, $CDCl_3$) δ 171.6, 171.2, 170.7, 170.5, 170.4, 170.2, 167.7, 169.7, 168.9,167.8, 150.4, 148.6, 147.1, 143.6, 136.3, 129.6 (2C), 126.6 (2C), 116.9, 111.6, 110.1, 78.5, 71.5, 71.5, 71.3, 71.1, 70.9, 70.8, 70.5, 70.3, 70.3, 70.1, 58.8, 57.1, 56.9, 49.1, 48.9, 42.7, 40.1, 39.9, 35.7, 31.6, 29.8, 29.1, 26.9, 26.6 (3C), 23.0, 22.5, 16.3. HRMS (ESI) calculated for $C_{62}H_{78}FeN_9O_{13}S$ [M+H$^+$]1244.4778, found 1244.4773.

1`-PEG3-MeVH032-ferrocene-1-ethylamino-pomalidomide (AS5). Following general procedure E, the desired compound was obtained from **3** (7 mg, 0.012 mmol) and **6c** (10 mg, 0.015 mmol). Purification by normal phase flash column chromatography using a linear gradient of methanol in dichloromethane from 0 to 5% afforded **AS5** as light-yellow oil (7.79 mg, 50 %). ^{1}H NMR (500 MHz, $CDCl_3$) δ 8.68 (s, 1H), 7.71 (d, *J* = 3.4 Hz, 1H), 7.53 – 7.43 (m, 2H), 7.41 – 7.33 (m, 4H), 7.29 (d, *J* = 8.9 Hz, 1H), 7.11 – 7.04 (m, 2H), 6.61 – 6.53 (m, 1H), 5.10 (dd, *J* = 13.9, 6.8 Hz, 1H), 4.90 (ddd, *J* = 20.0, 12.0, 5.8 Hz, 1H), 4.73 (td, *J* = 8.0, 3.2 Hz, 1H), 4.60 (t, *J* = 8.8 Hz, 1H), 4.56 – 4.50 (m, 4H), 4.50 – 4.42 (m, 2H), 4.35 – 4.30 (m, 2H), 4.28 (td, *J* = 2.6, 1.5 Hz, 2H), 4.11 (m, 2H), 4.00 (dd, *J* = 15.6, 8.1 Hz, 1H), 3.94 (d, *J* = 1.8 Hz, 1H), 3.92 – 3.86 (m, 2H), 3.67 – 3.51 (m, 12H), 3.49 (d, *J* = 5.2 Hz, 2H), 3.35 (s, 1H), 2.87 – 2.66 (m, 3H), 2.52 (d, *J* = 1.5 Hz, 3H), 2.47 (ddt, *J* = 12.6, 7.9, 4.4 Hz, 1H), 2.16 – 2.02 (m, 2H), 1.46 (d, *J* = 6.9 Hz, 3H), 1.04 (d, *J* = 2.8 Hz, 9H). ^{13}C NMR (126 MHz, $CDCl_3$) δ 171.9, 171.8, 171.3, 170.9, 170.4, 170.0, 169.0, 168.9, 167.7, 150.2, 146.8, 143.3, 136.2, 132.5, 130.8, 129.5, 126.5 (4C), 121.46, 117.1, 111.7, 110.2, 78.1, 76.5, 70.9, 70.9, 70.8, 70.6, 70.5, 70.4, 70.4, 70.2, 69.9, 69.9, 69.8, 69.5, 69.3, 69.2, 58.6, 56.9, 50.3, 48.8, 42.1, 38.9, 36.0, 35.4, 35.4, 31.4, 26.5 (3C), 22.8, 22.2, 16.1. HRMS (ESI) calculated for $C_{59}H_{70}FeN_{11}O_{12}S$ [M+H$^+$] 1212.4270, found 1212.4718.

1`-PEG3-MeVH032-ferrocene-1-PEG3-JQ1 (AS6). Following general procedure E, the desired compound was obtained from **4** (22 mg, 0.02 mmol) and **6c** (18 mg, 0.02 mmol). Purification by normal phase flash column chromatography using a linear gradient of methanol in dichloromethane from 0 to 10% afforded **AS6** as colourless oil (13.3 mg, 32 %). ^{1}H NMR (500 MHz, $CDCl_3$) δ 8.66 (s, 1H), 7.82 (d, *J* = 1.0 Hz, 1H), 7.65 – 7.55 (m, 1H), 7.43 – 7.34 (m, 6H), 7.33 – 7.28 (m, 3H), 6.82 (q, *J* = 5.6 Hz, 1H), 5.10 (quint, *J* = 7.1 Hz, 1H), 4.78 (t, *J* = 8.0 Hz, 1H), 4.68 (dd, *J* = 2.7, 1.3 Hz, 1H), 4.65 – 4.47 (m, 9H), 4.36 – 4.28 (m, 4H), 4.28 – 4.22 (m, 2H), 4.08 (dd, *J* = 11.4, 2.0 Hz, 1H), 3.98 (dd, *J* = 15.6, 3.9 Hz, 1H), 3.89 (dt, *J* = 11.9, 4.4 Hz, 3H), 3.74 (t, *J* = 4.9 Hz, 2H), 3.71 – 3.53 (m, 18H), 3.52 – 3.39 (m, 3H), 2.65 (d, *J* = 1.4 Hz, 3H), 2.52 (d, *J* = 1.9 Hz, 3H), 2.45 (ddd, *J* = 12.9, 8.0, 4.5 Hz, 1H), 2.40 (s, 3H), 2.18 (dddt, *J* = 11.6, 8.0, 3.7, 1.8 Hz, 1H), 1.47 (dd, *J* = 7.0, 3.3 Hz, 3H), 1.05 (s, 9H). ^{13}C NMR (126 MHz, $CDCl_3$) δ 171.7, 171.4, 170.2, 170.1, 169.9, 164.0, 155.4, 150.4, 150.0, 148.6, 145.0, 143.6, 136.9, 136.7, 132.4, 132.3, 131.7, 131.8, 131.0, 131.0, 130.9, 130.5, 130.0 (2C), 129.7 (2C), 128.8 (2C), 126.6 (2C), 121.6, 77.8, 77.4, 77.4, 76.9, 71.6, 71.1, 70.7, 70.6, 70.6, 70.5, 70.3, 70.3, 70.2, 70.1, 70.0, 69.6, 69.2, 68.8, 68.6, 64.1, 58.7, 57.1, 53.9, 50.4, 48.9, 39.4, 36.9, 36.3, 35.7, 26.7 (3C), 22.4, 16.3, 14.6, 13.3, 12.0. HRMS (ESI) calculated for $C_{69}H_{83}ClFeN_{12}O_{12}S_2$ [M+H$^+$] 1427.4727, found 1427.5375.

1`-PEG3-MeVH032-ferrocene-1-propylamino-JQ1 (AS7). Following general procedure E, the desired compound was obtained from **5** (4.7 mg, 0.007 mmol) and **6c** (5.0 mg, 0.007 mmol). Purification by normal phase flash column chromatography using a linear gradient of methanol in dichloromethane from 0 to 10% afforded **AS7** as colourless oil (3.6 mg, 37 %). ^{1}H NMR (500 MHz, $CDCl_3$) δ 8.67 (s, 1H), 7.80 (s, 1H), 7.55 (d, *J* = 7.8 Hz, 1H), 7.43 – 7.39 (m, 2H), 7.38 (d, *J* = 2.1 Hz, 4H), 7.34 – 7.30 (m, 3H), 7.22 (t, *J* = 5.8 Hz, 1H), 5.10 (quint, *J* = 7.0 Hz, 1H), 4.77 (t, *J* = 8.0 Hz, 1H), 4.67 – 4.60 (m, 3H), 4.55 (dddd, *J* = 15.5, 9.1, 2.5, 1.3 Hz, 6H), 4.38 – 4.28 (m, 4H), 4.27 – 4.18 (m, 4H), 4.09 (d, *J* = 11.2 Hz, 1H), 3.99 (d, *J* = 15.6 Hz, 1H), 3.92 – 3.88 (m, 3H), 3.71 (dd, *J* = 16.6, 7.2 Hz, 1H), 3.67 – 3.52 (m, 11H), 3.40 (dq, *J* = 12.8, 6.4 Hz, 1H), 3.31 (dq, *J* = 13.0, 6.4 Hz, 1H), 2.67 (s, 3H), 2.52 (s, 3H), 2.46 (ddd, *J* = 13.0, 8.0, 4.6 Hz, 1H), 2.42 – 2.38 (m, 3H), 2.25 – 2.16 (m, 1H), 1.96 (h, *J* = 7.4 Hz, 2H), 1.48 (d, *J* = 7.0 Hz, 4H), 1.05 (s, 9H). HRMS (ESI) calculated for $C_{66}H_{78}ClFeN_{12}O_{10}S_2$ [M+H$^+$] 1353.4437, found 1353.4835.

NMR studies.

Conformational analysis was conducted on a sample with a concentration of 1.25 mM of the FerroTACs. Standard NMR experiments were performed at 298 K, while variable-temperature NMR experiments recorded spectra across a temperature range from 298 K to 328 K allowing a sufficient temperature equilibration time (10–15 min) before signal acquisition. All NOESY spectra for conformational analysis as well as the assignment spectra in methanol-d_4 were recorded on a 500 MHz Bruker Avance III equipped with a 5 mm QCI cryogenic probe. NOESY spectra were obtained from a sample at 1.25 mM in $CDCl_3$ and 1.25 mM in methanol-d_4, recorded at 298 K, using 16 transients, 1024 points in the direct dimension and 512 points in the indirect dimension, a relaxation delay of 1.5 s, and a mixing time of 700 ms. All other spectra used for signal assignment in $CDCl_3$ were recorded on 500 MHz Bruker Avance NEO with a BBFO SmartProbe. Chemical shifts (δ/ppm) were referenced to the residual solvent peak in ^{1}H (7.26 ppm for $CDCl_3$, 3.31 ppm for CD_3OD. 2.50 ppm for *d*-DMSO) and ^{13}C spectra (77.16 ppm for $CDCl_3$, 49.00 ppm for CD_3OD, 39.52 ppm for DMSO-*d6*). Coupling constants (*J*) are given in Hz. Signal splitting patterns are described as singlet (s), doublet (d), triplet (t), quartet (q), quintet (quint), multiplet (m), broad (br) or a combination thereof.

Biology.

Cell Culture

HEK293 and HCT116 cell lines, purchased from ATCC, were cultured in Dulbecco's modified Eagle's medium (DMEM, Gibco) supplemented with 10% fetal bovine serum (FBS, Gibco), L-glutamine (2 mM, Gibco) and 100 µg/mL of penicillin/streptomycin (Gibco). All cell lines were maintained in a humidified incubator at 37 °C and 5% CO_2 for no more than 30 passages. Cells were routinely tested for mycoplasma contamination.

Cell Treatment for Immunoblotting

HEK 293 cells were plated in 6-well plates at varying densities (4-5 × 10^5 cells/mL) 24-42 h before treatment depending on the experimental setup. Cells were treated in fresh medium with the indicated compounds under indicated conditions with a final EtOH or iPrOH concentration of 0.01% (v/v) to 0.1% (v/v) depending on the experimental setup. After compound treatment, the medium was removed, and the cells were washed with ice-cold Phosphate-Buffered Saline (PBS) and lysed on ice with 100 µL RIPA lysis and extraction buffer (Thermo Fisher Scientific, #89900) supplemented with complete EDTA-free protease inhibitor cocktail (11873580001,Roche). Cells were incubated for 15 min on ice and then detached from the surface by scraping. After removal of the insoluble fraction by centrifugation at 15,000 g at 4 °C for 15 min, supernatants were stored at −80°C. Protein concentration was determined by bicinchoninic acid (BCA) assay (Thermo Fisher Scientific, #23225) For the mechanistic co-treatments, cells were treated with **AS2** for 4 h at final concentration of 1 µM and 0.2% EtOH. Prior to the treatment with **AS2**, wells were treated with either 3 µM MLN4924 (Sigma-Aldrich, #5.05477) for 3.5 h, with a final concentration of 25 µM MG132 (Sigma-Aldrich·, #474790) for 30 min or with a final concentration of 50 µM **VH032** for 30 min.

Quantitative Immunoblotting

Cell lysates containing a quarter of a volume of 4× NuPAGE LDS sample buffer (NP0007) supplemented with 10% β-mercaptoethanol or DTT were heated at 95 °C for 5 min. Samples (20 to 30 µg) were loaded onto precast 4 – 12% bis–tris midi 20W or 26W gels (Thermo Fisher Scientific) and resolved at 90 V for 10 min and then at 130 V for 1.5 h with a NuPAGE MOPS SDS running buffer (Thermo Fisher Scientific). Proteins were electrophoretically transferred onto a 0.45 µm nitrocellulose membrane (GE Healthcare, Amersham Protran Supported 0.4 mm NC) at 90 V for 90 min on ice in a transfer buffer (25 mM tris base and 192 mM glycine supplemented with 20% ethanol). The transferred membrane was blocked with 5% (w/v) skim milk powder dissolved in tris-buffered saline with Tween (TBS-T) (50 mM tris base, 150 mM sodium chloride (NaCl), 0.1% (v/v) Tween-20) at room temperature for 1 h. Western blot images were obtained through detection with anti-VHL (Cell Signaling Technology, #68547; 1:1000), anti-HIF-1α (BD Biosciences, #610959, clone 54, 1:1,000) and anti-hydroxy-HIF-1α (Pro564) (Cell Signaling Technology; #3434, 1:1,000) anti-CRBN (abcam, #ab244223, 1:1,000), anti-BRD4 (Cell Signaling Technology; #E2A7X, 1:1,000), anti-BRD3 (abcam, #ab50818, 1:1,000), anti-BRD2 (abcam, #ab243865, 1:1,000) antibodies.

Following overnight incubation with the primary antibodies at 4 °C, the membranes were washed two times for 10 min with TBS-T and then,incubated with secondary antibodies IRDye 800CW donkey anti-rabbit secondary

antibody (LI-COR #926-32213, 1:5,000) or IRDye 800CW donkey anti-mouse secondary antibody, (Li-COR #926-32212, 1:5,000) and hFABTM rhodamine anti-tubulin antibody (Biorad, 12004165, 1:10,000) for 1 h at room temperature and protected from light. Thereafter, the membranes were washed with TBS-T three times for 10 min, and protein bands were acquired using a ChemiDoc MP imaging system (Bio-Rad). Band quantification was performed using Image Lab software and reported as ratio of each protein band relative to the lane's loading control. The values obtained were then normalised to vehicle control.

Native Gel Electrophoresis Assay

Gel casting. Continuous 10% polyacrylamide gels were prepared from 3.75 mL 40% acrylamide solution, 7.5 mL ddH_2O, 3.75 mL 1.5 M Tris buffer at pH = 8.8, 150 µL of 10% (*w/v*) aqueous ammonium persulfate solution and 10 µL tetramethyl ethylenediamine and were polymerised in washed, SDS-free gel casting system for 1 h and used within one week.

PROTACs screen for ternary complex formation. VCB recombinant protein was diluted in sample buffer (20 mM HEPES, 150 mM NaCl, pH adjusted to pH = 7.0) to a final sample concentration of 12.11 µM and 0.5 µL of 20X EtOH stocks of the respective PROTACs were added (final concentration 24.22 µM) and were incubated for 30 min at room temperature. 1 µL of native gel dye (made from 2.5 mL tris-glycine running buffer, 5 mL glycerol, 1 mL bromophenol blue (1%) and 1.5 mL ddH_2O) were added, and the samples were loaded into the native gel and resolved at 100 V for at least 120 min on ice in a tris-glycine running buffer (25 mM tris base and 192 mM glycine). Protein bands were visualised with Instantblue Coomassie stain (abcam, #ab119211).

*Competition experiment of **AS2** with **VH298**.* The VCB recombinant protein was diluted in sample buffer (20 mM HEPES, 150 mM NaCl, pH adjusted to 7.0) to a final concentration of 12.11 µM. To this, 0.25 µL of 40X DMSO stock solution of **VH298** was added at varying ratios of 1:1, 1:1.5, 1:2, 1:4, 1:8, 1:10, 1:20. Following a 15-minute incubation at room temperature, 0.25 µL of a 40X ethanol stock solution of **AS2** (final concentration 24.22 µM) was added, and incubation continued for another 30 minutes at room temperature. Then, 1 µL of native gel dye was added, and the samples were loaded onto a native gel and resolved at 100 V for at least 120 minutes on ice in Tris-glycine running buffer (25 mM Tris base and 192 mM glycine). Protein bands were visualised using InstantBlue Coomassie stain (abcam, #ab119211). Similarly, the VCB recombinant protein was diluted in sample buffer (20 mM HEPES, 150 mM NaCl, pH adjusted to 7.0) to a final concentration of 2.42 µM, and 0.25 µL of 40X DMSO stock solution of **VH298** was added to achieve a final concentration of 3.63 µM. After 15 minutes of incubation at room temperature, 0.25 µL of 40X ethanol stock solution of **AS2** was added at increasing ratios of 1:0.2, 1:0.5, 1:1, 1:2, 1:10, 1:20, and 1:40 to VCB. The incubation was continued for another 30 minutes at room temperature. The gel was processed as described above.

Analytical Size Exclusion Chromatography (SEC)

SEC experiments were carried out in a ÄKTA pure system (GE Healthcare) at room temperature. VCB protein (10 µM) was incubated in buffer (20 mM HEPES,150 mM NaCl, pH = 7.0) with **CM11** (6 µM), **VH298** (12 µM) or ethanol (5%) for 15 min on ice or with **AS2** (20 µM or 6 µM) and ***cis*AS1** (20 µM) for 30 min at room temperature in 250 µL sample volume each prior to injection. Samples were run on a Superdex 200 Increase 10/300 GL column (GE Healthcare) at a flow rate of 0.5 mL · min^{-1} in 20 mM HEPES and 150 mM NaCl, pH = 7.0. Peak elution was monitored using ultraviolet absorbance at 280 nm.

Cell Viability Assays

Cells (HEK293 and HCT116) were seeded in 96-well plates at a density of 40,000 cells per well and treated for 72 hours with iPrOH or test compounds across eight 1/3 serially diluted concentrations, starting from 10 µM. Each treatment was performed in biological duplicates, with technical duplicates for each. Cell viability was measured using the CellTiter-Glo assay (CellTiter-Glo Luminescent Cell Viability Assay, Promega G7573) following the manufacturer's protocol. Luminescence was recorded on a GloMax Discover luminometer (Promega). Survival curves and EC_{50} values were generated using GraphPad Prism v.10.0.3 by fitting a nonlinear regression, with each data point normalised to the average luminescence of the corresponding control vehicle concentration.

Lytic HiBit-BET Degradation Assay

Cells were seeded at a density of 10,000 cells per well in DMEM with 10% FBS in white 96-well tissue culture plates and left to adhere overnight. iPrOH or test compounds across twelve 1/3 serially diluted concentrations, with an initial concentration of 10 μM for all compounds. Each treatment was conducted in biological duplicates with technical duplicates for each. After a 6-hour incubation, 100 μL of Promega Nano-Glo HiBiT lytic detection reagent mix (Promega Nano-Glo HiBiT Lytic Detection System #N3050), prepared according to the manufacturer's instructions, was added to each well. Plates were placed on an orbital shaker for 15 minutes to ensure complete cell lysis. Luminescence was recorded using the GloMax® Ultrasensitive Luminescence Protocol for 96-well plates. Luminescence readings were normalised against the mean values from iPrOH-treated controls and expressed as a ratio of the vehicle control. Curve fitting and DC_{50} values were calculated in GraphPad Prism v.10.0.3 using nonlinear regression. DC_{50} values were obtained *via* a three-parameter logistic model, and D_{max} values indicate the highest degradation achieved, expressed as a percentage of control treatments.

Live Kinetics HiBiT-BET Degradation Assay

To perform the transient transfection of HEK293 cells with HiBiT-tagged BRD4, BRD3, or BRD2 using LgBiT, lipid complexes were prepared. For each transfection, 1 μg of LgBiT DNA was mixed with 4 μg of carrier DNA in 100 μL of Opti-MEM without phenol red. Then, 6 μL of FuGENE® HD was added per mL of the DNA mixture. The mixture was gently inverted 5-10 times and incubated at room temperature for 20 minutes to form lipid complexes. Meanwhile, HEK293 cells were prepared at a density of 2 x 10^5 cells/mL. For each well of a 6-well plate, 2 mL of the cell suspension was seeded. After seeding, 100 μL of the lipid complex was added dropwise to each well for even distribution. The cells were incubated at 37 °C with 5% CO_2 for at least 20 hours to promote protein expression. On the second day, the transfected cells were tryptinised and collected into a single suspension. The total cell count was adjusted to 5 x 10^5 cells/mL, and 100 μL of this suspension was transferred to each well of a 96-well plate, including some negative control wells without transfected cells. The 96-well plate was incubated at 37 °C with 5% CO_2 for 24 hours. On the third day, preparations for the degradation assay began 2.5 hours before measurement by incubating the cells with Endurazine (Promega kit). The Endurazine substrate was diluted from 100x to 1x in cell culture media. The overnight media was removed, and 90 μL of the diluted Endurazine was added to each well. The cells were incubated for another 2.5 hours at 37 °C with 5% CO_2. A 10X PROTAC and control solutions were prepared in cell culture media. After luminescence equilibration, a pre-read luminescence measurement was taken to normalise for baseline differences. Finally, 10 μL of these solutions was dispensed in triplicate at final concentrations of 1 μM, 0.1 μM, and 0.01 μM. The treated plates were placed in a GloMax Discover luminometer (Promega) pre-warmed to 37 °C, and luminescence measurements were obtained every 7 minutes for 21 hours. Kinetic degradation plots were created by normalising luminescence readings in each well to the pre-read measurement and then to the average value of the iPrOH-only control at each time point.

NanoBRET live *vs* lytic target engagement.

VHL (N2930, Promega) NanoBRET target engagement assays were conducted following the manufacturer's guidelines. Transfection complexes were prepared by mixing 1 mL of Opti-MEM, 30 μL of FuGENE® HD, 9 μg/mL Transfection Carrier DNA, and 1 μg/mL of the VHL-NanoLuc fusion vector. These complexes were allowed to form over 20 minutes at room temperature, then added to 20 mL of HEK293 cells at a density of 200,000 cells/mL. The transfected HEK293 cells were then plated in a T75 flask and left to express the protein overnight.

The following day, 75 μL of transfected cells (at 200,000 cells/mL) were plated in white, non-binding 96-well plates for live and lytic target engagement assays. PROTACs and vehicle controls were prepared at 10x their final concentrations in Opti-MEM.

For the live-cell assay,10 μL of additional media was added to each well, followed by 10 μL of each 10x PROTAC and vehicle control dilution. A 100x solution of NanoBRET tracer (100 μM for VHL live-cell assay) was prepared in 100% DMSO and then diluted to a 20x solution using tracer dilution buffer. A final volume of 5 μL of the tracer was added to each well at a final concentration of 1 μM. Plates were mixed on an orbital shaker at 300 RPM for 15 seconds and then incubated at 37 °C with 5% CO_2 for 4 hours. Following incubation, 50 μL of a 3x substrate solution (8.8 μL NanoBRET Nano-Glo substrate, 7 μL Extracellular NanoLuc inhibitor, and 3500 μL Opti-MEM) was added to each well. After 2-3 minutes of incubation at room temperature, donor (450 nm) and acceptor (610 nm) emissions were measured using a GloMax Discover luminometer (Promega)

For the permeabilised-cell assay, 10 µL of digitonin was added, and plates were incubated in darkness for 5 minutes to allow permeabilisation. In each well 10 µL of the 10x PROTAC and vehicle control dilutions were dispensed. A 100x NanoBRET tracer solution (50 µM for VHL permeabilised-cell assay) was prepared with 100% DMSO, then diluted to a 20x solution with tracer dilution buffer. A 5 µL volume of tracer was dispensed into each well at a final concentration of 0.5 µM, and the plate was mixed at 300 RPM for 15 seconds. Finally, 50 µL of a 3x substrate solution (8.8 µL NanoBRET Nano-Glo substrate and 3,500 µL Opti-MEM) was added, and plates were incubated for 1 minute at room temperature before reading on a GloMax Discover as described above.

HiBiT Lytic Assay with Zosuquidar Co-treatment

A modified HiBiT lytic assay was used to assess the activity of zosuquidar on the BET degradation. Cells were seeded in 80 µL as previously described and incubated for 1 hour with a 10X stock of zosuquidar (Selleckchem, #S1481) or DMSO at a final concentration of 500 nM. For testing, iPrOH or various test compounds were applied at eight 1/3 serially diluted concentrations, beginning at an initial concentration of 10 µM for each. Each condition was tested in biological triplicate with technical duplicates. After a 4.5-hour incubation, 10 µL of 10X CellTiter-Fluor™ reagent (Promega) was added to each well at a final concentration of 50 nM, followed by a 30-minute incubation. Next, 100 µL of Promega Nano-Glo HiBiT lytic detection reagent (Nano-Glo HiBiT Lytic Detection System #N3050) was prepared per manufacturer instructions and added to each well. Plates were then placed on an orbital shaker for 15 minutes to ensure complete cell lysis. Luminescence was measured using the GloMax® Multiplex Luminescence and Fluorescence Protocol for 96-well plates. The luminescence signal for each well was normalised to the fluorescence signal and further normalised to the mean values from iPrOH-treated controls, then expressed as a ratio relative to the vehicle control. Curve fitting and DC_{50} values were determined in GraphPad Prism v.10.0.3 using nonlinear regression with a three-parameter logistic model to calculate DC_{50} values.

Chrom LogD Experimental

The stock solution of each test item was prepared from a 10mM stock in iPrOH, which was further diluted 1:50 with MeOH to obtain a 0.2 mM solution (4 µL of the test item stock solution is diluted with 196 µL DMSO) in insert vials. The samples were analysed on an HPLC system equipped with a HPLC-ThermoScientific Hypersil GOLD-C18 column (2.1 x 50 mm, 1.9 µm) and by MS detection using electrospray ionization (ESI) and Atmospheric Pressure Chemical Ionization (APCI) on a Shimadzu LCMS 2050. A linear 2.50 min gradient from 5 to 95% of organic phase (20 mM ammonium acetate in 95% acetonitrile pH 7.4) was applied with a flow rate of 1 mL/min and a column temperature of 40 °C. Reagents for the preparation of mobile phases were as follows: LiChropur ammonium acetate (Merck, purity grade ≥98%, product number 73594), ammonium hydroxide (Acros Organic, purity grade 28–30% in water), acetonitrile LC–MS grade, and ultrapure water prepared with Milli-Q Gradient (Q-POD – LC-Pak). Data were analysed with Shimadzu Open Solution Data Browser to obtain the RTs of the peak for each individual compound and standards. The RTs were next imported to establish a linear regression curve for the standards, comparing RTs with known Log$D_{7.4}$ values. ChromLog*D* values for the compounds were then calculated based on the regression curve.

Pharmacokinetic (PK) Study

The following *in vitro* PK profiling was outsourced and performed by Shanghai ChemPartner Co., Ltd.

Log$D_{7.4}$ Calculation

10 µL of 10 mM stock solution was added to a 96-well plate, followed by 300 µL of octanol. The plate was sealed, agitated on a shaker (5 min), and centrifuged (2000 rpm, 5 min). After removing the seal, 600 µL of potassium phosphate buffer (pH 7.4) was added. The plate was resealed, mixed (1 h, 25 °C), and centrifuged again (2000 rpm, 5 min). For the water phase, 10 µL of sample was diluted 1:2 with water, followed by a 1:20 dilution with 50% ethanol. 50 µL of the sample with 100 µL of 50% ethanol containing internal standard. For the octanol phase, 10 µL of the upper phase was diluted with 190 µL of 50% ethanol. This was further diluted by transferring 10 µL to a deep-well plate with 390 µL of ethanol. A final 1:2400 dilution was prepared by adding 100 µL of ethanol with IS to 50 µL of the 1:800 diluted sample. Samples were prepared for LC/MS analysis.

Solubility in Phosphate Buffer and Fed State Simulated Intestinal Fluid (FeSSIF)
8 µL of reference or test compound stock solution (10 mM in iPrOH) was added to 792 µL of 100 mM phosphate buffer (pH 7.4) or FeSSIF (pH 5.8). The resulting mixture was shaken for 1 h (1000 rpm) at room temperature and then centrifuged for 10 min (12 000 rpm) to remove the undissolved particles. The supernatant was collected and diluted 10 times and 100 times separately with 100 mM phosphate buffer or FeSSIF. Five microliters of the supernatant samples (no diluted, 10 times diluted, 100 times diluted) were mixed with 95 µL of acetonitrile (containing internal standard) separately before injecting into LC-MS/MS for analysis.

Plasma Stability Assay
Frozen plasma was thawed at 37 °C and centrifuged at 3000 rpm for 8 min to remove clots, and the supernatant was used in the experiment. The pH of the plasma was recorded, and only the pH range between 7.4 and 8 was used. The plasma and compound solution were prewarmed to 37 °C. 10 µL of prewarmed testing compound or reference compound (procaine) solution (20 µM in 0.05 mM sodium phosphate buffer (pH 7.4) with 0.5% BSA) was mixed with 90 µL of plasma at different time points to allow for 5, 15, 30, 45, and 60 min of incubation time. For 0 min, the plasma was mixed with vehicle only. Acetonitrile was added to the compound and plasma mixture to quench the reaction, and the resulting mixture was centrifuged (5594*g* for 15 min). The supernatant was taken and diluted before LC-MS analysis

Mouse Liver Microsome Stability
Testing compound (1.5 µL) or reference compound (500 µM in 5% iPrOH and 95% acetonitrile) was mixed with 18.75 µL of 20 mg/mL liver microsome (Corning) and 479.75 µL of potassium phosphate buffer (0.1 M potassium phosphate buffer, 1 mM EDTA, pH 7.4). The reaction was started by mixing 30 µL of the above mixture (prewarmed to 37 °C) with 15 µL of 6 mM NADPH stock solution (prewarmed to 37 °C). After incubating for 5, 15, 30, or 45 min, 135 µL of acetonitrile containing internal standard was added to stop the reaction. For 0 min, the compound and microsome mixture were mixed with acetonitrile first before adding NADPH. After quenching, the reaction mixture was centrifuged, and the supernatant was taken and diluted for LC-MS analysis.

HPLC/HRMS Traces for compounds AS1-AS7

AS1

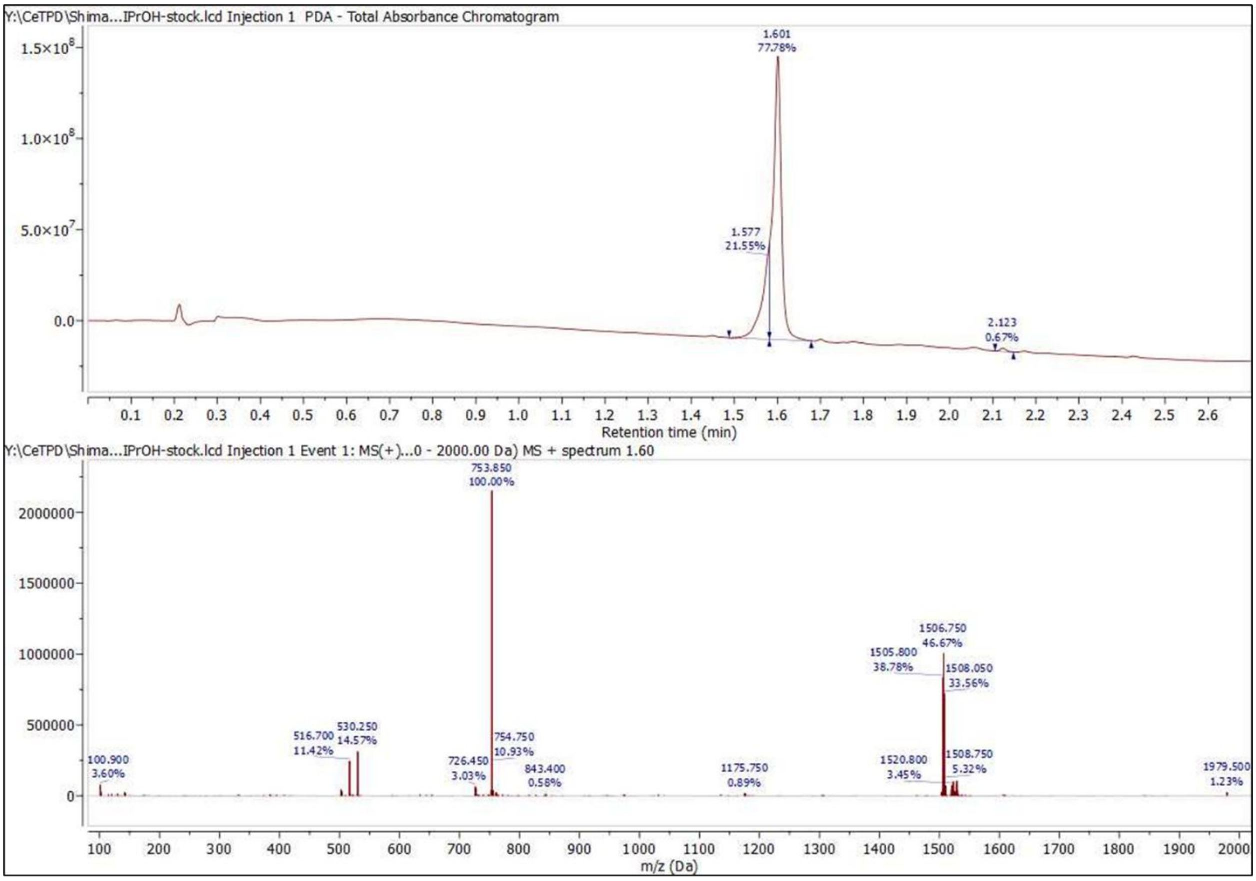

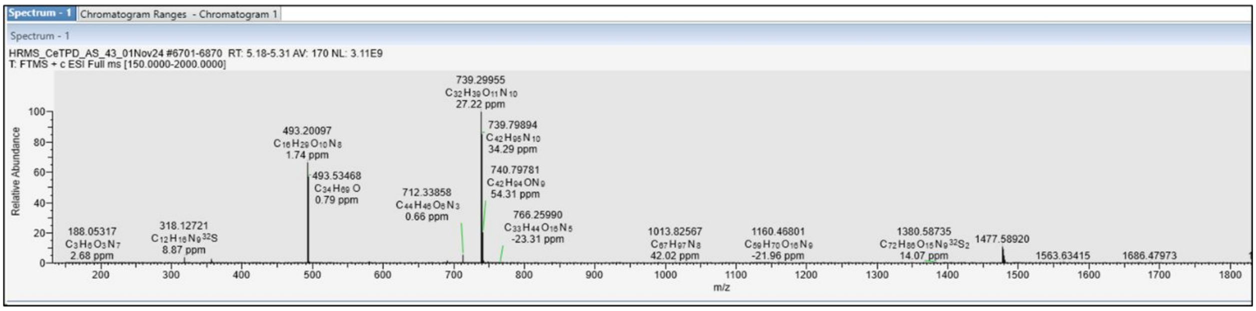

***cis*AS1**

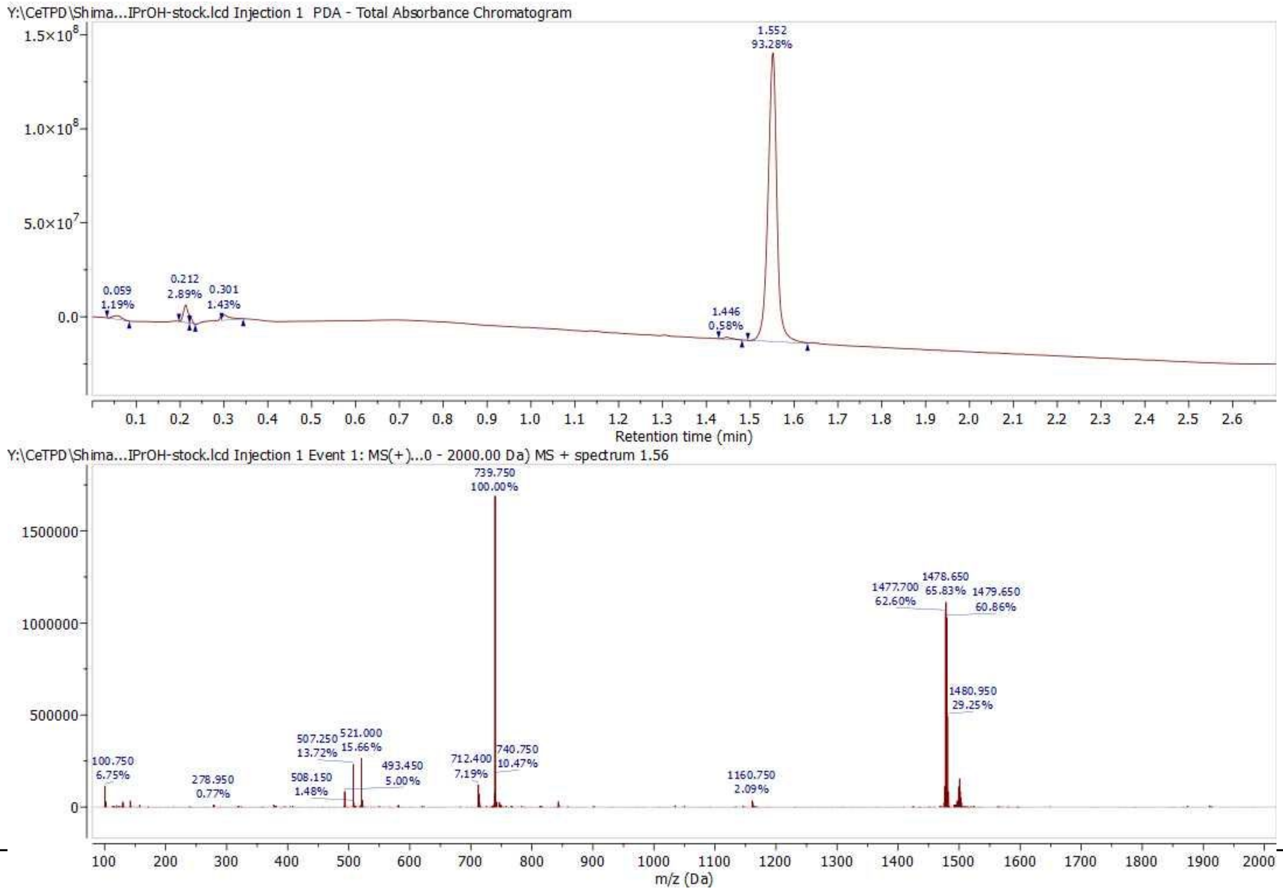

Y:\CeTPD\Shima...IPrOH-stock.lcd Injection 1 PDA - Total Absorbance Chromatogram
1.552
93.28%
0.059
1.19%
0.212
2.89%
0.301
1.43%
1.446
0.58%
Retention time (min)
Y:\CeTPD\Shima...IPrOH-stock.lcd Injection 1 Event 1: MS(+)...0 - 2000.00 Da) MS + spectrum 1.56
739.750
100.00%
1478.650
65.83%
1477.700
62.60%
1479.650
60.86%
1480.950
29.25%
507.250
13.72%
521.000
15.66%
493.450
5.00%
712.400
7.19%
740.750
10.47%
100.750
6.75%
278.950
0.77%
508.150
1.48%
1160.750
2.09%
m/z (Da)

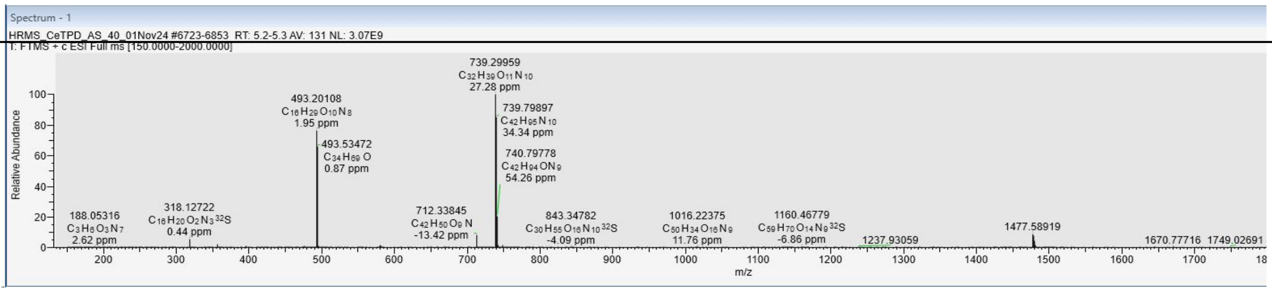

Spectrum - 1
HRMS_CeTPD_AS_40_01Nov24 #6723-6853 RT: 5.2-5.3 AV: 131 NL: 3.07E9
T: FTMS + c ESI Full ms [150.0000-2000.0000]
739.29959
C32 H39 O11 N10
27.28 ppm
493.20108
C16 H29 O10 N8
1.95 ppm
739.79897
C42 H95 N10
34.34 ppm
493.53472
C34 H69 O
0.87 ppm
740.79778
C42 H94 ON9
54.26 ppm
Relative Abundance
188.05316
C3 H8 O3 N7
2.62 ppm
318.12722
C16 H20 O2 N3 32S
0.44 ppm
712.33845
C42 H50 O9 N
-13.42 ppm
843.34782
C30 H55 O16 N10 32S
-4.09 ppm
1016.22375
C50 H34 O16 N9
11.76 ppm
1160.46779
C59 H70 O14 N9 32S
-6.86 ppm
1237.93059
1477.58919
1670.77716
1749.02691
m/z

AS2

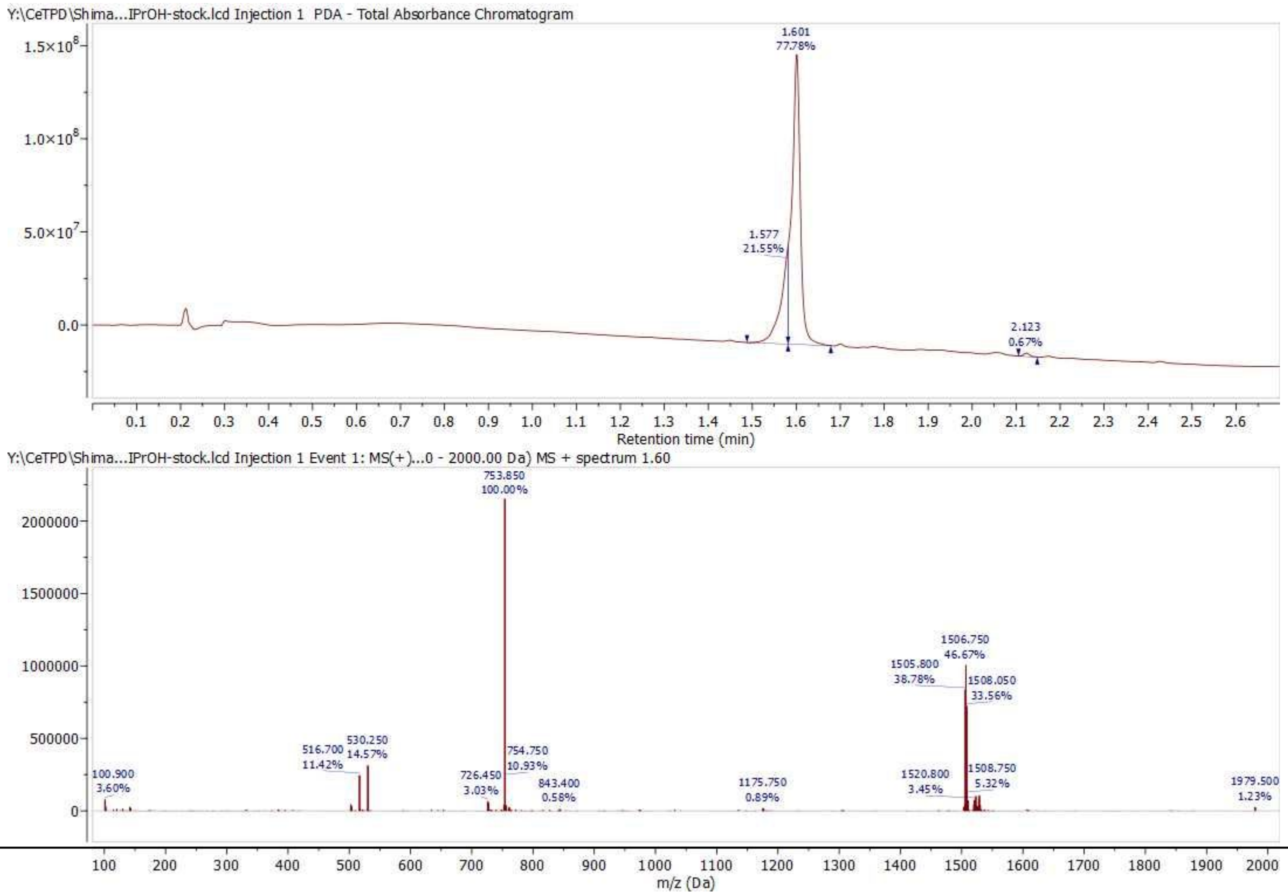
Y:\CeTPD\Shima...IPrOH-stock.lcd Injection 1 PDA - Total Absorbance Chromatogram
1.601
77.78%
1.577
21.55%
2.123
0.67%
Retention time (min)
Y:\CeTPD\Shima...IPrOH-stock.lcd Injection 1 Event 1: MS(+)...0 - 2000.00 Da) MS + spectrum 1.60
753.850
100.00%
1506.750
46.67%
1505.800
38.78%
1508.050
33.56%
516.700
11.42%
530.250
14.57%
754.750
10.93%
726.450
3.03%
843.400
0.58%
100.900
3.60%
1175.750
0.89%
1520.800
3.45%
1508.750
5.32%
1979.500
1.23%
m/z (Da)

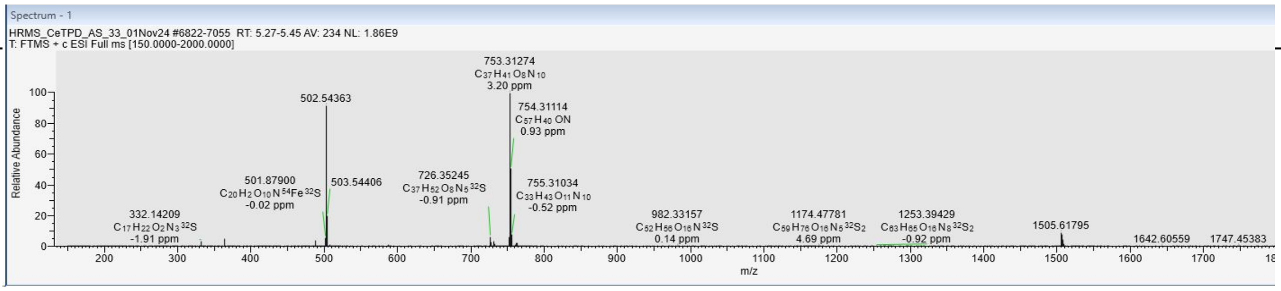
Spectrum - 1
HRMS_CeTPD_AS_33_01Nov24 #6822-7055 RT: 5.27-5.45 AV: 234 NL: 1.86E9
T: FTMS + c ESI Full ms [150.0000-2000.0000]
Relative Abundance
753.31274
C37 H41 O8 N10
3.20 ppm
502.54363
754.31114
C57 H40 ON
0.93 ppm
501.87900
C20 H2 O10 N 54Fe 32S
-0.02 ppm
503.54406
726.35245
C37 H52 O8 N5 32S
-0.91 ppm
755.31034
C33 H43 O11 N10
-0.52 ppm
332.14209
C17 H22 O2 N3 32S
-1.91 ppm
982.33157
C52 H56 O18 N 32S
0.14 ppm
1174.47781
C59 H76 O18 N8 32S2
4.69 ppm
1253.39429
C63 H65 O18 N8 32S2
-0.92 ppm
1505.61795
1642.60559
1747.45383
m/z

AS3

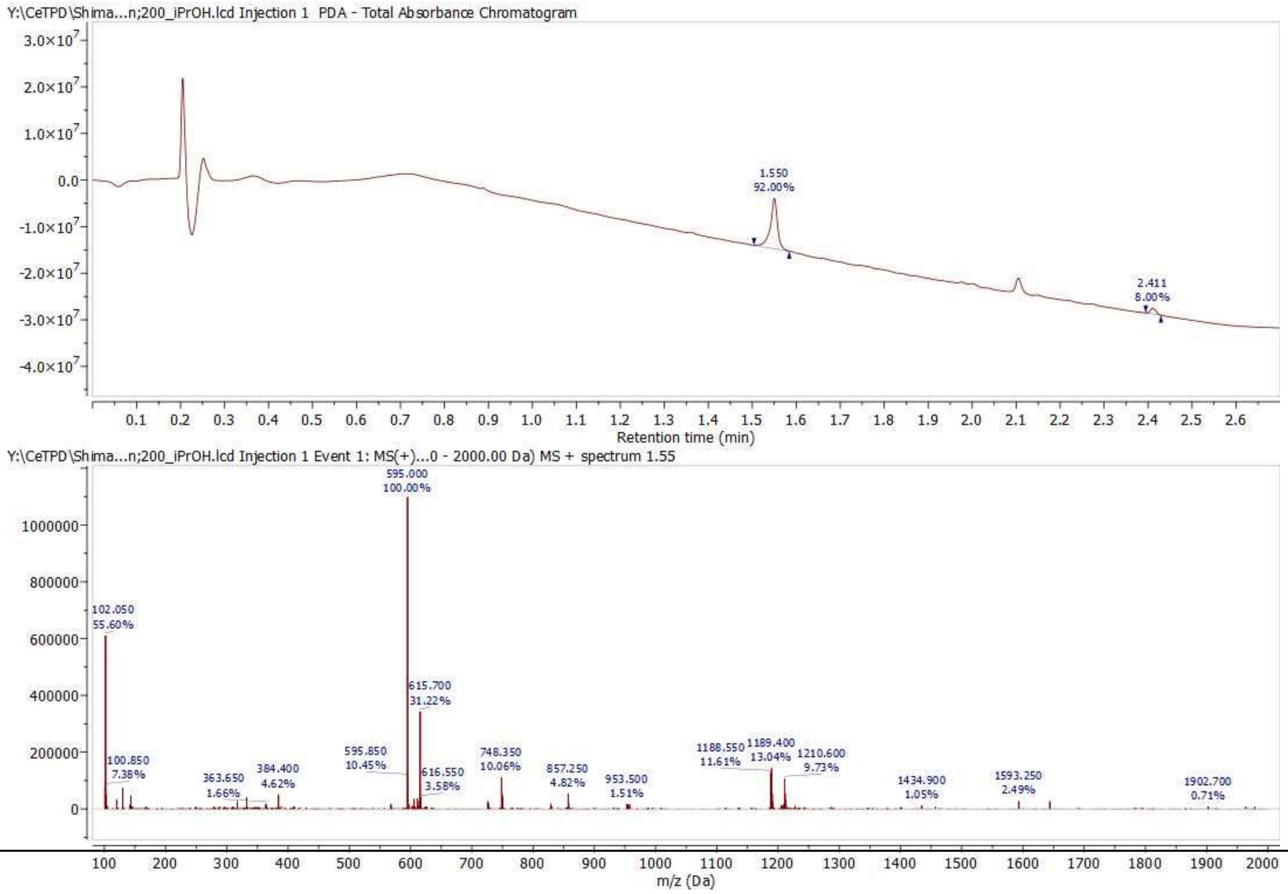

Y:\CeTPD\Shima...n;200_iPrOH.lcd Injection 1 PDA - Total Absorbance Chromatogram
1.550
92.00%
2.411
8.00%
Retention time (min)
Y:\CeTPD\Shima...n;200_iPrOH.lcd Injection 1 Event 1: MS(+)...0 - 2000.00 Da) MS + spectrum 1.55
595.000
100.00%
102.050
55.60%
615.700
31.22%
1189.400
13.04%
1188.550
11.61%
595.850
10.45%
748.350
10.06%
1210.600
9.73%
100.850
7.38%
857.250
4.82%
384.400
4.62%
616.550
3.58%
1593.250
2.49%
363.650
1.66%
953.500
1.51%
1434.900
1.05%
1902.700
0.71%
m/z (Da)

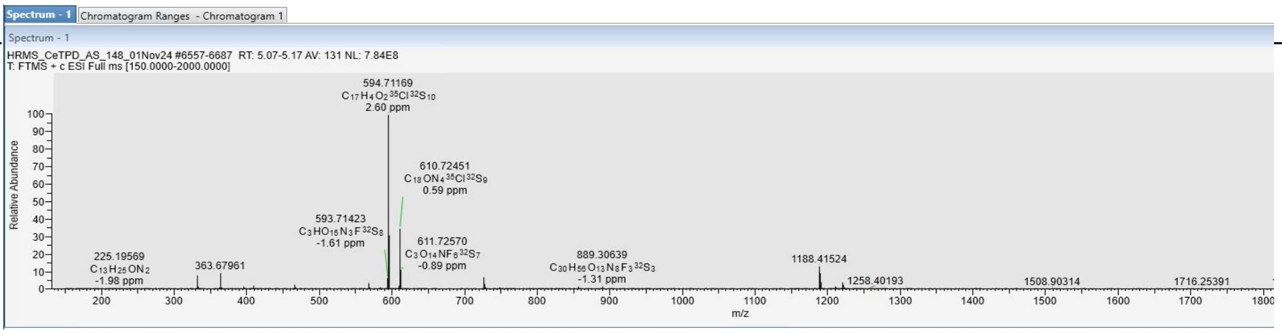

Spectrum - 1
Chromatogram Ranges - Chromatogram 1
HRMS_CeTPD_AS_148_01Nov24 #6557-6687 RT: 5.07-5.17 AV: 131 NL: 7.84E8
T: FTMS + c ESI Full ms [150.0000-2000.0000]
594.71169
C17 H4 O2 35Cl 32S10
2.60 ppm
610.72451
C18 O N4 35Cl 32S9
0.59 ppm
593.71423
C3 H O15 N3 F 32S8
-1.61 ppm
611.72570
C3 O14 N F6 32S7
-0.89 ppm
225.19569
C13 H25 O N2
-1.98 ppm
363.67961
889.30639
C30 H56 O13 N8 F3 32S3
-1.31 ppm
1188.41524
1258.40193
1508.90314
1716.25391
Relative Abundance
m/z

AS4

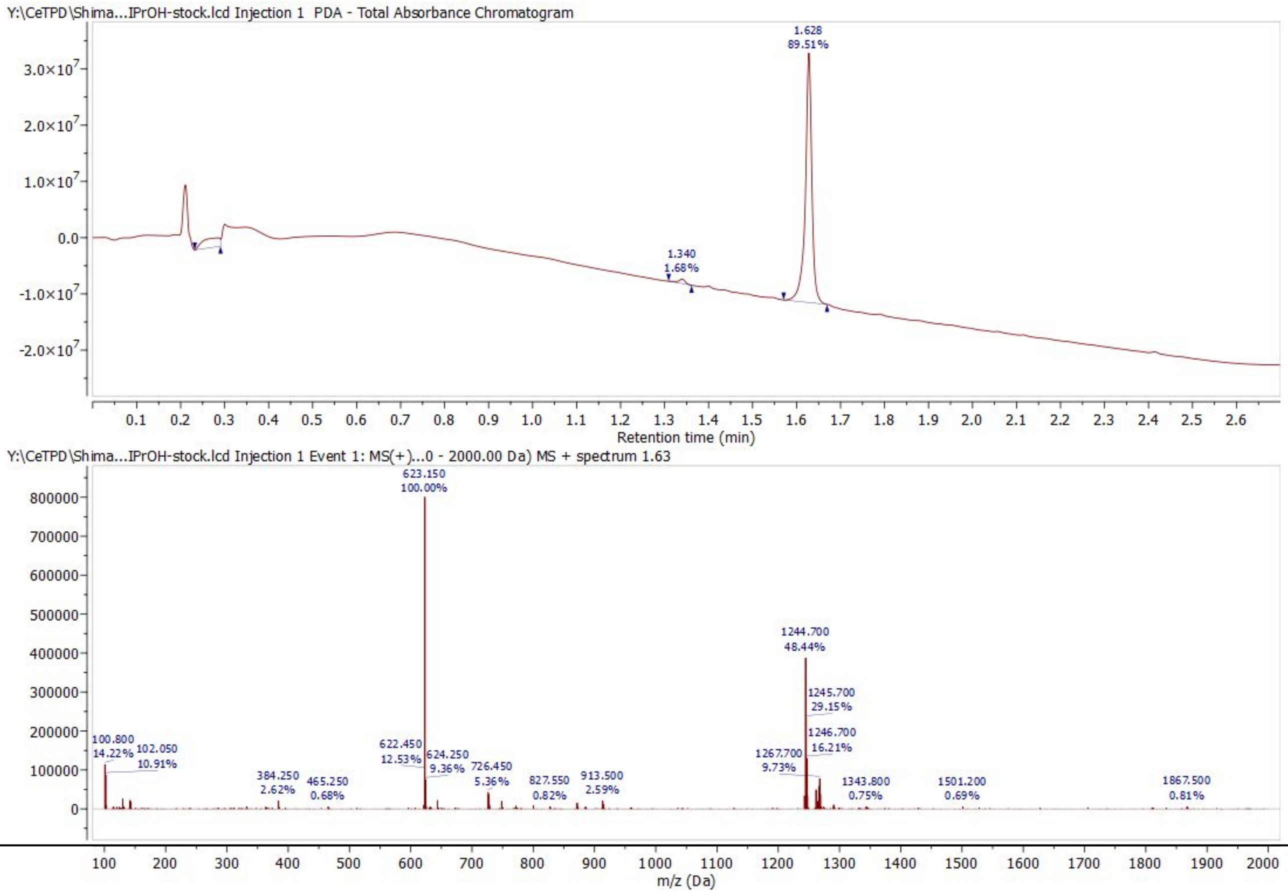

Y:\CeTPD\Shima...IPrOH-stock.lcd Injection 1 PDA - Total Absorbance Chromatogram
1.628
89.51%
1.340
1.68%
Retention time (min)
Y:\CeTPD\Shima...IPrOH-stock.lcd Injection 1 Event 1: MS(+)...0 - 2000.00 Da) MS + spectrum 1.63
623.150
100.00%
1244.700
48.44%
1245.700
29.15%
1246.700
16.21%
1267.700
9.73%
100.800
14.22%
102.050
10.91%
622.450
12.53%
624.250
9.36%
726.450
5.36%
384.250
2.62%
465.250
0.68%
827.550
0.82%
913.500
2.59%
1343.800
0.75%
1501.200
0.69%
1867.500
0.81%
m/z (Da)

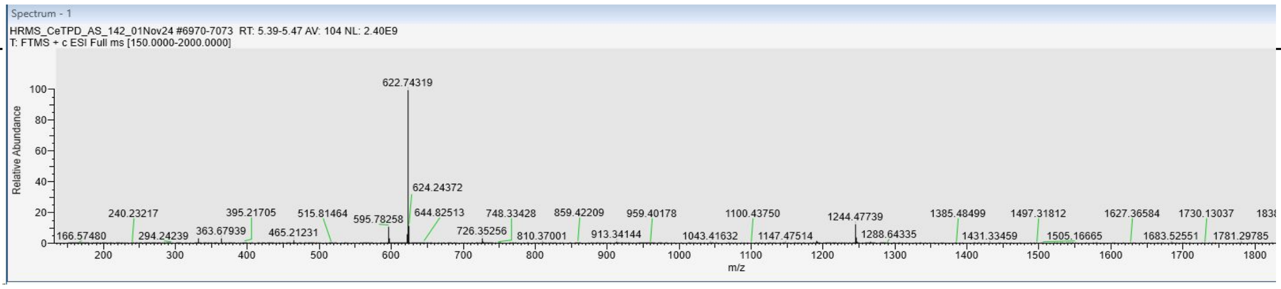

Spectrum - 1
HRMS_CeTPD_AS_142_01Nov24 #6970-7073 RT: 5.39-5.47 AV: 104 NL: 2.40E9
T: FTMS + c ESI Full ms [150.0000-2000.0000]
Relative Abundance
622.74319
624.24372
240.23217
395.21705
515.81464
595.78258
644.82513
748.33428
859.42209
959.40178
1100.43750
1244.47739
1385.48499
1497.31812
1627.36584
1730.13037
166.57480
294.24239
363.67939
465.21231
726.35256
810.37001
913.34144
1043.41632
1147.47514
1288.64335
1431.33459
1505.16665
1683.52551
1781.29785
m/z

AS5

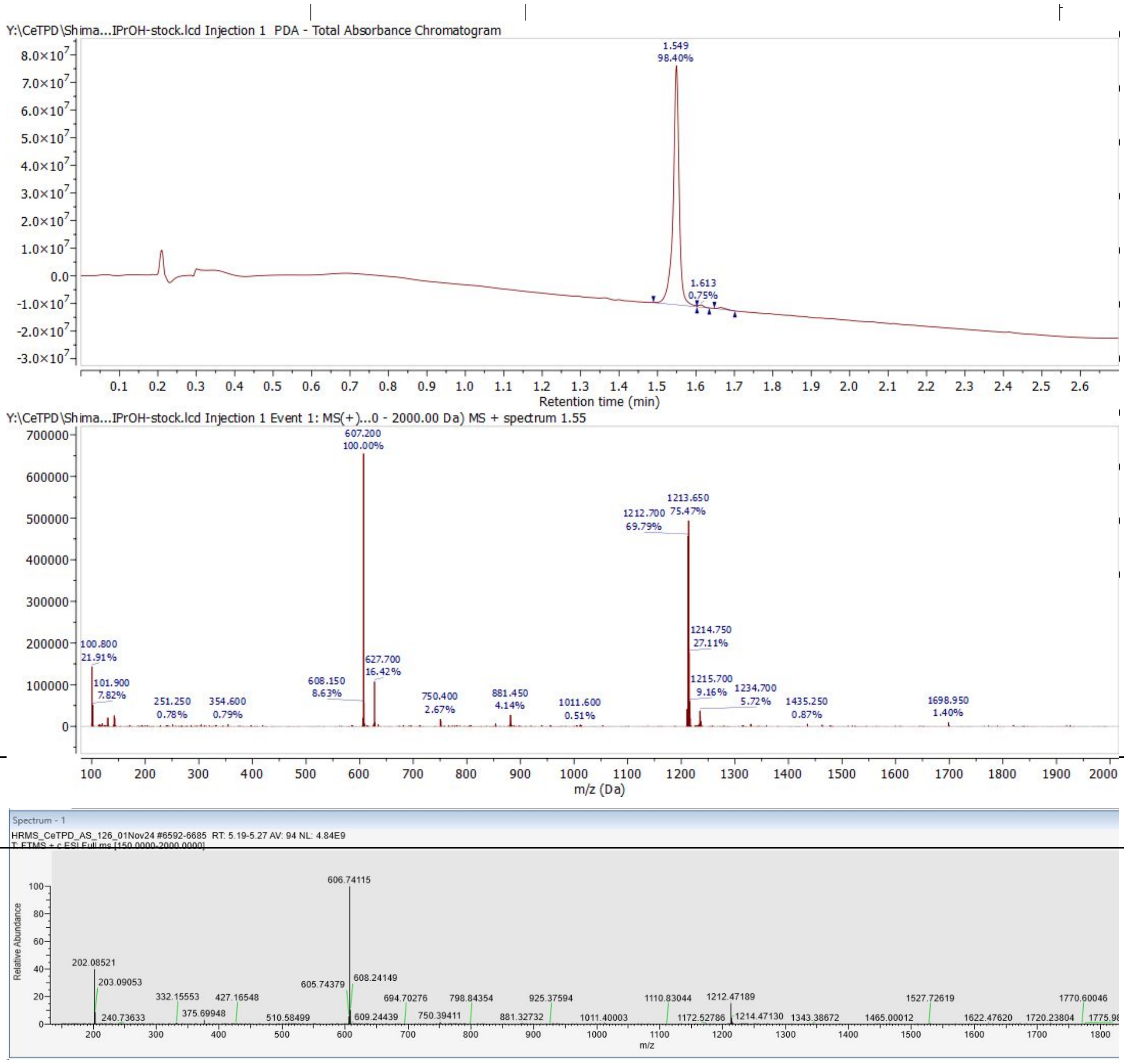

Y:\CeTPD\Shima...IPrOH-stock.lcd Injection 1 PDA - Total Absorbance Chromatogram
1.549
98.40%
1.613
0.75%
Retention time (min)
Y:\CeTPD\Shima...IPrOH-stock.lcd Injection 1 Event 1: MS(+)...0 - 2000.00 Da) MS + spectrum 1.55
607.200
100.00%
1213.650
75.47%
1212.700
69.79%
1214.750
27.11%
100.800
21.91%
627.700
16.42%
101.900
7.82%
608.150
8.63%
1215.700
9.16%
1234.700
5.72%
m/z (Da)
Spectrum - 1
HRMS_CeTPD_AS_126_01Nov24 #6592-6685 RT: 5.19-5.27 AV: 94 NL: 4.84E9
606.74115
202.08521
608.24149
1212.47189
Relative Abundance
m/z

AS6

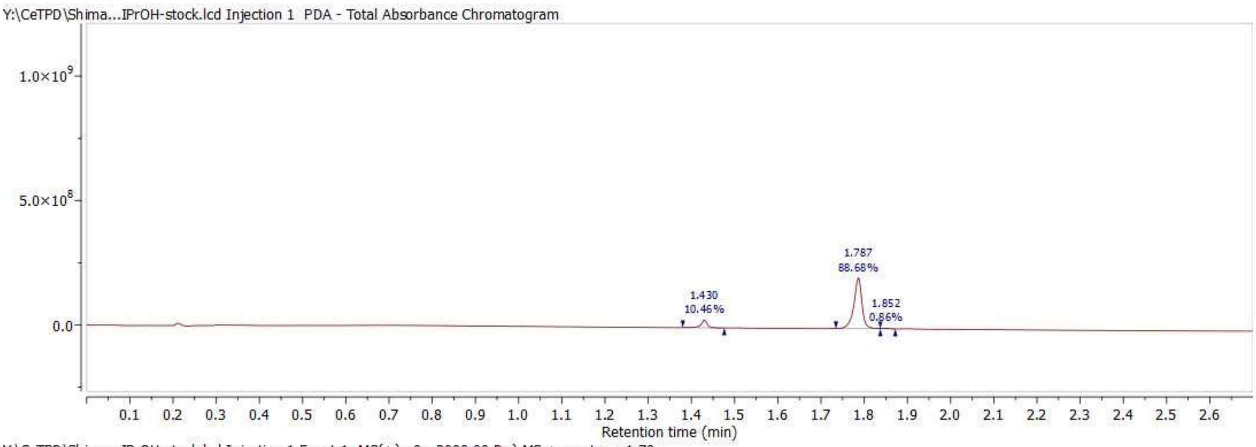

Y:\CeTPD\Shima...IPrOH-stock.lcd Injection 1 PDA - Total Absorbance Chromatogram
1.787
88.68%
1.430
10.46%
1.852
0.86%
Retention time (min)

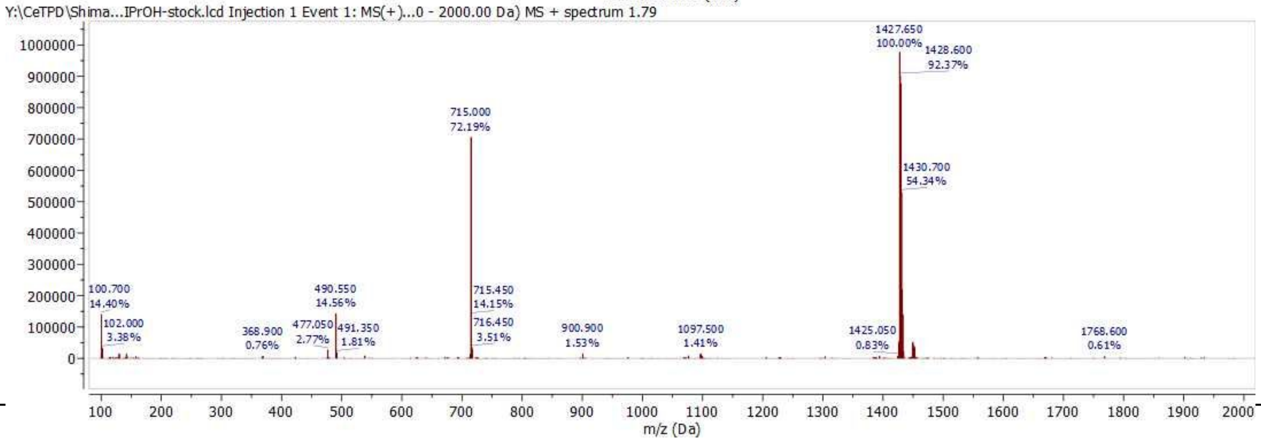

Y:\CeTPD\Shima...IPrOH-stock.lcd Injection 1 Event 1: MS(+)...0 - 2000.00 Da) MS + spectrum 1.79
1427.650
100.00%
1428.600
92.37%
715.000
72.19%
1430.700
54.34%
m/z (Da)

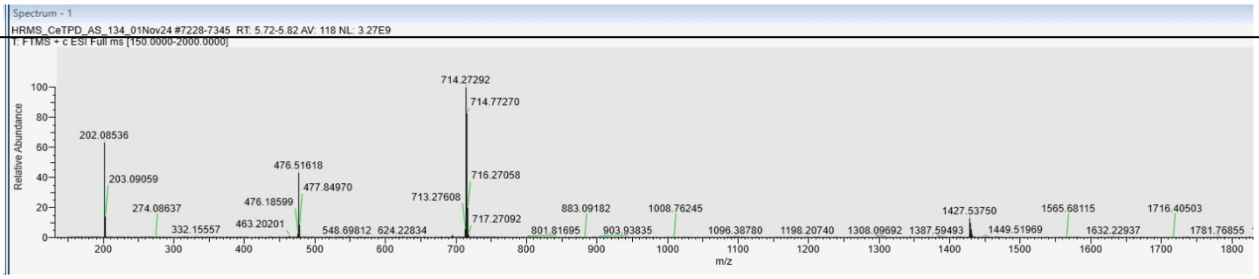

Spectrum - 1
HRMS_CeTPD_AS_134_01Nov24 #7228-7345 RT: 5.72-5.82 AV: 118 NL: 3.27E9
T: FTMS + c ESI Full ms [150.0000-2000.0000]
714.27292
714.77270
202.08536
Relative Abundance
m/z

AS7

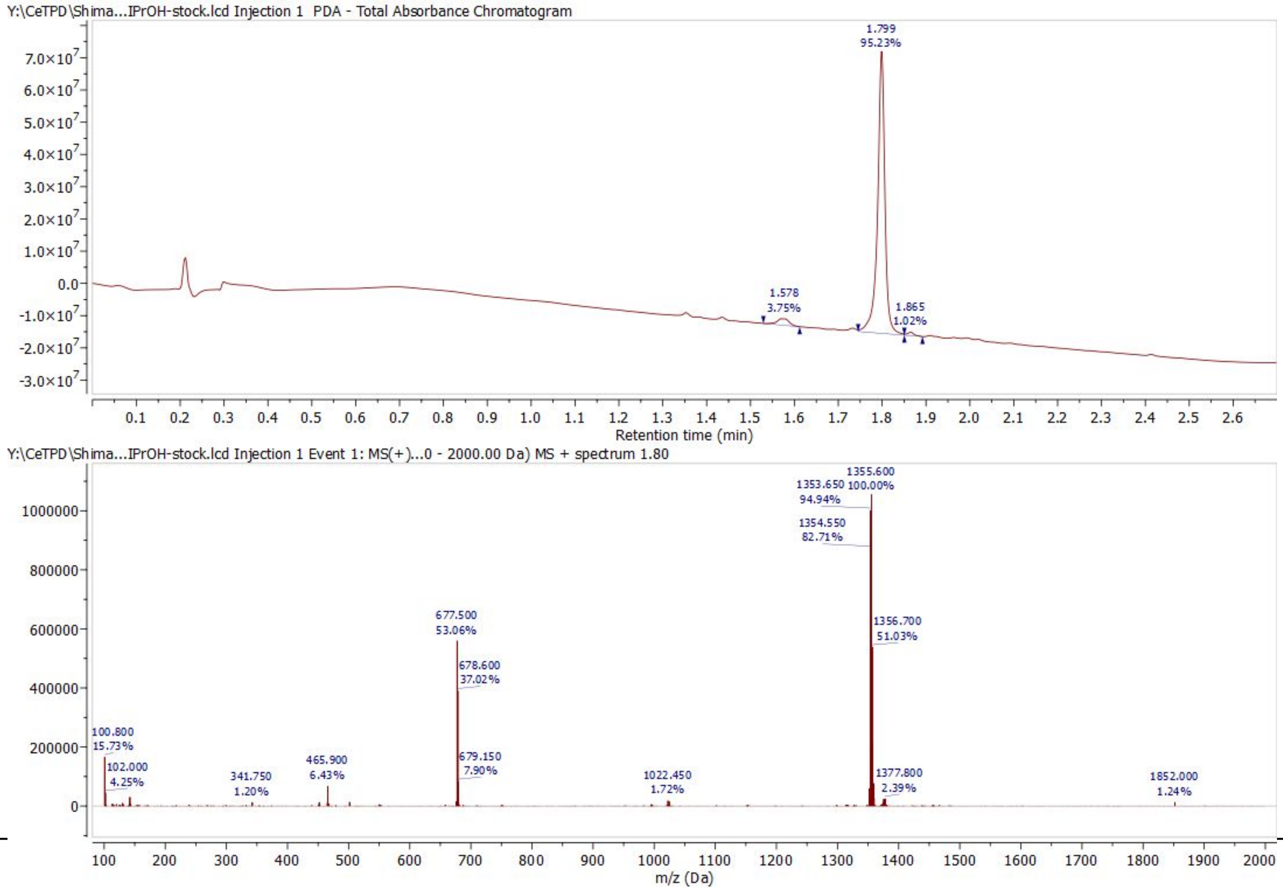
Y:\CeTPD\Shima...IPrOH-stock.lcd Injection 1 PDA - Total Absorbance Chromatogram
1.799
95.23%
1.578
3.75%
1.865
1.02%
Retention time (min)
Y:\CeTPD\Shima...IPrOH-stock.lcd Injection 1 Event 1: MS(+)...0 - 2000.00 Da) MS + spectrum 1.80
1355.600 100.00%
1353.650 94.94%
1354.550 82.71%
1356.700 51.03%
677.500 53.06%
678.600 37.02%
679.150 7.90%
100.800 15.73%
102.000 4.25%
341.750 1.20%
465.900 6.43%
1022.450 1.72%
1377.800 2.39%
1852.000 1.24%
m/z (Da)

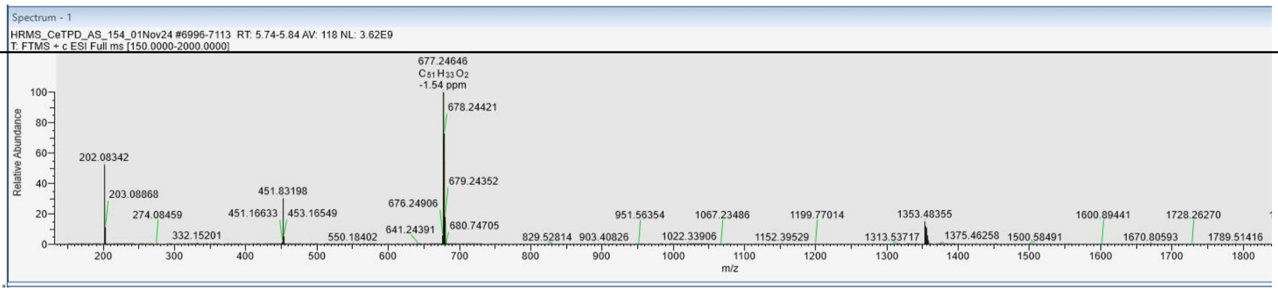
Spectrum - 1
HRMS_CeTPD_AS_154_01Nov24 #6996-7113 RT: 5.74-5.84 AV: 118 NL: 3.62E9
T: FTMS + c ESI Full ms [150.0000-2000.0000]
677.24646
C51 H33 O2
-1.54 ppm
678.24421
679.24352
202.08342
203.08868
274.08459
332.15201
451.83198
451.16633
453.16549
550.18402
641.24391
676.24906
680.74705
829.52814
903.40826
951.56354
1022.33906
1067.23486
1152.39529
1199.77014
1313.53717
1353.48355
1375.46258
1500.58491
1600.89441
1670.80593
1728.26270
1789.51416
Relative Abundance
m/z

References

1. Hu, J.; Hu, B.; Wang, M.; Xu, F.; Miao, B.; Yang, C.-Y.; Wang, M.; Liu, Z.; Hayes, D. F.; Chinnaswamy, K., Discovery of ERD-308 as a highly potent proteolysis targeting chimera (PROTAC) degrader of estrogen receptor (ER). *Journal of medicinal chemistry* **2019,** *62* (3), 1420-1442.
2. Galdeano, C.; Gadd, M. S.; Soares, P.; Scaffidi, S.; Van Molle, I.; Birced, I.; Hewitt, S.; Dias, D. M.; Ciulli, A., Structure-guided design and optimization of small molecules targeting the protein–protein interaction between the von Hippel–Lindau (VHL) E3 ubiquitin ligase and the hypoxia inducible factor (HIF) alpha subunit with in vitro nanomolar affinities. *Journal of Medicinal Chemistry* **2014,** *57* (20), 8657-8663.
3. Maniaci, C.; Hughes, S. J.; Testa, A.; Chen, W.; Lamont, D. J.; Rocha, S.; Alessi, D. R.; Romeo, R.; Ciulli, A., Homo-PROTACs: bivalent small-molecule dimerizers of the VHL E3 ubiquitin ligase to induce self-degradation. *Nature Communications* **2017,** *8* (1), 830.
4. Diehl, C. J.; Salerno, A.; Ciulli, A., Ternary Complex-Templated Dynamic Combinatorial Chemistry for the Selection and Identification of Homo-PROTACs. *Angewandte Chemie International Edition* **2024**, e202319456.
5. Girardini, M.; Maniaci, C.; Hughes, S. J.; Testa, A.; Ciulli, A., Cereblon versus VHL: Hijacking E3 ligases against each other using PROTACs. *Bioorganic & Medicinal Chemistry* **2019,** *27* (12), 2466-2479.
6. Zengerle, M.; Chan, K.-H.; Ciulli, A., Selective small molecule induced degradation of the BET bromodomain protein BRD4. *ACS chemical biology* **2015,** *10* (8), 1770-1777.
7. Petrov, A. R.; Jess, K.; Freytag, M.; Jones, P. G.; Tamm, M., Large-scale preparation of 1, 1′-ferrocenedicarboxylic acid, a key compound for the synthesis of 1, 1′-disubstituted ferrocene derivatives. *Organometallics* **2013,** *32* (20), 5946-5954.
